# Resistance to Tomato Yellow Leaf Curl Virus in Tomato Germplasm

**DOI:** 10.3389/fpls.2018.01198

**Published:** 2018-08-20

**Authors:** Zhe Yan, Ana Pérez-de-Castro, Maria J. Díez, Samuel F. Hutton, Richard G. F. Visser, Anne-Marie A. Wolters, Yuling Bai, Junming Li

**Affiliations:** ^1^The Institute of Vegetables and Flowers, Chinese Academy of Agricultural Sciences, Beijing, China; ^2^Plant Breeding, Graduate School Experimental Plant Sciences, Wageningen University & Research, Wageningen, Netherlands; ^3^Instituto Universitario de Conservación y Mejora de la Agrodiversidad Valenciana, Ciudad Politécnica de la Innovación, Universitat Politècnica de València, Valencia, Spain; ^4^Gulf Coast Research and Education Center, University of Florida, Gainesville, FL, United States

**Keywords:** *Begomovirus*, resistance, *Solanum lycopersicum*, *S. chilense*, *S. peruvianum*, tomato, TYLCV

## Abstract

Tomato yellow leaf curl virus (TYLCV) is a virus species causing epidemics in tomato (*Solanum lycopersicum*) worldwide. Many efforts have been focused on identification of resistance sources by screening wild tomato species. In many cases, the accession numbers were either not provided in publications or not provided in a consistent manner, which led to redundant screenings. In the current study, we summarized efforts on the screenings of wild tomato species for TYLCV resistance from various publications. In addition, we screened 708 accessions from 13 wild tomato species using different inoculation assays (i.e., whitefly natural infection and Agrobacterium-mediated inoculation) from which 138 accessions exhibited no tomato yellow leaf curl disease (TYLCD) symptoms. These symptomless accessions include 14 accessions from *S. arcanum*, 43 from *S. chilense*, 1 from *S. chmielewskii*, 28 from *S. corneliomulleri*, 5 from *S. habrochaites*, 4 from *S. huaylasense*, 2 from *S. neorickii*, 1 from *S. pennellii*, 39 from *S. peruvianum*, and 1 from *S. pimpinellifolium*. Most of the screened *S. chilense* accessions remained symptomless. Many symptomless accessions were also identified in *S. arcanum*, *S. corneliomulleri*, and *S. peruvianum*. A large number of *S. pimpinellifolium* accessions were screened. However, almost all of the tested accessions showed TYLCD symptoms. Further, we studied allelic variation of the *Ty-1*/*Ty-3* gene in few *S. chilense* accessions by applying virus-induced gene silencing and allele mining, leading to identification of a number of allele-specific polymorphisms. Taken together, we present a comprehensive overview on TYLCV resistance and susceptibility in wild tomato germplasm, and demonstrate how to study allelic variants of the cloned *Ty*-genes in TYLCV-resistant accessions.

## Introduction

Tomato yellow leaf curl disease (TYLCD) has been a global constraint to tomato (*Solanum lycopersicum*) production since the 1980s (Moriones and Navas-Castillo, 2000). Up till now, TYLCD is still one of the most devastating diseases of tomato. Infected susceptible tomato plants show symptoms that include yellowing, curling, and cupping of leaves, severe stunting and abortion of flowers and fruits, all of which can lead to yield reduction of up to 100% (Abhary et al., 2007). TYLCD can be caused by a cluster of related virus species including tomato yellow leaf curl virus (TYLCV), which belongs to the genus *Begomovirus* of the *Geminiviridae* family. TYLCV has a wide host range that includes tomato (*S. lycopersicum*), sweet pepper (*Capsicum annuum*), chili pepper (*C. chinense*), tobacco (*Nicotiana tabacum*), common bean (*Phaseolus vulgaris*), petunia (*Petunia* × *hybrida*), and lisianthus (*Eustoma grandiflora*) (Díaz-Pendón et al., 2010).

In nature, TYLCV is transmitted exclusively by the sweet potato whitefly *Bemisia tabaci* (Genn.) in a persistent-circulative manner (Gronenborn, 2007). *B. tabaci* is an invasive pest with global importance since more than 175 countries officially report the presence of *B. tabaci* (CABI 2017; *Bemisia tabaci*. In: Invasive Species Compendium^1^). *B. tabaci* is a complex consisting of at least 24 distinct species (De Barro et al., 2011). The *Bemisia* Middle East-Asia Minor 1 (MEAM1/B) and Mediterranean (MED/Q) are regarded as the most invasive and damaging species, and these are also the species that transmit TYLCV to tomato (De Barro et al., 2011; Ning et al., 2015). Serious damage in tomato production attributed to TYLCV was first reported in Israel in 1959 (Cohen and Antignus, 1994). Since then, the list of tomato production regions reporting TYLCV epidemics has grown (Barboza et al., 2014; Chinnaraja et al., 2016; Mabvakure et al., 2016). Among the countries for which *B. tabaci* incidence was reported, more than 70 also reported the occurrence of TYLCV [CABI 2016; TYLCV (leaf curl). In: Invasive Species Compendium^2^].

Domesticated tomato is known to be vulnerable to TYLCV infection, but resistance exists in wild tomato species (Ji et al., 2007b). Accessions exhibiting no TYLCD symptoms upon infection have been reported in a number of species, including *S. arcanum*, *S. cheesmaniae*, *S. chilense, S. chmielewskii*, *S. corneliomulleri*, *S. galapagense*, *S. habrochaites*, *S. neorickii*, *S. pennellii*, *S. peruvianum*, and *S. pimpinellifolium* (Ji et al., 2007b; Vidavski et al., 2008; De la Peña et al., 2010; Pereira-Carvalho et al., 2010; Tomás et al., 2011). So far, three TYLCV resistance genes have been cloned. *Ty-1* and *Ty-3* originate from *S. chilense* accession LA1969 and LA2779, respectively (Zamir et al., 1994; Ji et al., 2007a). They are two alleles of the same gene that is located on the long arm of tomato chromosome 6 and encodes an RNA-dependent RNA polymerase (RDR) (Verlaan et al., 2011, 2013). *Ty-2*, originating from *S. habrochaites* accession B6013 and located on the long arm of chromosome 11, encodes an NB-LRR gene (Yang et al., 2014; Yamaguchi et al., 2018). *ty-5* is a loss-of-function mutant allele of the *pelota* gene located on chromosome 4. The mutation in *ty-5* is caused by a T-to-G transversion in the coding region, which occured in cultivated tomato (Lapidot et al., 2015). However, it has also been suggested that *ty-5* is derived from a complex of *S. peruvianum* accessions (Anbinder et al., 2009). In addition to these cloned genes, two resistance loci *Ty-4* and *Ty-6* have been mapped. *Ty-4* is identified from *S. chilense* LA1932. This locus is located on the long arm of chromosome 3 and has a minor effect toward TYLCV resistance (Ji et al., 2009). *Ty-6* is the most recently identified TYLCV resistance locus on the long arm of chromosome 10, presumably originating from *S. chilense* accessions LA1938 and LA2779 (Hutton and Scott, 2014).

Up till now, introgressions of *Ty-1*, *Ty-2*, and *Ty-3* into cultivated tomato have been the major focus in breeding programs. *Ty-2* based resistance can be overcome by TYLCV-related Tomato yellow leaf curl Sardinia virus (TYLCSV) (Barbieri et al., 2010) and breakdown of *Ty-2* mediated resistance has been demonstrated recently by an isolate of the Mild strain of TYLCV (TYLCV-Mld) (Ohnishi et al., 2016). *Ty-1*-mediated resistance is not suitable to use under high disease pressure which leads to resistance breakage in some cases (García-Cano et al., 2008). Resistance breakage facilitates TYLCD epidemics, which urges plant breeders to continuously search for effective novel sources of resistance in the wild tomato gene pool. In multiple research programs wild tomato germplasm has been screened in order to identify accessions that can be utilized as sources of TYLCV resistance. Although these efforts have resulted in the identification of a number of sources exhibiting no TYLCD symptoms, the accession numbers of these sources were not consistently provided in publications, which led to redundant screenings in some cases. In this article, we summarize the results of previous resistance screening efforts and the use of different resistance resources in tomato introgression breeding. In addition, we report the identification of 138 tomato accessions that were TYLCD symptomless in a large-scale screening of 708 accessions from 13 wild tomato species. Finally, we discuss the potential use of the newly identified resources for TYLCV resistance in tomato breeding, in the context of donor species, the cloned *Ty* genes and viral titer levels.

## Materials and Methods

### Plant Materials

Wild tomato accessions were collected from Tomato Genetics Resource Center (TGRC), World Vegetable Center in Taiwan (previously the Asian Vegetable Research and Development Center, AVRDC), Centre for Genetic Resources, Netherlands (CGN), and Kentucky State University (KSU). The tomato cultivar *S. lycopersicum* cv. Moneymaker (MM) was included as susceptible control.

### Whitefly-Mediated Natural Inoculation

Germplasm accessions were screened from July till September in the years 2012, 2013, and 2014 for TYLCD resistance using field assays and natural infection with whiteflies at the Institute of Vegetables and Flowers, Chinese Academy of Agricultural Sciences, Beijing. Seeds were germinated in petri-dishes on moist sterilized filter paper. Germinated seedlings were transferred to pots and placed into a plastic tunnel greenhouse. After transplanting, plants were continuously exposed to natural infection of whiteflies by opening the tunnel ventilation. From July till September in Beijing, China, natural incidence of whitefly with large vector population facilitates TYLCD epidemics. Experimental design was a randomized complete block design with two blocks and one to eight plants per plot depending on the number of available germinated seedlings. *S. lycopersicum* cv. MM was included as susceptible control and used in each block (five plants per block). An inbred line, TO-937 derived from *S. pimpinellifolium* material, was included in each block as a tolerant control.

### Agrobacterium-Mediated Inoculation

For TYLCV disease assay using Agrobacterium-mediated inoculation in Wageningen, Netherlands, an infectious TYLCV-IL clone (pTYCz40a) was used. The method has been described in detail by Verlaan et al. (2011). The full length clone of TYLCV-IL genome was maintained in *Agrobacterium tumefaciens* strain LBA4404. Agrobacterium culture was grown, centrifuged and the pellet resuspended to OD_600_ = 0.5. Tomato plants used to screen for TYLCV resistance were grown under greenhouse conditions. The greenhouse was maintained at 23°C, 60% humidity and a 16/8 h day/night cycle. TYLCV Agro-inoculation was performed on plants at approximately three true leaves stage (around 21 days after sowing) as described by Verlaan et al. (2011). For most of the tested wild species, four plants per accession were inoculated and two plants were mock-inoculated. Only for *S. peruvianum*, *S. chilense*, and *S. lycopersicum* var. *cerasiforme* accessions, eight plants per accession were inoculated with the virus and four plants were mock-inoculated.

### Disease Assessment

In China, plant responses were evaluated three times at 8, 10, and 12 weeks after sowing for TYLCD symptom development. On each date, each plant was rated using a 0 to 4 disease severity index (DSI) described by Friedmann et al. (1998), where 0 indicates no TYLCD symptoms, and 4 means severe TYLCD symptoms, remarkable yellowing, curling leaves and significant stunting in plant size. Intermediate scores, 0.5, 1.5, 2.5, and 3.5 were incorporated for more precise disease severity scoring. The final disease score of each accession was taken into account and the value was presented as mean of all the tested individuals. Only accessions that remained asymptomatic throughout the whole evaluation period have been regarded as resistant genotypes. At Wageningen, plants were scored for symptom development at 25, 35, 45, and 55 days after virus inoculation. Symptom severity was scored using the same scale as described above. Final results were the disease scores of the last evaluation at 55 days post inoculation. At 45 days after TYLCV inoculation, top young leaves were harvested for DNA isolation using cetyltrimethyl ammonium bromide (CTAB) based protocol (Fulton et al., 1995). The presence of TYLCV was detected by PCR using primers TYLCV-Picó-F and TYLCV-Picó-R as described by Picó et al. (1999a).

### Virus-Induced Gene Silencing (VIGS) and Allele Mining

We used the virus-induced gene silencing (VIGS) construct targeting *Ty-1*/*Ty-3* and followed the agro-infiltration procedures as described in Verlaan et al. (2013). To quantify viral titer and obtain full length cDNA sequences of the *Ty-1*/*Ty-3* alleles, top leaves of plants infiltrated with VIGS constructs followed by TYLCV infection were harvested and grinded in liquid nitrogen using mortar and pestle. Total DNA was isolated using the CTAB based protocol (Fulton et al., 1995). For quantification of virus accumulation, the forward primer TYLCV-IS 1678F and the reverse primer TYLCV-CONS 1756R were used as described by Powell et al. (2012). *Elongation factor 1α* (*EF*) gene was used as a reference with primers: EF-F (5′-ATTGGAAACGGATATGCCCCT-3′) and EF-R (5′-TCCTTACCTGAACGCCTGTCA-3′). The amounts of viral DNA were calculated using the ΔΔCt method as described by Livak and Schmittgen (2001). Quantitative real-time PCR (qPCR) was carried in 10 μl reactions with a Bio-Rad iCycler iQ5 using SYBR Green Supermix according to the manufacturer’s protocol (Bio-Rad). Total RNA was extracted by using the RNeasy Plant Mini Kit (Qiagen) following the manufacturer’s protocol. DNase treatment was performed on 1 μg RNA (DNase I Amp. Grade) as described by the manufacturer (Invitrogen) and cDNA was synthesized using the iScript cDNA Synthesis Kit following the protocol (Bio-Rad).

For allele mining, full length cDNA sequences of the *Ty-1*/*Ty-3* gene were obtained by PCR using a high-fidelity Phusion DNA polymerase (Thermo Fisher Scientific) with primers Ty-F7 and Ty-R5 (Verlaan et al., 2013). cDNA amplification was carried out with 30 cycles of denaturation (10 s at 98°C), annealing (30 s at 68°C), and extension (2 min at 72°C) followed by 7 min of further extension at 72°C. Blunt ended PCR products were cloned using the Zero Blunt PCR Cloning Kit (Invitrogen). After ligation, the constructs were transformed into competent *Escherichia* *coli* One Shot^®^ TOP10 cells (Invitrogen). Recombinant clones were selected based on colony PCR using primers M13F (5′-GTAAAACGACGGCCAG-3′) and primer R7 that hybridizes within the insert (Verlaan et al., 2013). Plasmids of positive colonies were isolated using the QIAprep Spin Miniprep Kit (Qiagen). Plasmids were sequenced with different primer combinations covering the full length of the *Ty-1*/*Ty-3* gene (M13F, F3, F7, F6, F4, and M13R) (Verlaan et al., 2013). Sequences were analyzed using SeqMan Pro 9 (DNA Star). The cDNA sequences of the *Ty-1*/*Ty-3* alleles in MM, *S. chilense* LA1969 and *S. chilense* LA2779 were obtained from Verlaan et al. (2013); *S. chilense* LA1932, LA1938, and LA1971 from Caro et al. (2015). Alignments were made with MegAlign (DNA Star).

## Results

### Evaluation of Wild Tomato Species for TYLCD Resistance

We tested 701 accessions from 13 wild tomato species for resistance to TYLCD. The species that were represented in this screen included *S. arcanum* (24 accessions), *S. cheesmaniae* (7 accessions), *S. chilense* (51 accessions), *S. chmielewskii* (3 accessions), *S. corneliomulleri* (44 accessions), *S. galapagense* (2 accessions), *S. habrochaites* (36 accessions), *S. huaylasense* (4 accessions), *S. lycopersicoides* (2 accessions), *S. neorickii* (3 accessions), *S. pennellii* (38 accessions), *S. peruvianum* (81 accessions), and *S. pimpinellifolium* (406 accessions) (**Supplementary Table S1**).

**FIGURE 1:**
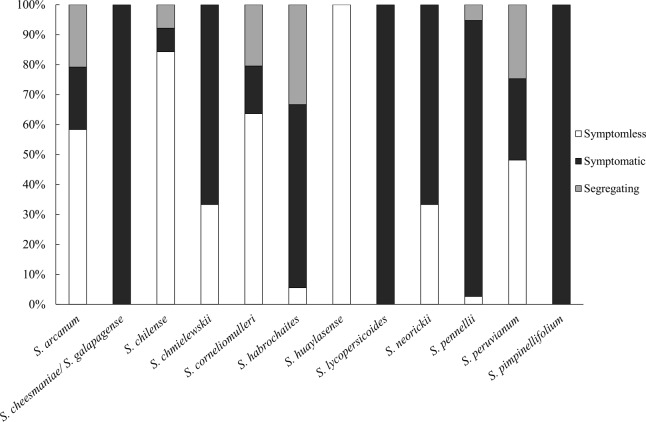
Stack bar graph depicting percentage of accessions for each *Solanum* spp. that exhibited symptomatic, symptomless, or segregating phenotypes upon TYLCD infection. Taxonomy of wild tomato relatives follow the classification system as presented in Peralta et al. (2008).

These accessions were subjected to a field screening with natural whitefly infection. To eliminate phenotypic variations and to assess uniformity of the infection across the trial, the susceptible control *S. lycopersicum* cv. MM plants were placed randomly at different positions within the experimental block. Meanwhile, an inbred line, TO-937 derived from *S. pimpinellifolium* material was included as a tolerant control. TO-937 has the introgression of type IV leaf glandular trichomes which limits whitefly (*B. tabaci*) access and feeding (Rodríguez-López et al., 2011). All MM plants exhibited a homogenous and highly susceptible response (DSI of 4). All plants of line TO-937 showed clear TYLCD symptoms (average DSI of 3). The wild accessions screened in the present study showed a range of phenotypic reactions to TYLCD infection. For simplicity, phenotypic responses to TYLCD were initially categorized into two groups, symptomless and symptomatic. Among the accessions tested, we identified 133 symptomless accessions (**Figure 1** and **Supplementary Table S1**). Proportionally, *S. huaylasense* and *S. chilense* displayed the most resistant accessions, with all four *S. huaylasense* accessions tested and more than 80% of the *S. chilense* accessions tested exhibiting no TYLCD symptoms (**Figure 1**). Among *S. chilense* accessions, four of the 54 screened accessions were symptomatic showing mild symptoms (**Supplementary Table S2**). From species of *S. arcanum*, *S. corneliomulleri*, and *S. peruvianum* a large number of symptomless accessions were identified. Of these three species, symptomatic accessions demonstrated mild to moderate levels of symptoms and severe symptoms were observed only in accessions LA1350 (*S. arcanum*), PI 199380 (*S. corneliomulleri*), and LA3218 (*S. peruvianum*) (**Supplementary Table S2**).

In contrast, the majority of *S. pennellii* and *S. habrochaites* accessions (92 and 61%, respectively) showed clear viral symptoms with severe yellowing and curling (**Figure 1**, **Supplementary Figure S1** and **Supplementary Table S2**). From *S. cheesmaniae*, *S. galapagense*, *S. lycopersicoides*, and *S. pimpinellifolium* all the tested accessions displayed TYLCD symptoms (**Figure 1**, **Supplementary Figure S1** and **Supplementary Table S2**). Particularly, all the 406 *S. pimpinellifolium* accessions showed TYLCD symptoms (**Figure 1** and **Supplementary Table S2**) although symptom severity varied significantly among accessions. Mild TYLCD symptoms, slight yellowing and curling of the young leaves, were observed only in some accessions including LA1607, LA1344, LA2578, LA1863, LA0398, LA1630, and LA1589 (**Supplementary Table S2**).

Significant plant-to-plant variation in phenotypic responses was observed within several accessions of tomato wild species, including *S. arcanum* (5 accessions), *S. chilense* (4 accessions), *S. corneliomulleri* (9 accessions), *S. habrochaites* (12 accessions), *S. pennellii* (2 accessions), and *S. peruvianum* (20 accessions) (**Figure 1** and **Supplementary Table S3**). This variation may be due to actual segregation of resistance alleles resulting from the heterogeneous nature of these out-crossing species (Peralta and Spooner, 2005; Moyle, 2008). Alternatively, it may also be due to the presence of escapes, since this test was conducted using natural infection in the field.

At Wageningen, 11 different wild tomato species (including one *S. lycopersicum* line as control) were screened and evaluated for their responses to TYLCV using Agrobacterium-mediated inoculation (**Table 1**). This screening panel included 32 accessions of the following species, *S. arcanum* (1 accession), *S. cheesmaniae* (2 accessions), *S. chilense* (1 accession), *S. corneliomulleri* (1 accession), *S. galapagense* (1 accession), *S. habrochaites* (10 accessions), *S. lycopersicoides* (1 accession), *S. neorickii* (1 accession), *S. peruvianum* (11 accessions), and *S. pimpinellifolium* (3 accessions). Twenty out of the 32 tested accessions showed no TYLCD symptoms (**Table 1**). Plants of *S. arcanum* LA2172, *S. chilense* LA0458, *S. corneliomulleri* CGN14358, *S. lycopersicoides* CGN23973, *S. neorickii* LA0735, and all the tested accessions of *S. peruvianum* were completely free of symptoms. Plants of *S. habrochaites* accessions LA4137, LA1777, and LA1718 also exhibited no TYLCV symptoms, while plants of the other tested *S. habrochaites* accessions showed severe symptoms. *S. pimpinellifolium* accession CGN15528 did not show viral symptoms. The other tested accessions (LA1584 and PI 365967) of *S. pimpinellifolium* exhibited clear TYLCV symptoms with yellowing and curling of the young leaves. Plants of the *S. galapagense* accession and *S. cheesmaniae* accession LA1409 were also susceptible to TYLCV. *S. cheesmaniae* accession PI 266375 segregated for its response to TYLCV infection. Of the 10 individuals tested, two plants exhibited no TYLCV symptoms, while the rest displayed a mild to moderate level of susceptibility (**Table 1**). Among 20 TYLCV symptomless accessions, 14 accessions were tested for virus status. Viral DNA was detected in the top young leaves of all the tested plants (**Supplementary Table S4**).

**Table 1:** Average disease severity index of wild tomato species screened for resistance to Tomato Yellow Leaf Curl Virus (TYLCV) using Agrobacterium-mediated inoculation with an infectious TYLCV-IL clone at Plant Breeding, Wageningen University & Research, Netherlands.

*Solanum* spp. accession^a^	DSI^b^	*Solanum* spp. accession^a^	DSI^b^
*S. arcanum*		*S. lycopersicoides*	
LA2172^*c*^	0 ± 0	CGN23973	0 ± 0
*S. cheesmaniae*		*S. lycopersicum* var. *cerasiforme*	
PI 266375^*c*^	2 ± 0.5	LA1310	4 ± 0
LA1409	4 ± 0	*S. neorickii*	
*S. chilense*		LA0735^*c*^	0 ± 0
LA0458	0 ± 0	*S. peruvianum*	
*S. corneliomulleri*		CGN14355	0 ± 0
CGN14358	0 ± 0	CGN14356	0 ± 0
*S. galapagense*		CGN15530^*c*^	0 ± 0
LA1401^*c*^	4 ± 0	CGN15531	0 ± 0
*S. habrochaites*		CGN15532^*c*^	0 ± 0
LA1718^*c*^	0 ± 0	LA0372	0 ± 0
LA1777^*c*^	0 ± 0	LA0462	0 ± 0
LA4137	0 ± 0	LA1955	0 ± 0
LA2314	3.2 ± 0.4	LA1977	0 ± 0
CGN15790	4 ± 0	LA4125	0 ± 0
CGN15791^*c*^	4 ± 0	PI 126928	0 ± 0
CGN15792^*c*^	4 ± 0	*S. pimpinellifolium*	
CGN15879	4 ± 0	CGN15528	0 ± 0
CGN24035	4 ± 0	LA1584	4 ± 0
PI 134417	4 ± 0	PI 365967	4 ± 0

*^a^Taxon using the classification system of CGN numbers according to gene bank records in the Netherland (Centre for Genetic Resources, Netherlands) (Peralta et al., 2008). ^b^DSI = Disease Severity Index on 0 (symptomless)–4 (severe symptom) scales as described in Friedmann et al. (1998). Results were displayed as Mean DSI ± Standard deviation. ^c^Accessions were included in the 150 Tomato Genome Re-sequencing Project (Aflitos et al., 2014). S. neorickii LA0735 refers to a different number (CGN24193), in the list of selected wild accessions of the 150 Tomato Genome ReSequencing Project. CGN24193 is the accession number published in the genebank, Centre for Genetic Resources, Netherlands.*

### Responses of the Same Tomato Species to TYLCV Infection Using Different Inoculation Methods

Wild tomato accessions have been tested in China and Netherlands using natural whitefly infection and agroinoculation, respectively. Some of the accessions were screened using both inoculation methods leading to similar results (**Table 2**). Plants of *S. arcanum* accession LA2172, *S. chilense* LA0458, *S. corneliomulleri* CGN14358, and six out of the 11 screened *S. peruvianum* accessions (CGN15530, CGN15532, LA0372, LA1977, LA4125, and PI 126928) were free of viral symptoms (**Table 2**). While, plants of *S. cheesmaniae* accession LA1409, six *S. habrochaites* accessions (CGN15790, CGN15791, CGN15792, CGN15879, CGN24035, and PI 134417) and *S. pimpinellifolium* accession LA1584 showed severe symptoms (**Table 2**).

**Table 2:** Disease severity index of TYLCD infection in wild tomato species after artificial Agrobacterium-mediated inoculation and field infection with whitefly.

*Solanum* spp. accession^a^	Field infection	Agroinoculation
	
	Average DSI^b^	Average DSI^b^
*S. arcanum*		
LA2172	0 ± 0	0 ± 0
*S. cheesmaniae*		
PI 266375	0 ± 0	2 ± 0.5
LA1409	3.9 ± 0.2	4 ± 0
*S. chilense*		
LA0458	0 ± 0	0 ± 0
*S. corneliomulleri*		
CGN14358	0 ± 0	0 ± 0
*S. habrochaites*		
LA2314	0 ± 0	3.2 ± 0.4
CGN15790	3 ± 0	4 ± 0
CGN15791	3.5 ± 0.1	4 ± 0
CGN15792	3.1 ± 0.3	4 ± 0
CGN15879	3 ± 1	4 ± 0
CGN24035	3.8 ± 0.3	4 ± 0
PI 134417	2.4 ± 1.3	4 ± 0
*S. lycopersicoides*		
CGN23973	3 ± 0.2	0 ± 0
*S. peruvianum*		
CGN14355	7 (0 ± 0)/5 (1.4 ± 0.4)	0 ± 0
CGN14356	8 (0 ± 0)/3 (1 ± 0)	0 ± 0
CGN15531	9 (0 ± 0)/4 (1 ± 0)	0 ± 0
CGN15530	0 ± 0	0 ± 0
CGN15532	0 ± 0	0 ± 0
LA0372	0 ± 0	0 ± 0
LA0462	2 ± 0	0 ± 0
LA1955	1 ± 0	0 ± 0
LA1977	0 ± 0	0 ± 0
LA4125	0 ± 0	0 ± 0
PI 126928	0 ± 0	0 ± 0
*S. pimpinellifolium*		
LA1584	3.5 ± 0.7	4 ± 0

*^a^Taxon using the classification system of CGN numbers according to gene bank records in the Netherland (Centre for Genetic Resources, Netherlands) (Peralta et al., 2008). ^b^DSI = Disease Severity Index on 0 (symptomless)–4 scales as described in Friedmann et al. (1998). Results were displayed as Mean DSI ± Standard deviation. S. peruvianum accessions CGN14355, CGN14356, and CGN15531 segregated for TYLCV resistance when using whitefly natural inoculation, average DSI refers to two groups of plants, number of symptomless individuals (mean DSI ± Standard deviation) and number of symptomatic plants (mean DSI ± Standard deviation).*

Also, contrasting results of the same accession were observed. *S. habrochaites* LA2314, which was symptomless in the field with whitefly infection, exhibited clear TYLCD symptoms with the agroinfection method (**Table 2**). In contrast, symptomatic accessions in the field test, such as *S. lycopersicoides* CGN23973 as well as *S. peruvianum* LA0462 and LA1955, showed no symptoms using Agrobacterium-mediated inoculation (**Table 2**). Among the accessions that were heterogenic in response to whitefly natural infection, we tested three accessions from *S. peruvianum* (CGN14355, CGN14356, and CGN15531) with Agrobacterium mediated TYLCV inoculation and all the tested plants were free of viral symptoms (**Table 2**).

### Presence of Functional *Ty-1* and *Ty-3* Alleles in Different *S. chilense* Accessions

Previous studies indicated that the resistance to TYLCV in *S. chilense* accessions LA1932, LA1938, LA1960, and LA1971 is due to the presence of functional *Ty-1*/*Ty-3* alleles (Caro et al., 2015). To verify whether the *S. chilense* accessions showing no TYLCD symptom in this study carry a functional *Ty-1*/*Ty-3* allele, VIGS was applied to silence the *Ty-1*/*Ty-3* gene in six *S. chilense* accessions (**Table 3**). These accessions were selected because relatively more seeds were available.

**Table 3:** Silencing of *Ty-1*/*Ty-3* compromises TYLCV resistance in multiple *S. chilense* accessions.

Accession	Silencing construct (TRV2-180)			Control construct (TRV2-GUS)		
	Plants tested^b^	R^a^	S^a^	Plants tested	R^a^	S^a^
LA1961	10	10	0	5	5	0
LA2981	10	10	0	5	5	0
LA0130	9	3	6	5	5	0
LA2737	10	5	5	5	5	0
LA2754	4	4	0	2	2	0
LA1960	10	2	8	5	5	0
*Ty-1* line	10	2	8	5	5	0
Moneymaker	10	0	10	5	0	5

*The TRV2-180 construct was applied to silence Ty-1/Ty-3 as described in Verlaan et al. (2013). TRV2-GUS was used as a negative control construct with insertion of a GUS fragment. ^a^Resistant (R): Symptomless, disease score 0; mild symptom with disease index ranging from 0.5 to 1; Susceptible (S): Moderate to severe symptom, disease index 2 to 4. ^b^For each accession, 10 plants were tested. If the number is smaller than 10, it means that a number of plants died.*

After TYLCV inoculation, susceptible MM plants showed severe TYLCV symptoms 20 days post inoculation with TYLCV (**Table 3**). An advanced breeding line harboring the *Ty-1* gene (named hereafter as the *Ty-1* line) was included as a positive control. Plants of the *Ty-1* line, which were infiltrated with the TRV2-GUS construct remained symptomless. While, eight out of 10 plants of the *Ty-1* line, which were infiltrated with the TRV2-180 construct to silence the *Ty-1* gene, displayed TYLCV symptoms (**Table 3**). However, not all the TRV2-180 infiltrated plants showed symptoms, showing that the silencing effect of VIGS was not uniform.

**FIGURE 2:**
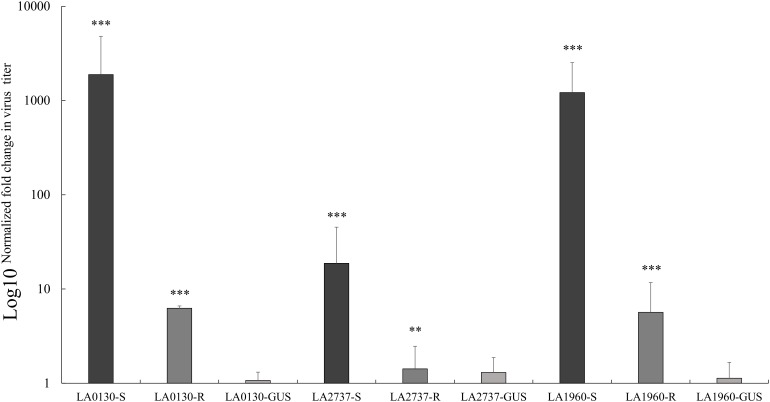
Relative quantification of virus accumulation by qPCR in *S. chilense* accessions LA0130, LA2737, and LA1960 upon silencing *Ty-1*/*Ty-3*. For each accession, tested individuals infiltrated with silencing construct were grouped according to their phenotypic responses; R, resistant, S, compromised resistance. The amounts of viral DNA were normalized using the *Tomato elongation factor 1α* (*EF*). For each accession, the relative level of viral DNA was calibrated to the DNA level in the respective control plants infiltrated with the TRV2-GUS vector. Error bars represent standard deviation of biological replicates. Virus titer was converted into logarithmic scale (log10) displayed on the vertical axis. *Asterisks* indicate significant difference between TRV2-180 plants and average of TRV2-GUS plants according to one way analysis of variance. Number of asterisks indicate the degree of significance (^∗∗^*p* < 0.01 and ^∗∗∗^*p* < 0.001).

All five plants of *S. chilense* accessions LA1961, LA2981, LA0130, LA2737, LA2754, and LA1960 infiltrated with the TRV2-GUS construct remained resistant, demonstrating that TYLCV resistance in these accessions was not influenced by the VIGS vector, tobacco rattle virus (TRV). Of accessions LA0130, LA2737, and LA1960, a number of plants infiltrated with the TRV2-180 construct showed TYLCV symptoms (**Table 3**), indicating that the resistance of these accessions was compromised upon silencing the *Ty-1* gene (**Table 3**). We also quantified virus concentration in susceptible TRV2-180 plants of the accessions LA0130, LA2737, and LA1960 and compared it with that of the resistant TRV2-180 plants (no compromised resistance after silencing) and the TRV2-GUS plants. Significantly more virus accumulation was detected in silenced *Ty-1*/*Ty-3* plants compared with that of the other two groups (**Figure 2**). All the plants of the accessions LA1961 and LA2981 tested with the TRV2-180 construct remained resistant (**Table 3**). Ten plants of the accession LA2754 were infiltrated with TRV2-180 construct and six died. The remaining ones did not show symptoms after TYLCV inoculation (**Table 3**).

### Intraspecific Sequence Variations in *Ty-1*/*Ty-3* Resistant Alleles

To explore *Ty-1*/*Ty-3* allele-specific polymorphisms, the sequences of the coding regions from *S. chilense* accessions LA0130, LA2737, and LA1960 were analyzed (**Supplementary Figure S2**). Nine polymorphisms representing three unique alleles were identified, of which seven SNPs specific for accession LA0130, one for LA1960 and one for LA2737. All these SNPs differ from the *Ty-1*/*Ty-3* alleles uncovered from previous studies (Verlaan et al., 2013; Caro et al., 2015). Further, the protein sequences obtained by *in silico* translating the amplified coding sequences were aligned (**Supplementary Figure S3**). Four allele-specific amino acids (aa) were observed to be unique and not present in other RDR alleles identified before. For *S. chilense* accession LA1960, the allele-specific aa is F22 (**Supplementary Figure S3**, yellow marked). LA0130-specific aa are V139, Q805, and K912 (**Supplementary Figure S3**, yellow marked). In addition, a previous study showed that TYLCV-resistant *S. chilense* species carry three *Ty-1*/*Ty-3* specific aa (Caro et al., 2015), which are present in the tested accessions (**Supplementary Figure S3**, red marked).

### Previously Published Data on Screening of Accessions With Resistance and Susceptibility to TYLCV

In Spain, numerous wild tomato species have been screened for resistance to TYLCD by researchers at the Institute for the Conservation and Improvement of Agrodiversity (COMAV). These screens utilized whitefly-mediated and Agroinoculation methods with either TYLCV or TYLCSV, and some accessions were tested with both viruses. TYLCSV is the first reported geminivirus in Spain. The screening work started in mid-1990s, when TYLCSV was the predominant TYLCV-related *Begomovirus* present in Spain (Moriones et al., 1993). In total, 143 accessions were screened for resistance, the results of which we summarize from nine publications (Jordá et al., 1996; Picó and Díez, 1998; Picó et al., 1998, 1999b, 2000; Soler et al., 2000; Pérez de Castro et al., 2004, 2005, 2010).

Among 143 accessions tested, highly and moderately resistant accessions were found in *S. chilense* (17 accessions), *S. lycopersicum* var. *cerasiforme* (1 accession), *S. habrochaites* (4 accessions), *S. lycopersicoides* (7 accessions), *S. neorickii* (1 accession), *S. peruvianum* (7 accessions), and *S. pimpinellifolium* (5 accessions) (**Supplementary Table S5**). Accessions that were resistant against TYLCSV were identified in *S. chilense* (LA1932 and LA1963), *S. habrochaites* (ECU336 and ECU436), *S. neorickii* (ECU301), *S. peruvianum* (ECU446, PI 126935, and PI 143679), *S. pimpinellifolium* (UPV16953) and *S. lycopersicum* var. *cerasiforme* (ECU464) (**Supplementary Table S5**). Later, TYLCV was introduced in Spain and was reported worldwide causing serious TYLCD epidemics, thus, this viral species was also included in some of the screenings. All the resistant accessions identified in *S. lycopersicoides* were effective against TYLCV. Considering the importance of both viral species, in many of the screenings, phenotypic responses to both TYLCV and TYLCSV were evaluated. These intensive screenings resulted in identification of many accessions being resistant to both TYLCV species from *S. chilense*, *S. habrochaites*, *S. peruvianum*, and *S. pimpinellifolium*, indicating a very strong correlation between resistance to TYLCV and resistance to TYLCSV (**Supplementary Table S5**). The highest levels of resistance were found among *S*. *chilense* and *S*. *peruvianum* accessions when challenged with two viral species in different inoculation assays. High variability was found among *S*. *pimpinellifolium* and *S*. *habrochaites* accessions. Intermediate level of resistance was found in *S*. *pimpinellifolium* accessions with mild to moderate viral symptoms (**Supplementary Table S5**). *S. pennellii* accessions included in the screenings were rated as susceptible (**Supplementary Table S5**; Picó et al., 2000; Soler et al., 2000).

In addition to the above mentioned large scale tests carried out at COMAV, we summarized resistant and susceptible accessions in previously published resources (**Supplementary Table S5**). Worldwide efforts on identification of promising resistance sources against TYLCV viral complex have resulted in various resistant accessions corresponding to *S. arcanum*, *S. cheesmaniae*, *S. chilense*, *S. chmielewskii*, *S. corneliomulleri*, *S. galapagense*, *S. habrochaites*, *S. neorickii*, *S. pennellii*, *S. peruvianum*, and *S. pimpinellifolium* (**Supplementary Table S5** with literature). Resistant accessions identified up till now from previous publications were mainly from wild species *S. chilense*, *S. habrochaites*, *S. peruvianum*, and *S. pimpinellifolium* (**Supplementary Table S5**). All the identified *S. chilense* accessions showed a high level of resistance exhibiting no viral symptoms. Among *S. peruvianum* and *S. habrochaites* accessions, both high levels of resistance (no symptoms) as well as mild to moderate levels of resistance were identified. Resistant accessions from *S. pimpinellifolium* mostly showed a moderate level of resistance which allows slight viral symptoms (**Supplementary Table S5**).

### Introgression Breeding for TYLCV Resistance From Diverse Tomato Genetic Pools

Currently, *Ty-1*, *Ty-2*, and *Ty-3* are the primary resistance genes widely used in tomato breeding programs reported in literature. The *Ty-4* resistance locus confers only a low level of resistance, while the *ty-5* gene is recessive in nature; therefore, the utilization of these genes in tomato breeding programs is restricted (Ji et al., 2009; Lapidot et al., 2015). *Ty-6* is an incompletely dominant resistance locus that was more recently identified (Hutton and Scott, 2014). Little is known about *Ty-6* or the extent to which it is utilized commercially. *Ty-1*, *Ty-3*, *Ty-4*, and (reportedly) *Ty-6* all originated from various *S. chilense* accessions, including LA1969, LA2779, LA1932, and LA1938 (Zamir et al., 1994; Ji et al., 2007a, 2009; Hutton and Scott, 2014). In some cases, a single *S. chilense* accession can harbor more than one TYLCV resistance locus (e.g., LA1932 contains both *Ty-1* and *Ty-4*; LA2779 contains both *Ty-3* and *Ty-*6) (Ji et al., 2007a, 2009; Hutton and Scott, 2014; Caro et al., 2015). Resistance in commercial breeding materials can likewise be mediated by a single resistance gene or a joint response of different genes.

At COMAV, promising resistance sources in a number of wild species, such as *S. pimpinellifolium*, *S. peruvianum*, and *S. chilense*, were used to develop advanced resistant generations. UPV16991 was the most resistant *S. pimpinellifolium* accession identified. Resistance in L102, a UPV16991-derived inbred line, was monogenic and incompletely dominant. The resistance was characterized by a restriction of viral particle accumulation (Pérez de Castro et al., 2007). Pyramiding resistance derived from UPV16991 and the *Ty-1* gene increased the level of resistance in different crosses between *Ty-1* lines (BC_7_S_1_ generation from the cross *S. lycopersicum* NE-1 and LA3473) and UPV16991-derived lines (Pérez de Castro et al., 2008). In *S. peruvianum*, PI 126944 was selected for resistance to TYLCV (Picó and Díez, 1998). Three interspecific hybrids were obtained between cultivated tomato NE-1 and PI 126944, and a set of introgression lines into the genetic background of tomato is being developed from these (Picó et al., 2002). In some of these generations the resistance level against TYLCV and TYLCSV has been assessed (Julián et al., 2013). Interspecific hybrids between cultivated tomato (*S. lycopersicum*) and *S. chilense* accessions (i.e., LA1932, LA1938, LA1959, LA1960, LA1963, LA1969, LA1971, LA2779, LA3473, UPV20306, UPV20328, and UPV20329) were made leading to different types of populations (Picó et al., 1999c, 2002; Pérez de Castro et al., 2013). In many *S*. *chilense* accessions including LA1932, LA1938, LA1960, and LA1971, TYLCV resistance loci have been mapped to tomato chromosome 6, in the *Ty-1*/*Ty-3* region (Caro et al., 2015). Interspecific hybrids were obtained between *S. lycopersicum* and *S. chilense* accessions UPV20306, UPV20328, and UPV20329. However, the level of resistance derived from these accessions seemed to be lower than resistance derived from the rest of the exploited sources (Pérez de Castro et al., 2005).

At the World Vegetable Center in Taiwan, breeding for resistance against TYLCD has focused intensely on *Ty-2*-mediated resistance, and *Ty-2* has been extensively exploited in tomato breeding worldwide. CLN2116 is an F_7_ determinate tomato line developed at AVRDC with *Ty-2* introgression (AVRDC, 2001). Three breeding lines namely CLN2513, CLN2514, and CLN2515 contain combined resistance derived from *Ty-1* and *Ty-2*. These lines have been generated by double crossing three tomato lines (CLN399, CH154, and CLN2026) with BL982 (*Ty-1*-based resistance) as well as CLN2116 (AVRDC, 2002). Multiple determinate tomato lines with TYLCD resistance have been developed including CLN2468A, CLN2468B, CLN2468C, CLN2468D, CLN2469C, CLN2460F, CLN2467E, CLN2467F, and CLN2467G. At the same time, indeterminate tomato lines including CLN2460G, CLN2460H, CLN2460I, CLN2460J, CLN2463O, and CLN2463P are available for breeding purposes. All these breeding lines possess *Ty-2* derived originally from H24 (AVRDC, 2003). TYLCD-resistant breeding line CLN2777A was derived from the cross between CL5915 and H24, a carrier of the *Ty-2* locus. There are also breeding lines available at AVRDC carrying various combinations of TYLCD resistance loci. CLN3150A-5 has excellent disease resistance, being homozygous for *Ty-2* and *ty-5* genes (AVRDC, 2016). There are various improved lines developed at the World Vegetable Center with TYLCD resistance mediated by *Ty-2* or in combination with other known TYLCD resistance genes (i.e., *Ty-1*/*Ty-3* and *ty-5*). CLN2498D^3^ and CLN3024A^3^ are both (semi-) determinate lines carrying TYLCD resistance gene, *Ty-2*. CLN3241H-27^3^ and CLN3241Q^3^ are proper candidates for open field cultivation with a high level of TYLCV resistance achieved by pyramiding *Ty-1*/*Ty-3* and *Ty-2* resistance genes. FMTT1733D^3^ and FMTT1733E^3^ are indeterminate lines harboring *Ty-2* and *Ty-3*. CLN3736D^3^ is a semi-determinate open field line with superior TYLCD resistance driven by the combined effort of *Ty-1*/*Ty-3*, *Ty-*2, and *ty-5*. Cultivars or improved breeding lines developed at the World Vegetable Center are available for breeding purposes or for local agronomic performance testing for future release.

*Begomovirus* resistance breeding efforts at the University of Florida, Gulf Coast Research and Education Center (GCREC) began in the early 1990s. Although initial breeding efforts involved a larger number of *S. chilense* accessions, resistance derived from *S. chilense* accessions LA1932, LA1938, LA2779 and from *S. lycopersicum* cv. Tyking later became the primary focus of the program. *Ty-1* and *Ty-3* are widely used for TYLCV resistance in many breeding programs worldwide. However, undesirable horticultural traits are generally coupled with both introgressions, known as linkage drag. Recent breeding efforts at GCREC have focused on reducing the size of *Ty-1* and *Ty-3* introgressions. Fla.8923 is a product of these efforts, and contains *Ty-3* within a 70 Kb *S. chilense* introgression (Hutton et al., 2015). Similarly, Fla.7907C and Fla.7781B each contain *Ty-1* within an approximately 1 Mb introgression (Hutton and Scott, 2017; Hutton, Unpublished). Fla.8624 is a breeding line containing *Ty-6* showing an intermediate level of resistance to TYLCV. Fla.8638B is a breeding line pyramiding *ty-5* and *Ty-6*, displaying a high resistance level against a wide range of *Begomoviruses* (Scott et al., 2015).

Besides the three major centers (COMAV, AVRDC, and GCREC) that have generated advanced breeding lines and released them for breeding purposes, there are other programs aiming at deploying resistant wild tomato species to breed for TYLCV resistance. Various advanced populations have been developed starting from *S. habrochaites* accessions. The majority of them were derived from accession B6013, which is the donor of the *Ty-2* resistance gene (Kalloo and Banerjee, 1990; Picó et al., 2002; Chomdej et al., 2007). Besides accession B6013, promising resistant lines were developed using *S. habrochaites* accessions LA1777 and LA0386 as well as EELM-889 (Vidavsky and Czosnek, 1998; Tomás et al., 2011). Combined resistance derived from both LA1777 and LA0386 has been introgressed into cultivated tomato. This resulted in segregating families displaying responses to TYLCV ranging from resistance and tolerance to susceptibility. A BC_1_S_4_ inbred line (named 902) that is fixed for TYLCV resistance was obtained (Vidavsky and Czosnek, 1998). *S. habrochaites* accession EELM-889 provides effective resistance against multiple TYLCD associated viruses (Tomás et al., 2011). Segregating populations (F_2_, F_3_, and BC_1_) were developed to further characterize EELM-889 mediated resistance against the Israel strain of TYLCV-IL. TYLCV-IL is the most widespread and economically important TYLCD causing *Begomovirus* species (Lefeuvre et al., 2010). The resistance to TYLCV-IL is controlled by two distinct loci, one dominant and another recessive (Tomás et al., 2011). In addition, resistant accessions from *S. peruvianum* were selected including PI 126935, PI 126926, PI 128648, and EC104395 to initiate the introgression of resistance into the cultivated tomato (Pilowsky and Cohen, 1990; Vidavsky et al., 1998; Maruthi et al., 2003). Resistance sources have also been uncovered from *S. pimpinellifolium* LA1921 and efforts have been taken to further obtain different populations (backcross, F_1_, and F_2_) (Banerjee and Kalloo, 1987). Results obtained by testing these populations for their response to TYLCV infection indicated that the resistance mediated by LA1921 was monogenic and incompletely dominant (Banerjee and Kalloo, 1987).

## Discussion

In this study, we presented our own and worldwide efforts so far on the identification of potential tomato wild accessions for resistance to TYLCD. Out of more than 700 accessions of 13 wild tomato species, about 140 accessions showed a symptomless response to TYLCD infection, either by agroinfiltration or by natural whitefly infection. Virus replication was detected in most symptomless accessions identified by agroinfiltration, showing that these examined accessions are not immune to TYLCV. Further, unique functional *Ty-1*/*Ty-3* alleles are present in *S. chilense* accessions LA0130, LA2737, and LA1960. Meanwhile, by summarizing to date the breeding efforts in breeding tomato with TYLCV resistance, we demonstrated that a very small number of resources has been used as resistance genitors in commercial cultivars. TYLCV has a great potential to change due to factors including virus recombination, mutations, additions of satellite and invasion of exogenous whitefly species (Nawaz-ul-Rehman and Fauquet, 2009; Czosnek and Ghanim, 2011; Hosseinzadeh et al., 2014). For example the TYLCV-IS76 strain, a recombinant between TYLCV-IL and the Spanish strain of TYLC-Sardinia virus (TYLCSV-ES), which can accumulate better than its parental strains in cultivars carrying the *Ty-1* gene (Belabess et al., 2015, 2016). It has been shown that *Ty-2* based resistance can be overcome by TYLCSV and the TYLCV-Mld strain which has a TYLCSV-like C4 protein (Barbieri et al., 2010; Tomás et al., 2011; Ohnishi et al., 2016). Therefore, the TYLCV symptomless accessions identified in this study represent a treasure of resources to tomato breeders.

Our focus of the current study was on the screening of germplasm collection for resistance to TYLCV and we defined resistance only as symptomless response to TYLCV. It should be noted that majority of the accessions was screened in the field using whitefly-mediated natural inoculation. For some accessions symptomless plants may have occurred due to escape or avoidance of whitefly infection. Therefore, the true TYLCV resistance should be determined by retesting these symptomless accessions with controlled inoculation approaches as well as by quantifying virus titers. Here, we discuss several issues in the context with follow-up studies and with the further use of these accessions for breeding purpose.

### The Avoidance/Resistance to Whitefly

Previous screening studies on *S. peruvianum* under whitefly inoculation reported high levels of resistance to TYLCV. The resistance was overcome under graft-inoculated conditions for the same accessions (Azizi et al., 2008), suggesting a possible resistance to whitefly rather than to TYLCV. Similar results were obtained in the present study for *S. habrochaites* accession LA2314, symptomless in whitefly-mediated screening and symptomatic in Agrobacterium-mediated inoculation (**Table 2**). These results indicate that symptomless response of *S. habrochaites* accession LA2314 is possibly associated with the resistance to whitefly, probably owning to the presence of glandular trichomes. In various wild tomato species, the presence of glandular trichomes on the leaf surface contributes to whitefly resistance by entrapping the whiteflies, and thereby possibly changing their feeding behavior (Momotaz et al., 2010; Rodríguez-López et al., 2011; Firdaus et al., 2012; Andrade et al., 2017; Rakha et al., 2017). In *Solanum* spp., type IV and VI trichomes contribute to a high level of resistance to whitefly, which is attributed to the exudates of glandular trichomes (Kennedy, 2003). Secondary metabolites released by tomato glandular trichomes influence the whiteflies’ preference for or avoidance of specific plants, and their feeding behavior (Bleeker et al., 2009, 2011; Rodríguez-López et al., 2011). Thus, accessions displaying natural resistance against the transmission vector may not be resistant to its transmittable viral species. Before using these symptomless accessions identified upon whitefly natural infection, it is worthwhile to test the selected accessions with the viral species under investigation using Agrobacterium-mediated inoculation.

### Resistance to TYLCV or to TYLCV-Like Viruses

In screenings worldwide, accessions were tested with the virus strain that was endemic to the area. This approach likely contributes to the identification of strain-specific resistant accessions that may benefit regional breeding programs. In the field, mixed infections frequently occur. As transmission vector, it is possible for whiteflies to transmit different types of virus as well as various TYLCV-like viral species (Díaz-Pendón et al., 2010). While, in controlled artificial inoculation the viral strain is known. For example, *S. lycopersiciodes* accession CGN23973 showed a high level of resistance against TYLCV-IL strain when using Agrobacterium-mediated infection. However, it was symptomatic in the field with natural whitefly infection. We did not determine the virus strains present in the field in China. Possibly, CGN23973 may not be resistant to other TYLCV(-like) strains that whitefly transmit in the open field. In addition, plants are continuously exposed to large whitefly populations in the field. TYLCV incidence and severity may be influenced by the level of disease pressure. For example, the *Ty-1* conferred resistance was broken under high inoculum pressure (García-Cano et al., 2008). Therefore, in the case of *S. lycopersiciodes* CGN23973, an alternative possible explanation for the contrasting phenotypic responses can be the high disease pressure with whitefly natural infection.

### Alleles of Known TYLCV Resistance Loci

Almost all of the screened accessions of *S. chilense* did not support any viral symptom development which makes *S. chilense* the most promising resistance source. In multiple *S. chilense* accessions including LA1932, LA1938, and LA1971 allelic variants of the *Ty-1* gene contribute to the resistance (Caro et al., 2015). Accessions LA1932 and LA1938 also carry other genetic factors (i.e., *Ty-4* and *Ty-6*, respectively) (Ji et al., 2009; Hutton and Scott, 2014). We showed the resistance in three additional *S. chilense* accessions, LA0130, LA1960, and LA2737, is based on three distinct functional *Ty-1*/*Ty-3* alleles. In the VIGS experiment, the resistance in accessions LA1961, LA2981, and LA2754 was not compromised after silencing the *Ty-1* gene, which may be explained by two reasons. One is that other *Ty*-genes, such as *Ty-4* and *Ty-6* may be present in these accessions. The other is that *Ty-1* was not silenced due to the insufficient effect of VIGS. In our previous studies, we have identified three aa which are specific to *S. chilense* *Ty-1*/*Ty-3* alleles; as well as two aa that distinguish the *Ty-1* allele in LA1969 from *Ty-3* in LA2779 (Caro et al., 2015). In this study, three unique functional *Ty-1/Ty-3* alleles were discovered for *S. chilense* LA1932, LA1938, and LA1971. Based on these results, *Ty-1* and *Ty-3* allele-specific markers can be further developed as in-gene markers for more precise screenings in different breeding programs. Further, as the *Ty-2* and *ty-5* genes are also cloned (Lapidot et al., 2015; Yamaguchi et al., 2018), gene specific SNPs can be used to check the presence of these two genes in the resistant accessions of *S. habrochaites* (e.g., LA1777 and LA1718) and *S. peruvianum* (e.g., CGN15530, CGN15532, and PI 266375). These accessions, together with the other four symptomless accessions (*S. arcanum* LA2172, *S. huaylasense* LA1364 and LA1365, *S. neorickii* LA0735/CGN24193, **Table 1** and **Supplementary Table S2**), belong to the 150 Tomato Genome Re-sequenced genomes (Aflitos et al., 2014), which facilates the allele mining of the *Ty-2* and *ty-5* genes in these accessions.

### Introgression Breeding

Introgression of resistance from wild tomato species into cultivated tomatoes is the common practice for TYLCV resistance breeding. *S. chilense* and *S. peruvianum* are the most common sources of resistance commercially used among the wild tomato species (Pérez de Castro et al., 2007). *S. chilense* accessions screened in this study showed very high resistance levels. Many, if not most, tested accessions remained vigorous and completely symptomless during the whole screening period (**Figure 1**). This makes *S. chilense* the first priority to search for potential novel resistance sources in the diverse tomato gene pool. Besides *S. chilense*, a large number of symptomless accessions have also been identified among the screened accessions of *S. arcanum*, *S. corneliomulleri*, and *S. peruvianum*. These species may serve as the secondary gene pool for TYLCV resistance. However, these species belong to the “peruvianum” complex, a group of species having crossing barriers with *S. lycopersicum* (Díez and Nuez, 2008). A new wild tomato species segregated from *S. peruvianum* is described and classified as *S. huaylasense* (Peralta et al., 2005). Only four accessions of *S. huaylasense* were screened for their responses upon TYLCV infection, and all these accessions were resistant with no TYLCD symptoms. Thus *S. huaylasense* may serve as another potential gene pool for TYLCV resistance donors. In addition to crossing barriers, introgression can be hindered by chromosomal inversions between the wild resistant donor and the cultivated tomato such as the inversion present in *S. chilense* (the donor species of *Ty-1*) (Verlaan et al., 2011) and *S. habrochaites* (the donor species of *Ty-2*) (Wolters et al., 2015). Chromosomal rearrangements lead to suppression of recombination, resulting in linkage drag. In the case of *Ty-1*, traits with negative effects on several agronomic traits have been introduced along with *Ty-1* due to linkage drag (Rubio et al., 2010).

*S. pimpinellifolium* belongs to the “esculentum” complex that is completely crossable with *S. cheesmaniae*/*S. galapagense* and *S. lycopersicum*, the cultivated tomato (Díez and Nuez, 2008). This makes this species very suitable for breeding programs. Therefore, in this study, great efforts have been made on the screening of 408 *S. pimpinellifolium* accessions for sources of resistance. However, the majority of the tested accessions displayed severe levels of susceptibility. Only one *S. pimpinellifolium* accession G1.1554 (CGN15528) was free of TYLCD symptoms (**Table 1**). Several accessions showed a reduced level of susceptibility exhibiting mild to moderate TYLCD symptoms (**Table 1** and **Supplementary Table S2**). In other studies, few accessions (Hirsute-INRA and LA1478) from *S. pimpinellifolium* were identified that show resistance to TYLCV with no viral symptoms (Kasrani, 1989). G1.1554 (CGN15528) is currently being exploited for TYLCV resistance (Víquez-Zamora et al., 2014). Two quantitative trait loci (QTLs) contributing to TYLCV resistance were identified on chromosomes 3 and 11 (Víquez-Zamora et al., 2014).

In summary, this is the first time that such a large scale screening has been performed to uncover TYLCV resistance (symptomless) and susceptibility (symptomatic) in wild tomato germplasm. The majority of the symptomless accessions identified in this study have never been reported before in publications. Therefore, these symptomless accessions can be considered as novel sources of TYLCV resistance. Moreover, the other merit of this study is the summary of previous efforts on screening and using wild tomato accessions in breeding for TYLCV resistance. The latter makes it clear that a very small number of TYLCV resistance sources is presently used. Finally, with few *S. chilense* accessions, we demonstrate how allelic variants of a cloned *Ty*-gene can be discovered by VIGS in combination with allele mining. Therefore, this work constitutes a treasure of knowledge for the breeder who is urged to extend the very limited germplasm used to date as a donor for TYLCV resistance in commercial cultivars.

## Author Contributions

ZY, JL, A-MAW, and YB have conceived and designed the experiments. ZY, AP-d-C, MJD, and SFH have performed the experiments and analyzed the data. ZY, AP-d-C, MJD, SFH, RGFV, A-MAW, and YB have written and edited the paper.

## Conflict of Interest Statement

The authors declare that the research was conducted in the absence of any commercial or financial relationships that could be construed as a potential conflict of interest.

## Footnotes

1 https://www.cabi.org/isc/datasheet/8927

2 https://www.cabi.org/isc/datasheet/55402

3 http://avrdc.org/seed/improved-lines/fresh-market-tomato/

## Supplementary Material

FIGURE S1 Phenotypic responses of different wild *Solanum* species upon inoculation with tomato yellow leaf curl disease. Representatives of different tomato wild species are shown displaying variations in their symptom severity. From *left to right* are accessions corresponding to different *Solanum* species: *S. habrochaites* CGN15391, *S. pennellii* LA1809, *S. lycopersicoides* CGN23973, *S. chmielewskii* CGN15815, *S. cheesmaniae* LA1409, *S. neorickii* CGN15816, *S. peruvianum* CGN15795, *S. corneliomulleri* LA1944, and *S. lycopersicum* cv. MM. For each accession, two different individuals are shown.

FIGURE S2 Alignment of full-length cDNA sequences of the *Ty-1*/*Ty-3* allele in *S. chilense* accessions LA0130, LA2737, and LA1960. Sequences of *S. lycopersicum* “Moneymaker” (MM), the *Ty-1* allele from *S. chilense* LA1969 and the *Ty-3* allele from *S. chilense* LA2779 were described by Verlaan et al. (2013). *Ty-1*/*Ty-3* alleles in *S. chilense* LA1932, LA1938, and LA1971 were obtained from Caro et al. (2015).

FIGURE S3 Alignment of protein sequences of the *Ty-1*/*Ty-3* alleles. Protein sequences are derived from cDNA sequences in **Supplementary Figure S2**. Unique amino acids present in either LA1960 or LA0130 are highlighted in *yellow*. The three amino acids specific for the *Ty-1*/*Ty-3* allele are marked in *red*.

TABLE S1 Summary of responses of wild tomato species upon natural infection with tomato yellow leaf curl disease at the Institute of Vegetables and Flowers, Chinese Academy of Agricultural Sciences, Beijing.

TABLE S2 Average disease severity index ratings of *Solanum* spp. accessions upon natural infection with tomato yellow leaf curl disease at the Institute of Vegetables and Flowers, Chinese Academy of Agricultural Sciences, Beijing.

TABLE S3 Wild tomato accessions displaying variation in phenotypic responses upon natural infection with tomato yellow leaf curl disease at the Institute of Vegetables and Flowers, Chinese Academy of Agricultural Sciences, Beijing.

TABLE S4 Polymerase chain reaction detection of tomato yellow leaf curl virus (TYLCV) in wild accessions screened with Agrobacterium-mediated inoculation. Analyses were performed at 55 days post inoculation in young tissues of three tested individuals of each accession. Plant response was evaluated according to the intensity of viral DNA amplification and symptom expression.

TABLE S5 Previous published data on resistant/tolerant and susceptible wild tomato accessions against tomato yellow leaf curl virus complex.
