# Supplementary figures and images for: Resistance to Tomato Yellow Leaf Curl Virus in Tomato Germplasm
Source: Front Plant Sci. 2018 Aug 20;9:1198. doi: 10.3389/fpls.2018.01198 (PMC6110163; doi:10.3389/fpls.2018.01198)

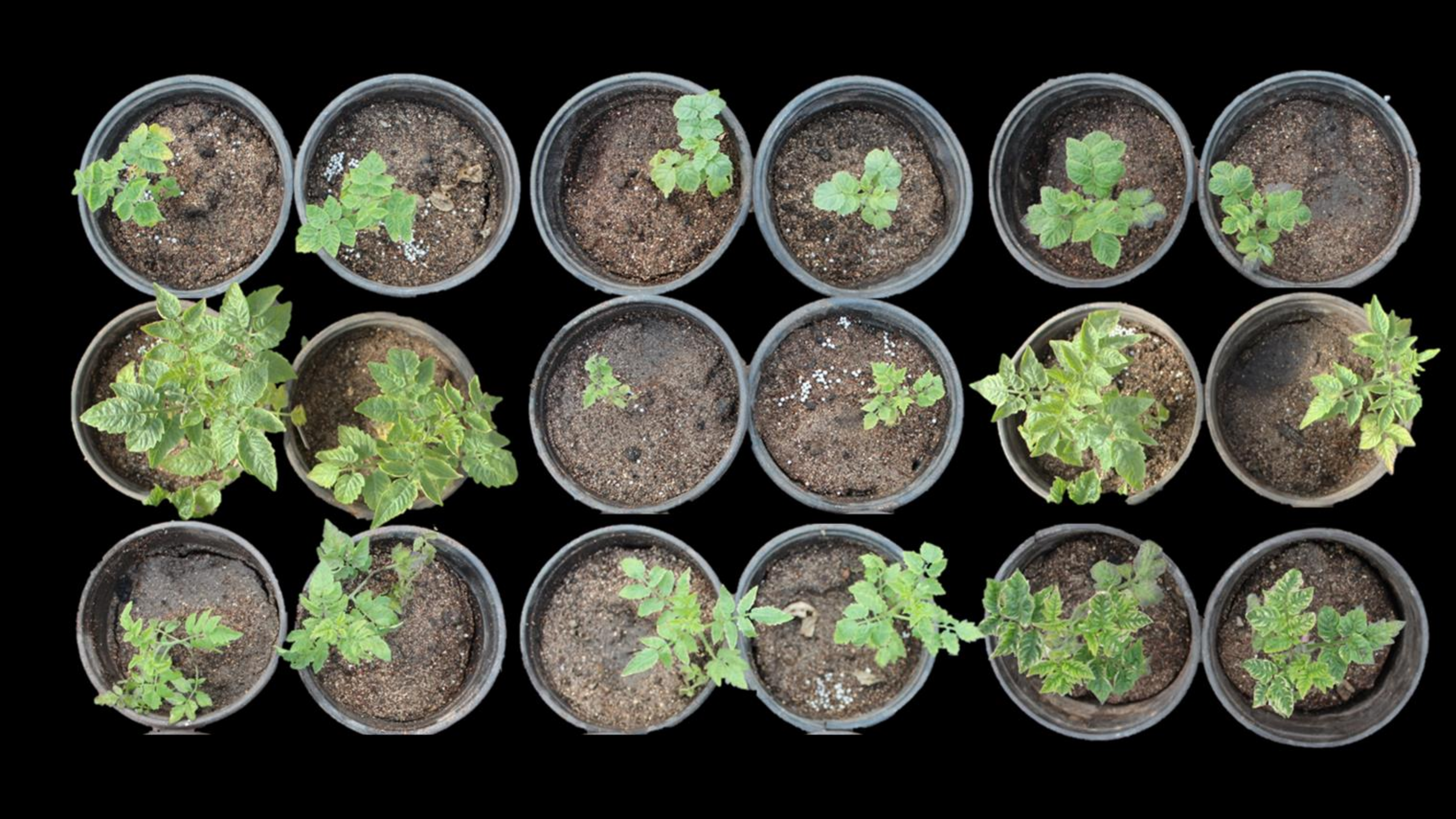

Supplement: FIGURE S1 — Phenotypic responses of different wild Solanum species upon inoculation with tomato yellow leaf curl disease. Representatives of different tomato wild species are shown displaying variations in their symptom severity. From left to right are accessions corresponding to different Solanum species: S. habrochaites CGN15391, S. pennellii LA1809, S. lycopersicoides CGN23973, S. chmielewskii CGN15815, S. cheesmaniae LA1409, S. neorickii CGN15816, S. peruvianum CGN15795, S. corneliomulleri LA1944, and S. lycopersicum cv. MM. For each accession, two different individuals are shown. [file Image_1.TIF]
