# Supplementary material for: Resistance to Tomato Yellow Leaf Curl Virus in Tomato Germplasm
Source: Front Plant Sci. 2018 Aug 20;9:1198. doi: 10.3389/fpls.2018.01198 (PMC6110163; doi:10.3389/fpls.2018.01198)
Supplement: FIGURE S2 — Alignment of full-length cDNA sequences of the Ty-1/Ty-3 allele in S. chilense accessions LA0130, LA2737, and LA1960. Sequences of S. lycopersicum “Moneymaker” (MM), the Ty-1 allele from S. chilense LA1969 and the Ty-3 allele from S. chilense LA2779 were described by Verlaan et al. (2013). Ty-1/Ty-3 alleles in S. chilense LA1932, LA1938, and LA1971 were obtained from Caro et al. (2015). [file Image_2.pdf]

```
                      exon 1
Slyc_MM_RDR        ATGGGTGATCCGTTGATTGAAGAAATTGATGTTC------------TGGATGCACCTTTACCATATTCTGTAGAGACGAT  68
Schil_LA1969_Ty1   ATGGGTGATCCGTTGATTGAAGAAATTGATGTTCCTTCTTGTATACTGGATGCACCTTTACCATATTCTGTAGAGACGAT  80
Schil_LA2779_Ty3   ATGGGTGATCCGTTGATTGAAGAAATTGATGTTCCTTCTTGTATACTGGATGCACCTTTACCATATTCTGTAGAGACGAT  80
Schil_LA1932_RDR   ATGGGTGATCCGTTGATTGAAGAAATTGATGTTCCTTCGTGTATACTGGATGCACCTTTACCATATTCTGTAGAGACGAT  80
Schil_LA1938_RDR   ATGGGTGATCCGTTGATTGAAGAAATTGATGTTCCTTCTTGTATACTGGATGCACCTTTACCATATTCTGTAGAGACGAT  80
Schil_LA1971_RDR   ATGGGTGATCCGTTGATTGAAGAAATTGATGTTCCTTCGTGTATACTGGATGCACCTTTACCATATTCTGTAGAGACGAT  80
Schil_LA0130_RDR   ATGGGTGATCCGTTGATTGAAGAAATTGATGTTCCTTCTTGTATACTGGATGCACCTTTACCATATTCTGTAGAGACGAT  80
Schil_LA1960_RDR   ATGGGTGATCCGTTGATTGAAGAAATTGATGTTCCTTCTTGTATACTGGATGCACCTTTACCATTTTCTGTAGAGACGAT  80
Schil_LA2737_RDR   ATGGGTGATCCGTTGATTGAAGAAATTGATGTTCCTTCTTGTATACTGGATGCACCTTTACCATATTCTGTAGAGACGAT  80

Slyc_MM_RDR        GCTTGATAGAATCTGCAAGGAGCAGGGGCAAAAACCACCGTGTACTGGCATTAGAAGGAGGCTGAGCTCTATTGGTGAAA  148
Schil_LA1969_Ty1   GCTTGATAGAATCTGCAAGGAGCAGGGGCAAAAACCACCGTGTACTGGCATTAGAAGGAGGCTGAGCTCTATTGGTGAAA  160
Schil_LA2779_Ty3   GCTTGATAGAATCTGCAAGGAGCAGGGGCAAAAACCACCGTGTACTCGCATTAGAAGGAGGCTGAGCTCTATTGGTGAAA  160
Schil_LA1932_RDR   GCTTGATAGAATCTGCAAGGAGCAGGGGCAAAAACCACCGTGTACTGGCATTAGAAGGAGGCTGAGCTCTATTGGTGAAA  160
Schil_LA1938_RDR   GCTTGATAGAATCTGCAAGGAGCAGGGGCAAAAACCACCGTGTACTGGCATTAGAAGGAGGCTGAGCTCTATTGGTGAAA  160
Schil_LA1971_RDR   GCTTGATAGAATCTGCAAGGAGCAGGGGCAAAAACCACCGTGTACTGGCATTAGAAGGAGGCTGAGCTCTATTGGTGAAA  160
Schil_LA0130_RDR   GCTTGATAGAATCTGCAAGGAGCAGGGGCAAAAACCACCGTGTACTGGCATTAGAAGGAGGCTAAGCTCTATTGGTGAAA  160
Schil_LA1960_RDR   GCTTGATAGAATCTGCAAGGAGCAGGGGCAAAAACCACCGTGTACTGGCATTAGAAGGAGGCTGAGCTCTATTGGTGAAA  160
Schil_LA2737_RDR   GCTTGATAGAATCTGCAAGGAGCAGGGGCAAAAACCACCGTGTACTGGCATTAGAAGGAGGCTGAGCTCTATTGGTGAAA  160

Slyc_MM_RDR        AAGGGTCATTAGAAATGCTCAAAATAATATCACGTCGTCCTATCAAGAAGAGTCTCTCTGCTTTTCTTGTTTACATGATT  228
Schil_LA1969_Ty1   AAGGGTCATTAGAAATGCTCAAAATAATATCACGTCGTCCTATCAAGAAGAGTCTCTCTGCTTTTCTTGTTTACATGATT  240
Schil_LA2779_Ty3   AAGGGTCATTAGAAATGCTCAAAATAATATCACGTCGTCCTATCAAGAAGAGTCTCTCTGCTTTTCTTGTTTACATGATC  240
Schil_LA1932_RDR   AAGGGTCATTAGAAATGCTCAAAATAATATCACGTCGTCCTATCAAGAAGAGTCTCTCTGCTTTTCTTGTTTACATGATC  240
Schil_LA1938_RDR   AAGGGTCATTAGAAATGCTCAAAATAATATCACGTCGTCCTATCAAGAAGAGTCTCTCTGCTTTTCTTGTTTACATGATT  240
Schil_LA1971_RDR   AAGGGTCATTAGAAATGCTCAAAATAATATCACGTCGTCCTATCAAGAAGAGTCTCTCTGCTTTTCTTGTTTACATGATC  240
Schil_LA0130_RDR   AAGGGTCATTAGAAATGCTCAAAATAATATCACGTCGTCCTATCAAGAAGAGTCTCTCTGCTTTTCTTGTTTACATGATC  240
Schil_LA1960_RDR   AAGGGTCATTAGAAATGCTCAAAATAATATCACGTCGTCCTATCAAGAAGAGTCTCTCTGCTTTTCTTGTTTACATGATT  240
Schil_LA2737_RDR   AAGGGTCATTAGAAATGCTCAAAATAATATCACGTCGTCCTATCAAGAAGAGTCTCTCTGCTTTTCTTGTTTACATGATT  240
```

```
Slyc_MM_RDR        GATCGCTACCCGGATTGTCTCTCCTCTTCCTCTAGCCCCTTCAATTGTCTACTCAAACGCTCTTCTTCCCCTCGTCTCTT 308
Schil_LA1969_Ty1   GATCGCTACCCGGATTGTCTCTCCTCTTCCTCTAGCCCATTCAATAGTCTACTCAAACGCTCTTCTTCCCCTGTTCTATT 320
Schil_LA2779_Ty3   GATCGCTACCCGGATTGTCTCTCCTCTTCCTCTAGCCCCTTCAATAGTCTACTCAAACGCTCTTCTTCCCCTCGTCTCTT 320
Schil_LA1932_RDR   GATCGCTACCCGGATTGTCTCTCCTCTTCCTCTAGCCCATCCAATAGTCTACTCAAACGCTCTTCTTCCCCTCTTCTCTT 320
Schil_LA1938_RDR   GATCGCTACCCGGATTGTCTCTCCTCTTCCTCTAGCCCCTTCAATAGTCTACTCAAACGCTCTTCTTCCCCTGTTCTATT 320
Schil_LA1971_RDR   GATCGCTACCCGGATTGTCTCTCCTCTTCCTCTAGCCCATCCAATAGTCTACTCAAACGCTCTTCTTCCCCTGTTCTCTT 320
Schil_LA0130_RDR   GATCGCTACCCGGATTGTCTCTCCTCTTCCTCTAGCCCATCCAATAGTCTACTCAAACGCTCTTCTTCCCCTGTTCTCTT 320
Schil_LA1960_RDR   GATCGCTACCCGGATTGTCTCTCCTCTTCCTCTAGCCCATTCAATAGTCTACTCAAACGCTCTTCTTCCCCTGTTCTATT 320
Schil_LA2737_RDR   GATCGCTACCCGGATTGTCTCTCCTCTTCCTCTAGCCCCTTCAATAGTCTACTCAAACGCTCTTCTTCCCCTGTTCTATT 320

                              exon 2
Slyc_MM_RDR        TCCATCTCCAGAGGGTAAACGTTTACAAGGTGAAAGTTCTTCTAAATCAAAGCTTGAGATGGGCTTATTGGCCTGTGCAA 388
Schil_LA1969_Ty1   TCCATCTCCAGAGGGTAAACGTTTACAAGGTGAAAGTTCTTCTAAATCAAAGCTTGAGATGGGCTTATTGGCCTGTGCAA 400
Schil_LA2779_Ty3   TCCATCTCCAGAGGGTAAACGTTTACAAGGTGAAAGTTCTTCTAAATCAAAGCTTGAGATGGGCTTATTGGCCTGTGCAA 400
Schil_LA1932_RDR   TCCATCTCCAGAGGGTAAACGTTTACAAGGTGAAAGTTCTTCTAAATCAAAGCTTGAGATGGGCTTATTGGCCTGTGCAA 400
Schil_LA1938_RDR   TCCATCTCCAGAGGGTAAACGTTTACAAGGTGAAAGTTCTTCTAAATCAAAGCTTGAGATGGGCTTATTGGCCTGTGCAA 400
Schil_LA1971_RDR   TCCATCTCCAGAGGGTAAACGTTTACAAGGTGAAAGTTCTTCTAAATCAAAGCTTGAGATGGGCTTATTGGCCTGTGCAA 400
Schil_LA0130_RDR   TCCATCTCCAGAGGGTAAACGTTTACAAGGTGAAAGTTCTTCTAAATCAAAGCTTGAGATGGGCTTATTGGCCTGTGCAA 400
Schil_LA1960_RDR   TCCATCTCCAGAGGGTAAACGTTTACAAGGTGAAAGTTCTTCTAAATCAAAGCTTGAGATGGGCTTATTGGCCTGTGCAA 400
Schil_LA2737_RDR   TCCATCTCCAGAGGGTAAACGTTTACAAGGTGAAAGTTCTTCTAAATCAAAGCTTGAGATGGGCTTATTGGCCTGTGCAA 400

Slyc_MM_RDR        GCCCTCAGAAAGTTGCTCGCCAGTTATCATTTTGCGAGGAGCCTGAATCTAACTGTAGAAGAACCTCCCCTTATGTCAGC 468
Schil_LA1969_Ty1   GCCCTCAGAAAGTTGCTCGCCAGTTATCATTTTGCGAGGAGCCTGAATCTAACTGTAGAAGAACCTCCCCTTATGTCAGC 480
Schil_LA2779_Ty3   GCCCTCAGAAAGTTGCTCGCCAGTTATCATTTTGCGAGGAGCCTGAATCTAACTGTAGAAGAACCTCCCCTTATGTCAGC 480
Schil_LA1932_RDR   GCCCTCAGAAAGTTGCTCGCCAGTTATCATTTTGCGAGGAGCCTGAATCTAACTGTAGAAGAACCTCCCCTTATGTCAGC 480
Schil_LA1938_RDR   GCCCTCAGAAAGTTGCTCGCCAGTTATCATTTTGCGAGGAGCCTGAATCTAACTGTAGAAGAACCTCCCCTTATGTCAGC 480
Schil_LA1971_RDR   GCCCTCAGAAAGTTGCTCGCCAGTTATCATTTTGCGAGGACCCTGAACCTAACTGTAGAAGAACCTCCCCTTATGTCAGC 480
Schil_LA0130_RDR   GCCCTCAGAAAGTTGTTCGCCAGTTATCATTTTGCGAGGAGCCTGAATCTAACTGTAGAAGAACCTCCCCTTATGTCAGC 480
Schil_LA1960_RDR   GCCCTCAGAAAGTTGCTCGCCAGTTATCATTTTGCGAGGACCCTGAACCTAACTGTAGAAGAACCTCCCCTTATGTCAGC 480
Schil_LA2737_RDR   GCCCTCAGAAAGTTGCTCGCCAGTTATCATTTTGCGAGGAGCCTGAATCTAACTGTAGAAGAACCTCCCCTTATGTCAGC 480
```

```
                                                                                     exon 3
Slyc_MM_RDR         CAACAGTTGATGATCCTCAATGAACTTGAATTTAGAAAATTGTTTCTGGTACTGAGCTACATTGGATGCAACAAGTTGGA 548
Schil_LA1969_Ty1    CAACAGTTGATGATCCTCAATGAACTTGAATTTAGAAAATTGTTTTTGGTACTGAGCTACATTGGATGCAACAAGTTGGA 560
Schil_LA2779_Ty3    CAACAGTTGATGATCCTCAATGAACTTGAATTTAGAAAATTGTTTTTGGTACTGAGCTACATTGGATGCAACAAGTTGGA 560
Schil_LA1932_RDR    CAACAGTTGATGATCCTCAATGAACTTGAATTTAGAAAATTGTTTTTGGTACTGAGCTACATTGGATGCAACAAGTTGGA 560
Schil_LA1938_RDR    CAACAGTTGATGATCCTCAATGAACTTGAATTTAGAAAATTGTTTTTGGTACTGAGCTACATTGGATGCAACAAGTTGGA 560
Schil_LA1971_RDR    CAACAGTTGATGATCCTCAATGAACTTGAATTTAGAAAATTGTTTTTGGTACTGAGCTACATTGGATGCAACAAGTTGGA 560
Schil_LA0130_RDR    CAACAGTTGATGATCCTCAATGAACTTGAATTTAGAAAATTGTTTTTGGTACTGAGCTACATTGGATGCAACAAGTTGGA 560
Schil_LA1960_RDR    CAACAGTTGATGATCCTCAATGAACTTGAATTTAGAAAATTGTTTTTGGTACTGAGCTACATTGGATGCAACAAGTTGGA 560
Schil_LA2737_RDR    CAACAGTTGATGATCCTCAATGAACTTGAATTTAGAAAATTGTTTTTGGTACTGAGCTACATTGGATGCAACAAGTTGGA 560

Slyc_MM_RDR         AGATGTTATATCCCCTCAAATTGCTGATGATATTGTAAGAAAGAAAAATCTTTCCATGACTGATTTTGAATCAGAAATTT 628
Schil_LA1969_Ty1    AGATGTTATATCCCCTCAAATTGCTGATGATATTGTAAGAAAGAAAGATCTTTCCATGACTGATTTTGAATCAGAAATTT 640
Schil_LA2779_Ty3    AGATGTTATATCCCCTCAAATTGCTGATGATATTGTAAGAAAGAAAGATCTTTCCATGACTGATTTTGAATCAGAAATTT 640
Schil_LA1932_RDR    AGAAGTTATATCCCCTCAAATTGCTGATGATATTGTAAGAAAGAAAAATCTTTCCATGACTGATTTTGAATCAGAAATTT 640
Schil_LA1938_RDR    AGATGTTATATCCCCTCAAATTGCTGATGATATTGTAAGAAAGAAAGATCTTTCCATGACTGATTTTGAATCAGAAATTT 640
Schil_LA1971_RDR    AGATGTTATATCCCCTCAAATTGCTGATGATATTGTAAGAAAGAAAGATCTTTCCATGACTGATTTTGAATCAGAAATTT 640
Schil_LA0130_RDR    AGATGTTATATCCCCTCAAATTGCTGATGATATTGTAAGAAAGAAAGATCTTTCCATGACTGATTTTGAATCAGAAATTT 640
Schil_LA1960_RDR    AGATGTTATATCCCCTCAAATTGCTGATGATATTGTAAGAAAGAAAGATCTTTCCATGACTGATTTTGAATCAGAAATTT 640
Schil_LA2737_RDR    AGATGTTATATCCCCTCAAATTGCTGATGATATTGTAAGAAAGAAAGATCTTTCCATGACTGATTTTGAATCAGAAATTT 640

                                                                 exon 4
Slyc_MM_RDR         GGAATGCTTTTGGAAAAGCATGTTATGCTGTGTCAGATAGATCAAAGTACTTAGACTGGAATTGCAGAAAGACACATATC 708
Schil_LA1969_Ty1    GGAATGCTTTTGGAAAAGCATGTTATGCTGTGTCAGATAGATCAAAGTACTTAGACTGGAATTGCAGAAAGACACATATC 720
Schil_LA2779_Ty3    GGAATGCTTTTGGAAAAGCATGTTATGCTGTGTCAGATAGATCAAAGTACTTAGACTGGAATTGCAGAAAGACACATATC 720
Schil_LA1932_RDR    GGAATGCTTTTGGAAAAGCATGTTATGCTGTGTCAGATAGATCAAAGTACTTAGACTGGAATTGCAGAAAGACACATATC 720
Schil_LA1938_RDR    GGAATGCTTTTGGAAAAGCATGTTATGCTGTGTCAGATAGATCAAAGTACTTAGACTGGAATTGCAGAAAGACACATATC 720
Schil_LA1971_RDR    GGAATGCTTTTGGAAAAGCATGTTATGCTGTGTCAGATAGATCAAAGTACTTAGACTGGAATTGCAGAAAGACACATATC 720
Schil_LA0130_RDR    GGAATGCTTTTGGAAAAGCATGTTATGCTGTGTCAGATAGATCAAAGTACTTAGACTGGAATTGCAGAAAGACACATATC 720
Schil_LA1960_RDR    GGAATGCTTTTGGAAAAGCATGTTATGCTGTGTCAGATAGATCAAAGTACTTAGACTGGAATTGCAGAAAGACACATATC 720
Schil_LA2737_RDR    GGAATGCTTTTGGAAAAGCATGTTATGCTGTGTCAGATAGATCAAAGTACTTAGACTGGAATTGCAGAAAGACACATATC 720
```

```
                                                       exon 5
Slyc_MM_RDR          TACTATTGCCACATTAAGCAGAACGGATACTGTTCCTTCAAGGGTCCATACTTGAACACATTAAGGACTCACTTACAGAG  788
Schil_LA1969_Ty1     TACTATTGCCACATTAAGCAGAACGGATGCTGTACCTTCAAGGGTCCATACTTGAACACAGCAAGGACTCACTTACAGAG  800
Schil_LA2779_Ty3     TACTATTGCCACATTAAGCAGAACGGATGCTGTACCTTCAAGGGTCCATACTTGAACACAGCAAGGACTCACTTACAGAG  800
Schil_LA1932_RDR     TACTATTGCCACATTAAGCAGAACGGATGCTGTACCTTCAAGGGTCCATACTTGAACACAGCAAGGACTCACTTACAGAG  800
Schil_LA1938_RDR     TACTATTGCCACATTAAGCAGAACGGATGCTGTACCTTCAAGGGTCCATACTTGAACACAGCAAGGACTCACTTACAGAG  800
Schil_LA1971_RDR     TACTATTGCCACATTAAGCAGAACGGATGCTGTACCTTCAAGGGTCCATACTTGAACACAGCAAGGACTCACTTACAGAG  800
Schil_LA0130_RDR     TACTATTGCCACATTAAGCAGAACGGATGCTGTACCTTCAAGGGTCCATACTTGAACACAGCAAGGACTCACTTACAGAG  800
Schil_LA1960_RDR     TACTATTGCCACATTAAGCAGAACGGATGCTGTACCTTCAAGGGTCCATACTTGAACACAGCAAGGACTCACTTACAGAG  800
Schil_LA2737_RDR     TACTATTGCCACATTAAGCAGAACGGATGCTGTACCTTCAAGGGTCCATACTTGAACACAGCAAGGACTCACTTACAGAG  800

Slyc_MM_RDR          AGCCCTGGGAGATGACAATGTACTGATTGTAAAATTTGTTGAAGATACAAGTTGTGCCAATATAATTCTCGAGGAAGGCA  868
Schil_LA1969_Ty1     AGCCCTGGGAGATGACAATGTACTGATTGTCAAATTTGTTGAAGATACAAGTTGTGCCAATATAATTCTCGAGGAAGGCA  880
Schil_LA2779_Ty3     AGCCCTGGGAGATGACAATGTACTGATTGTAAAATTTGTTGAAGATACAAGTTGTGCCAATATAATTCTTGAGGAAGGCA  880
Schil_LA1932_RDR     AGCCCTGGGAGATGACAATGTACTGATTGTAAAATTTGTTGAAGATACAAGTTGTGCCAATATAATTCTTGAGGAAGGCA  880
Schil_LA1938_RDR     AGCCCTGGGAGATGACAATGTACTGATTGTCAAATTTGTTGAAGATACAAGTTGTGCCAATATAATTCTCGAGGAAGGCA  880
Schil_LA1971_RDR     AGCCCTGGGAGATGACAATGTACTGATTGTCAAATTTGTTGAAGATACAAGTTGTGCCAATATAATTCTCGAGGAAGGCA  880
Schil_LA0130_RDR     AGCCCTGGGAGATGACAATGTACTGATTGTCAAATTTGTTGAAGATACAAGTTGTGCCAATATAATTCTCGAGGAAGGCA  880
Schil_LA1960_RDR     AGCCCTGGGAGATGACAATGTACTGATTGTCAAATTTGTTGAAGATACAAGTTGTGCCAATATAATTCTCGAGGAAGGCA  880
Schil_LA2737_RDR     AGCCCTGGGAGATGACAATGTACTGATTGTCAAATTTGTTGAAGATACAAGTTGTGCCAATATAATTCTCGAGGAAGGCA  880

                                               exon 6
Slyc_MM_RDR          TTCTTGTTGGCTTGAGACGTTACCGTTTCTTTGTGTATAAAGATGATAAAGAGAGGAAGAAAGTCCAGCTATGATGAAG  948
Schil_LA1969_Ty1     TTCTTGTTGGCTTGAGACGTTACCGTTTCTTTGTGTATAAAGATGATAAAGAGAGGAAGAAAGTCCAGCTATGATGAAG  960
Schil_LA2779_Ty3     TTCTTGTTGGCTTGAGACGTTACCGTTTCTTTGTGTATAAAGATGATAAAGAGAGGAAGAAAGTCCAGCTATGATGAAG  960
Schil_LA1932_RDR     TTCTTGTTGGCTTGAGACGTTACCGTTTCTTTGTGTATAAAGATGATAAAGAGAGGAAGAAAGTCCAGCTATGATGAAG  960
Schil_LA1938_RDR     TTCTTGTTGGCTTGAGACGTTACCGTTTCTTTGTGTATAAAGATGATAAAGAGAGGAAGAAAGTCCAGCTATGATGAAG  960
Schil_LA1971_RDR     TTCTTGTTGGCTTGAGACGTTACCGTTTCTTTGTGTATAAAGATGATAAAGAGAGGAAGAAAGTCCAGCTATGATGAAG  960
Schil_LA0130_RDR     TTCTTGTTGGCTTGAGACGTTACCGTTTCTTTGTGTATAAAGATGATAAAGAGAGGAAGAAAGTCCAGCTATGATGAAG  960
Schil_LA1960_RDR     TTCTTGTTGGCTTGAGACGTTACCGTTTCTTTGTGTATAAAGATGATAAAGAGAGGAAGAAAGTCCAGCTATGATGAAG  960
Schil_LA2737_RDR     TTCTTGTTGGCTTGAGACGTTACCGTTTCTTTGTGTATAAAGATGATAAAGAGAGGAAGAAAGTCCAGCTATGATGAAG  960
```

```
Slyc_MM_RDR          ACAAAAACTGCTTCTTTGAAGTGCTACTTTGTTAGGTTTGAGTCCATTGGAACCTGCAATGATGGAGAATCCTATGTATT 1028
Schil_LA1969_Ty1     ACAAAAACTGCTTCTTTGAAGTGCTACTTTGTTAGGTTTGAGTCCATTGGAACCTGCGATGATGGAGAATCCTATGTATT 1040
Schil_LA2779_Ty3     ACAAAAACTGCTTCTTTGAAGTGCTACTTTGTTAGGTTTGAGTCCATTGGAACCTGCGATGATGGAGAATCCTATGTATT 1040
Schil_LA1932_RDR     ACAAAAACTGCTTCTTTGAAGTGCTACTTTGTTAGGTTTGAGTCCATTGGAACCTGCGATGATGGAGAATCCTATGTATT 1040
Schil_LA1938_RDR     ACAAAAACTGCTTCTTTGAAGTGCTACTTTGTTAGGTTTGAGTCCATTGGAACCTGCGATGATGGAGAATCCTATGTATT 1040
Schil_LA1971_RDR     ACAAAAACTGCTTCTTTGAAGTGCTACTTTGTTAGGTTTGAGTCCATTGGAACCTGCGATGATGGAGAATCCTATGTATT 1040
Schil_LA0130_RDR     ACAAAAACTGCTTCTTTGAAGTGCTACTTTGTTAGGTTTGAGTCCATTGGAACCTGCGATGATGGAGAATCCTATGTATT 1040
Schil_LA1960_RDR     ACAAAAACTGCTTCTTTGAAGTGCTACTTTGTTAGGTTTGAGTCCATTGGAACCTGCGATGATGGAGAATCCTATGTATT 1040
Schil_LA2737_RDR     ACAAAAACTGCTTCTTTGAAGTGCTACTTTGTTAGGTTTGAGTCCATTGGAACCTGCGATGATGGAGAATCCTATGTATT 1040

Slyc_MM_RDR          TTCTACCAAAACAATCAGTCAAGCAAGGTGTAAATTCATGCATGTGCATATGGTTTCTAATATGGCAAAATATGCAGCCA 1108
Schil_LA1969_Ty1     TTCTACCAAAACAATCAGTCAAGCAAGGTGTAAATTCATGCATGTGCATATGGTTTCTAATATGGCAAAATATGCAGCCA 1120
Schil_LA2779_Ty3     TTCTACCAAAACAATCAGTCAAGCAAGGTGTAAATTCATGCATGTGCATATGGTTTCTAATATGGCAAAATACGCAGCCA 1120
Schil_LA1932_RDR     TTCTACCAAAACAATCAGTCAAGCAAGGTGTAAATTCATGCATGTGCATATGGTTTCTAATATGGCAAAATACGCAGCCA 1120
Schil_LA1938_RDR     TTCTACCAAAACAATCAGTCAAGCAAGGTGTAAATTCATGCATGTGCATATGGTTTCTAATATGGCAAAATATGCAGCCA 1120
Schil_LA1971_RDR     TTCTACCAAAACAGTCAGTCAAGCAAGGTGTAAATTCATGCATGTGCATATGGTTTCTAATATGGCAAAATATGCAGCCA 1120
Schil_LA0130_RDR     TTCTACCAAAACAATCAGTCAAGCAAGGTGTAAATTCATGCATGTGCATATGGTTTCTAATATGGCAAAATATGCAGCCA 1120
Schil_LA1960_RDR     TTCTACCAAAACAGTCAGTCAAGCAAGGTGTAAATTCATGCATGTGCATATGGTTTCTAATATGGCAAAATATGCAGCCA 1120
Schil_LA2737_RDR     TTCTACCAAAACAGTCAGTCAAGCAAGGTGTAAATTCATGCATGTGCATATGGTTTCTAATATGGCAAAATATGCAGCCA 1120

                     exon 7
Slyc_MM_RDR          GGCTTTCCTTAATTCTATCAAAGACGATTAAGCTTCAAACAGATCTTGATTCTGTCACCATTGAAAGAATTGAAGATATA 1188
Schil_LA1969_Ty1     GGCTTTCCTTAATTCTATCAAAGACTATTAAGCTTCAAGTGGATCTTGATTCTGTCACCATTGAAAGAATCGAAGATATA 1200
Schil_LA2779_Ty3     GGCTTTCCTTAATTCTATCAAAGACTATTAAGCTTCAAGCGGATCTTGATTCTGTCACCATTGAAAGAATCGAAGATATA 1200
Schil_LA1932_RDR     GGCTTTCCTTAATTCTATCAAAGACTATTAAGCTTCAAGCGGATCTTGATTCTGTCACCATTGAAAGAATCGAAGATATA 1200
Schil_LA1938_RDR     GGCTTTCCTTAATTCTATCAAAGACTATTAAGCTTCAAGTGGATCTTGATTCTGTCACCATTGAAAGAATCGAAGATATA 1200
Schil_LA1971_RDR     GGCTTTCCTTAATTCTATCAAAGACTATTAAGCTTCAAGTGGATCTTGATTCTGTCACCATTGAAAGAATCGAAGATATA 1200
Schil_LA0130_RDR     GGCTTTCCTTAATTCTATCAAAGACTATTAAGCTTCAAGTGGATCTTGATTCTGTCACCATTGAAAGAATCGAAGATATA 1200
Schil_LA1960_RDR     GGCTTTCCTTAATTCTATCAAAGACTATTAAGCTTCAAGTGGATCTTGATTCTGTCACCATTGAAAGAATCGAAGATATA 1200
Schil_LA2737_RDR     GGCTTTCCTTAATTCTATCAAAGACTATTAAGCTTCAAGTGGATCTTGATTCTGTCACCATTGAAAGAATCGAAGATATA 1200
```

```
                                       exon 8
Slyc_MM_RDR        CTTTGTCGGGATGAAAATGGTTGTATTATTCAAGATGAAGACGGCGAACCTCGTATACATACTGATGGTACTGGTTTCAT 1268
Schil_LA1969_Ty1   CTTTGTCGGGATGAAAATGGTTGTATTATTCAAGATGAAGACGGCGAACCTCGTATACATACTGATGGTACTGGTTTCAT 1280
Schil_LA2779_Ty3   CTTTGTCGGGATGAAAATGGTTGTATTATTCAAGATGAAGACGGCGAACCTCGTATACATACTGATGGTACTGGTTTCAT 1280
Schil_LA1932_RDR   CTTTGTCGGGATGAAAATGGTTGTATTATTCAAGATGAAGACGGCGAACCTCGTATACATACTGATGGTACTGGTTTCAT 1280
Schil_LA1938_RDR   CTTTGTCGGGATGAAAATGGTTGTATTATTCAAGATGAAGACGGCGAACCTCGTATACATACTGATGGTACTGGTTTCAT 1280
Schil_LA1971_RDR   CTTTGTCAGGATGAAAATGGTTGTATTATTCAAGATGAAGACGGCGAACCTCGTATACATACTGATGGTACTGGTTTCAT 1280
Schil_LA0130_RDR   CTTTGTCGGGATGAAAATGGTTGTATTATTCAAGATGAAGACGGCGAACCTCGTATACATACTGATGGTACTGGTTTCAT 1280
Schil_LA1960_RDR   CTTTGTCAGGATGAAAATGGTTGTATTATTCAAGATGAAGACGGCGAACCTCGTATACATACTGATGGTACTGGTTTCAT 1280
Schil_LA2737_RDR   CTTTGTCGGGATGAAAATGGTTGTATTATTCAAGATGAAGACGGCGAACCTCGTATACATACTGATGGTACTGGTTTCAT 1280

                                                                                             exon 9
Slyc_MM_RDR        ATCAGAAGATTTAGCTATGCATTGTCCCAAAGATTTTTCAAAAGCAGAATATATAAAAGATGAAAATTATGAGAATTTTG 1348
Schil_LA1969_Ty1   ATCAGAAGATTTAGCTATGCATTGTCCCAAAGATTTTTCAAAAGCAGAATATATAAAAGATGAAAATTATGAGAATTTTG 1360
Schil_LA2779_Ty3   ATCAGAAGATTTAGCTATGCATTGTCCCAAAGATTTTTCAAAAGCAGAATATATAAAAGATGAAAATTATGAGAATTTTG 1360
Schil_LA1932_RDR   ATCAGAAGATTTAGCTATGCATTGTCCCAAAGATTTTTCAAAAGCAGAATATATAAAAGATGAAAATTATGAGAATTTTG 1360
Schil_LA1938_RDR   ATCAGAAGATTTAGCTATGCATTGTCCCAAAGATTTTTCAAAAGCAGAATATATAAAAGATGAAAATTATGAGAATTTTG 1360
Schil_LA1971_RDR   ATCAGAAGATTTAGCTATGCATTGTCCCAAAGATTTTTCAAAAGCAGAATATATAAAAGATGAAAATTATGAGAATTTTG 1360
Schil_LA0130_RDR   ATCAGAAGATTTAGCTATGCATTGTCCCAAAGATTTTTCAAAAGCAGAATATATAAAAGATGAAAATTATGAGAATTTTG 1360
Schil_LA1960_RDR   ATCAGAAGATTTAGCTATGCATTGTCCCAAAGATTTTTCAAAAGCAGAATATATAAAAGATGAAAATTATGAGAATTTTG 1360
Schil_LA2737_RDR   ATCAGAAGATTTAGCTATGCATTGTCCCAAAGATTTTTCAAAAGCAGAATATATAAAAGATGAAAATTATGAGAATTTTG 1360

                                                                                         exon 10
Slyc_MM_RDR        TTGATATCGTGGACCTTGATGACGTGAATGTAGAAAGAAGAGTGAGTGTATCTCGCAATAGGAAACCGCCTTTGTTGATG 1428
Schil_LA1969_Ty1   TTGATATCGTGGACCTTGATGACGTGAATGTAGAAAGAAGAGCGAGTGTATCTGGGAATAGGGAACCGCCTTTGTTGATG 1440
Schil_LA2779_Ty3   TTGATATCGTGGACCTTGATGACGTGAATGTAGAAAGAAGAGYGAGTGTATCTCGCAATAGGAAACCGCCTTTGTTGATG 1440
Schil_LA1932_RDR   TTGATATCGTGGACCTTGATGACGTGAATGTAGAAAGAAGAGCGAGTGTATCTGGGAACAGGGAACCGCCTTTGTTGATG 1440
Schil_LA1938_RDR   TTGATATCGTGGACCTTGATGACGTGAATGTAGAAAGAAGAGCGAGTGTATCTGGGAATAGGGAACCGCCTTTGTTGATG 1440
Schil_LA1971_RDR   TTGATATCGTGGACCTTGATGACGTGAATGTAGAAAGAAGAGCGAGTGTATCTGGGAATAGGGAACCGCCTTTGTTGATG 1440
Schil_LA0130_RDR   TTGATATCGTGGACCTTGATGACGTGAATGTAGAAAGAAGAGCGAGTGTATCTGGGAATAGGGAACCGCCTTTGTTGATG 1440
Schil_LA1960_RDR   TTGATATCGTGGACCTTGATGACGTGAATGTAGAAAGAAGAGCGAGTGTATCTGGGAATAGGGAACCGCCTTTGTTGATG 1440
Schil_LA2737_RDR   TTGATATCGTGGACCTTGATGACGTGAATGTAGAAAGAAGAGCGAGTGTATCTGGGAATAGGGAACCGCCTTTGTTGATG 1440
```

```
                                                                                                                    exon 11
Slyc_MM_RDR        CAGTGCCGTTTGTTCTTCAATGGTTGTGCTGTGAAGGGGACTTTTCTTGTCAATAGAAAGATTGGATCACGAAAAATTCA 1508
Schil_LA1969_Ty1   CAGTGCCGTTTGTTCTTCAACGGTTGTGCTGTGAAGGGGACTTTTCTTGTCAATAGAAAGATTGGATCACGAAAAATTCA 1520
Schil_LA2779_Ty3   CAGTGCCGTTTGTTCTTCAAAGGTTGTGCTGTGAAGGGGACTTTTCTTCTCAATAGAAAGATTGGATCACGAAAAATTCA 1520
Schil_LA1932_RDR   CAGTGCCGTTTGTTCTTCAAAGGTTGTGCTGTGAAGGGGACTTTTCTTCTCAATAGAAAGATTGGATCACGAAAAATTCA 1520
Schil_LA1938_RDR   CAGTGCCGTTTGTTCTTCAACGGTTGTGCTGTGAAGGGGACTTTTCTTGTCAATAGAAAGATTGGATCACGAAAAATTCA 1520
Schil_LA1971_RDR   CAGTGCCGTTTGTTCTTCAACGGTTGTGCTGTGAAGGGGACTTTTCTTGTCAATAGAAAGATTGGATCACGAAAAATTCA 1520
Schil_LA0130_RDR   CAGTGCCGTTTGTTCTTCAACGGTTGTGCTGTGAAGGGGACTTTTCTTGTCAATAGAAAGATTGGATCACGAAAAATTCA 1520
Schil_LA1960_RDR   CAGTGCCGTTTGTTCTTCAACGGTTGTGCTGTGAAGGGGACTTTTCTTGTCAATAGAAAGATTGGATCACGAAAAATTCA 1520
Schil_LA2737_RDR   CAGTGCCGTTTGTTCTTCAACGGTTGTGCTGTAAAGGGGACTTTTCTTGTCAATAGAAAGATTGGATCACGAAAAATTCA 1520

Slyc_MM_RDR        TATTAGACCCTCAATGGTGAAGGTTGAGATAGACCCAACAATTTCAAGTATACCAACTTTTGACTCATTGGAGATAGTTG 1588
Schil_LA1969_Ty1   TATTAGACCCTCAATGGTGAAGGTTGAGATAGACCCAACAATTTCAAGTATACCAACTTTTGACTCATTGGAGATAGTTG 1600
Schil_LA2779_Ty3   TATTAGACCCTCAATGGTGAAGGTTGAGATAGACCCAACAATTTCAAGTATACCAACTTTTGACTCATTGGAGATAGTTG 1600
Schil_LA1932_RDR   TATTAGACCCTCAATGGTGAAGGTTGAGATAGACCCAACAATTTCAAGTATACCAACTTTTGACTCATTGGAGATAGTTG 1600
Schil_LA1938_RDR   TATTAGACCCTCAATGGTGAAGGTTGAGATAGACCCAACAATTTCAAGTATACCAACTTTTGACTCATTGGAGATAGTTG 1600
Schil_LA1971_RDR   TATTAGACCCTCAATGGTGAAGGTTGAGATAGACCCAACAATTTCAAGTATACCAACTTTTGACTCATTGGAGATAGTTG 1600
Schil_LA0130_RDR   TATTAGACCCTCAATGGTGAAGGTTGAGATAGACCCAACAATTTCAAGTATACCAACTTTTGACTCATTGGAGATAGTTG 1600
Schil_LA1960_RDR   TATTAGACCCTCAATGGTGAAGGTTGAGATAGACCCAACAATTTCAAGTATACCAACTTTTGACTCATTGGAGATAGTTG 1600
Schil_LA2737_RDR   TATTAGACCCTCAATGGTGAAGGTTGAGATAGACCCAACAATTTCAAGTATACCAACTTTTGACTCATTGGAGATAGTTG 1600

                          exon 12
Slyc_MM_RDR        CAATCAGTCATAGACCAAATAAGGCATATCTGTCCAAGAATTTAATCTCTCTGCTGAGCTACGGAGGAGTCCATAAAGAA 1668
Schil_LA1969_Ty1   CAATCAGTCATAGACCAAATAAGGCATATCTGTCCAAGAATTTAATCTCTCTGCTGAGCTACGGAGGAGTCCATAAAGAA 1680
Schil_LA2779_Ty3   CAATCAGTCATAGACCAAATAAGGCATATCTGTCCAAGAATTTAATCTCTCTGCTGAGCTACGGAGGAGTCCATAAAGAA 1680
Schil_LA1932_RDR   CAATCAGTCATAGACCAAATAAGGCATATCTGTCCAAGAATTTAATCTCTCTGCTGAGCTACGGAGGAGTCCATAAAGAA 1680
Schil_LA1938_RDR   CAATCAGTCATAGACCAAATAAGGCATATCTGTCCAAGAATTTAATCTCTCTGCTGAGCTACGGAGGAGTCCATAAAGAA 1680
Schil_LA1971_RDR   CAATCAGTCATAGACCAAATAAGGCATATCTGTCCAAGAATTTAATCTCTCTGCTGAGCTACGGAGGAGTCCATAAAGAA 1680
Schil_LA0130_RDR   CAATCAGTCATAGACCAAATAAGGCATATTTGTCCAAAAATTTAATCTCTCTGCTGAGCTACGGAGGAGTCCATAAAGAA 1680
Schil_LA1960_RDR   CAATCAGTCATAGACCAAATAAGGCATATCTGTCCAAGAATTTAATCTCTCTGCTGAGCTACGGAGGAGTCCATAAAGAA 1680
Schil_LA2737_RDR   CAATCAGTCATAGACCAAATAAGGCATATCTGTCCAAGAATTTAATCTCTCTGCTGAGCTACGGAGGAGTCCATAAAGAA 1680
```

```
                                                                                             exon13
Slyc_MM_RDR        TACTTTATGGAACTTTTGGGAAGTGCGCTGGAAGAGACGAAACAAGTATATTTGAGGAAACGTGCAGCTCTAAAAGTTGC 1748
Schil_LA1969_Ty1   TACTTTCTGGAGCTTTTGGGAAGTGCACTGGAAGAGACGAAACAAGTATATTTGAGGAAACGGGCAGCTCTAAAAGTTGC 1760
Schil_LA2779_Ty3   TACTTTCTGGAGCTTTTGGGAAGTGCACTGGAAGAGACGAAACAAGTATATTTGAGGAAACGGGCAGCTCTAAAAGTTGC 1760
Schil_LA1932_RDR   TACTTTCTGGAGCTTTTGGGAAGTGCACTGGAAGAGACGAAACAAGTATATTTGAGGAAACGGGCAGCTCTAAAAGTTGC 1760
Schil_LA1938_RDR   TACTTTCTGGAGCTTTTGGGAAGTGCACTGGAAGAGACGAAACAAGTATATTTGAGGAAACGGGCAGCTCTAAAAGTTGC 1760
Schil_LA1971_RDR   TACTTTCTGGAGCTTTTGGGAAGTGCACTGGAAGAGACGAAACAAGTATATTTGAGGAAACGGGCAGCTCTAAAAGTTGC 1760
Schil_LA0130_RDR   TACTTTCTGGAGCTTTTGGGAAGTGCACTGGAAGAGACGAAACAAGTATATTTGAGGAAACGGGCAGCTCTAAAAGTTGC 1760
Schil_LA1960_RDR   TACTTTCTGGAGCTTTTGGGAAGTGCACTGGAAGAGACGAAACAAGTATATTTGAGGAAACGGGCAGCTCTAAAAGTTGC 1760
Schil_LA2737_RDR   TACTTTCTGGAGCTTTTGGGAAGTGCACTGGAAGAGACGAAACAAGTATATTTGAGGAAACGGGCAGCTCTAAAAGTTGC 1760

Slyc_MM_RDR        TATCAACTATAGAGAAATGGATGATGAATGTCTAACAGCAAGGATGATATCGTCTGGGATACCTCTCAATGAACCTCATC 1828
Schil_LA1969_Ty1   TATCAACTATAGAGAAATGGATGATGAATGTCTAACAGCAAGGATGATATCGTCTGGGATACCTCTCAATGAACCTCATC 1840
Schil_LA2779_Ty3   TATCAACTATAGAGAAATGGATGATGAATGTCTAACAGCAAGGATGATATCGTCTGGGATACCTCTCAATGAACCTCATC 1840
Schil_LA1932_RDR   TATCAACTATAGAGAAATGGATGATGAATGTCTAACAGCAAGGATGATATCGTCTGGGATACCTCTCAATGAACCTCATC 1840
Schil_LA1938_RDR   TATCAACTATAGAGAAATGGATGATGAATGTCTAACAGCAAGGATGATATCGTCTGGGATACCTCTCAATGAACCTCATC 1840
Schil_LA1971_RDR   TATCAACTATAGAGAAATGGATGATGAATGTCTAACAGCAAGGATGATATCGTCTGGGATACCTCTCAATGAACCTCATC 1840
Schil_LA0130_RDR   TATCAACTATAGAGAAATGGATGATGAATGTCTAACAGCAAGGATGATATCGTCTGGGATACCTCTCAATGAACCTCATC 1840
Schil_LA1960_RDR   TATCAACTATAGAGAAATGGATGATGAATGTCTAACAGCAAGGATGATATCGTCTGGGATACCTCTCAATGAACCTCATC 1840
Schil_LA2737_RDR   TATCAACTATAGAGAAATGGATGATGAATGTCTAACAGCAAGGATGATATCGTCTGGGATACCTCTCAATGAACCTCATC 1840

Slyc_MM_RDR        TCCATGCTCGCTTGTCTAGGCTTGCAAAGATTGAAAGAACTAAGCTTAGAGGAGGAAAGCTTCCTATAAGTGACAGTTTT 1908
Schil_LA1969_Ty1   TCCATGTTCGCTTGTCTAGGCTTGCAAAGATTGAAAGAACTAAGCTTAGAGGAGGAAAGCTTCCTATAAGTGACAGTTTT 1920
Schil_LA2779_Ty3   TCCATGTTCGCTTGTCTAGGCTTGCAAAGATTGAAAGAACTAAGCTTAGAGGAGGAAAGCTTCCTATAAGTGACAGTTTT 1920
Schil_LA1932_RDR   TCCATGTTCGCTTGTCTAGGCTTGCAAAGATTGAAAGAACTAAGCTTAGAGGAGGAAAGCTTCCTATAAGTGACAGTTTT 1920
Schil_LA1938_RDR   TCCATGTTCGCTTGTCTAGGCTTGCAAAGATTGAAAGAACTAAGCTTAGAGGAGGAAAGCTTCCTATAAGTGACAGTTTT 1920
Schil_LA1971_RDR   TCCATGTTCGCTTGTCTAGGCTTGCAAAGATTGAAAGAACTAAGCTTAGAGGAGGAAAGCTTCCTATAAGTGACAGTTTT 1920
Schil_LA0130_RDR   TCCATGTTCGCTTGTCTAGGCTTGCAAAGATTGAAAGAACTAAGCTTAGAGGAGGAAAGCTTCCTATAAGTGACAGTTTT 1920
Schil_LA1960_RDR   TCCATGTTCGCTTGTCTAGGCTTGCAAAGATTGAAAGAACTAAGCTTAGAGGAGGAAAGCTTCCTATAAGTGACAGTTTT 1920
Schil_LA2737_RDR   TCCATGTTCGCTTGTCTAGGCTTGCAAAGATTGAAAGAACTAAGCTTAGAGGAGGAAAGCTTCCTATAAGTGACAGTTTT 1920
```

exon 14

```
Slyc_MM_RDR        TATCTTATGGGAACAGCTGACCCCACTGGTGTACTGGAAAGCAATGAAGTCTGTGTTATTCTAGATAATGGCCAAGTATC 1988
Schil_LA1969_Ty1   TATCTTATGGGAACAGCTGACCCCACTGGTGTACTGGAAAGCAATGAAGTCTGTGTTATTCTAGATAATGGCCAAGTATC 2000
Schil_LA2779_Ty3   TATCTTATGGGAACAGCTGACCCCACTGGTGTACTGGAAAGCAATGAAGTCTGTGTTATTCTAGATAATGGCCAAGTATC 2000
Schil_LA1932_RDR   TATCTTATGGGAACAGCTGACCCCACTGGTGTACTGGAAAGCAATGAAGTCTGTGTTATTCTAGATAATGGCCAAGTATC 2000
Schil_LA1938_RDR   TATCTTATGGGAACAGCTGACCCCACTGGTGTACTGGAAAGCAATGAAGTCTGTGTTATTCTAGATAATGGCCAAGTATC 2000
Schil_LA1971_RDR   TATCTTATGGGAACAGCTGACCCCACTGGTGTACTGGAAAGCAATGAAGTCTGTGTTATTCTAGATAATGGCCAAGTATC 2000
Schil_LA0130_RDR   TATCTTATGGGAACAGCTGACCCCACTGGTGTACTGGAAAGCAATGAAGTCTGTGTTATTCTAGATAATGGCCAAGTATC 2000
Schil_LA1960_RDR   TATCTTATGGGAACAGCTGACCCCACTGGTGTACTGGAAAGCAATGAAGTCTGTGTTATTCTAGATAATGGCCAAGTATC 2000
Schil_LA2737_RDR   TATCTTATGGGAACAGCTGACCCCACTGGTGTACTGGAAAGCAATGAAGTCTGTGTTATTCTAGATAATGGCCAAGTATC 2000

Slyc_MM_RDR        TGGGCGTGTTTTGGTCTATAGAAATCCTGGTCTTCACTTTGGAGATGTACATGTGATGAAAGCGCGATATGTGGAAGAGC 2068
Schil_LA1969_Ty1   TGGGCGTGTTTTGGTCTACAGAAATCCTGGTCTTCACTTTGGAGATGTGCATGTGATGAAAGCGCGATATGTGGAAGAGC 2080
Schil_LA2779_Ty3   TGGGCGTGTTTTGGTCTACAGAAATCCTGGTCTTCACTTTGGAGATGTGCATGTGATGAAAGCGCGATATGTGGAAGAGC 2080
Schil_LA1932_RDR   TGGGCGTGTTTTGGTCTACAGAAATCCTGGTCTTCACTTTGGAGATGTGCATGTGATGAAAGCGCGATATGTGGAAGAGC 2080
Schil_LA1938_RDR   TGGGCGTGTTTTGGTCTACAGAAATCCTGGTCTTCACTTTGGAGACGTGCATGTGATGAAAGCGCGATATGTGGAAGAGC 2080
Schil_LA1971_RDR   TGGGCGTGTTTTGGTCTACAGAAATCCTGGTCTTCACTTTGGAGATGTGCATGTGATGAAAGCGCGATATGTGGAAGAGC 2080
Schil_LA0130_RDR   TGGGCGTGTTTTGGTCTACAGAAATCCTGGTCTTCACTTTGGAGACGTGCATGTGATGAAAGCGCGATATGTGGAAGAGC 2080
Schil_LA1960_RDR   TGGGCGTGTTTTGGTCTACAGAAATCCTGGTCTTCACTTTGGAGATGTGCATGTGATGAAAGCGCGATATGTGGAAGAGC 2080
Schil_LA2737_RDR   TGGGCGTGTTTTGGTCTACAGAAATCCTGGTCTTCACTTTGGAGACGTGCATGTGATGAAAGCGCGATATGTGGAAGAGC 2080

Slyc_MM_RDR        TTGCAGATGTTGTTGGTGATGCCAAATATGGTATATTTTTTTCAACTAAAGGCCCGAGGTCAGCTGCTACTGAGATTGCA 2148
Schil_LA1969_Ty1   TTGCAGATGTTGTTGGTGATGCCAAATATGGTATATTTTTTTCAACTAAAGGCCCGAGGTCAGCTGCTACTGAGATTGCA 2160
Schil_LA2779_Ty3   TTGCAGATGTTGTTGGTGATGCCAAATATGGTATATTTTTTTCAACTAAAGGCCCGAGGTCAGCTGCTACTGAGATTGCA 2160
Schil_LA1932_RDR   TTGCAGATGTTGTTGGTGATGCCAAATATGGTATATTTTTTTCAACTAAAGGCCCGAGGTCAGCTGCTACTGAGATTGCA 2160
Schil_LA1938_RDR   TTGCAGATGTTGTTGGTGATGCCAAATATGGTATATTTTTTTCAACTAAAGGCCCGAGGTCAGCTGCTACTGAGATTGCA 2160
Schil_LA1971_RDR   TTGCAGATGTTGTTGGTGATGCCAAATATGGTATATTTTTTTCAACTAAAGGCCCGAGGTCAGCTGCTACTGAGATTGCA 2160
Schil_LA0130_RDR   TTGCAGATGTTGTTGGTGATGCCAAATATGGTATATTTTTTTCAACTAAAGGCCCGAGGTCAGCTGCCACTGAGATTGCA 2160
Schil_LA1960_RDR   TTGCAGATGTTGTTGGTGATGCCAAATATGGTATATTTTTTTCAACTAAAGGCCCGAGGTCAGCTGCTACTGAGATTGCA 2160
Schil_LA2737_RDR   TTGCAGATGTTGTTGGTGATGCCAAATATGGTATATTTTTTTCAACTAAAGGCCCGAGGTCAGCTGCTACTGAGATTGCA 2160
```

```
                                                          exon 15
Slyc_MM_RDR       AATGGTGACTTTGATGGTGATATGTATTGGGTTTCCATAAACCGTAAGTTGGTAGATTCTTATACAACAAGTAGACCATG 2228
Schil_LA1969_Ty1  AATGGTGACTTTGATGGTGATATGTATTGGGTTTCCATAAACCGTAAGTTGGTAGATTCTTATACAACAAGTAGACCATG 2240
Schil_LA2779_Ty3  AATGGTGACTTTGATGGTGATATGTATTGGGTTTCCATAAACCGTAAGTTGGTAGATTCTTATACAACAAGTAGACCATG 2240
Schil_LA1932_RDR  AATGGTGACTTTGATGGTGATATGTATTGGGTTTCCATAAACCGTAAGTTGGTAGATTCTTATACAACAAGTAGACCATG 2240
Schil_LA1938_RDR  AATGGTGACTTTGATGGTGATATGTATTGGGTTTCCATAAACCGTAAGTTGGTAGATTCTTATACAACAAGTAGACCATG 2240
Schil_LA1971_RDR  AATGGTGACTTTGATGGTGATATGTATTGGGTTTCCATAAACCGTAAGTTGGTAGATTCTTATACAACAAGTAGACCATG 2240
Schil_LA0130_RDR  AATGGTGACTTTGATGGTGATATGTATTGGGTTTCCATAAACCGTAAGTTGGTAGATTCTTATACAACAAGTAGACCATG 2240
Schil_LA1960_RDR  AATGGTGACTTTGATGGTGATATGTATTGGGTTTCCATAAACCGTAAGTTGGTAGATTCTTATACAACAAGTAGACCATG 2240
Schil_LA2737_RDR  AATGGTGACTTTGATGGTGATATGTATTGGGTTTCCATAAACCGTAAGTTGGTAGATTCTTATACAACAAGTAGACCATG 2240

Slyc_MM_RDR       GATTCGCATGCATTCAACTCCTAACGCAGTTAGCAAAAAACCAAGTGAATTTTCAGCTGATGAATTGGAATATGAGCTTT 2308
Schil_LA1969_Ty1  GATTCGCATGCATTCAACTCCTAAGGCAGTTAGCAAAAAACCAAGTGAATTTTCAGCTGATGAATTGGAATATGAGCTTT 2320
Schil_LA2779_Ty3  GATTCGCATGCATTCAACTCCTAAGGCAGTTAGCAAAAAACCAAGTGAATTTTCAGCTGATGAATTGGAATATGAGCTTT 2320
Schil_LA1932_RDR  GATTCGCATTCATTCAACTCCTAAGGCAGTTAGCAAAAAACCAAGTGAATTTTCAGCTGATGAATTGGAATATGAGCTTT 2320
Schil_LA1938_RDR  GATTCGCATGCATTCAACTCCTAAGGCAGTTAGCAAAAAGCCAAGTGAATTTTCAGCTGATGAATTGGAATATGAGCTTT 2320
Schil_LA1971_RDR  GATTCGCATGCATTCAACTCCTAAGGCAGTTAGCAAAAAACCAAGTGAATTTTCAGCTGATGAATTGGAATATGAGCTTT 2320
Schil_LA0130_RDR  GATTCGCATGCATTCAACTCCTAAGGCAGTTAGCAAAAAACCAAGTGAATTTTCAGCTGATGAATTGGAATATGAGCTTT 2320
Schil_LA1960_RDR  GATTCGCATGCATTCAACTCCTAAGGCAGTTAGCAAAAAACCAAGTGAATTTTCAGCTGATGAATTGGAATATGAGCTTT 2320
Schil_LA2737_RDR  GATTCGCATGCATTCAACTCCTAAGGCAGTTAGCAAAAAACCAAGTGAATTTTCAGCTGATGAATTGGAATATGAGCTTT 2320

                                                   exon 16
Slyc_MM_RDR       TTAGGCAATTTCTGGAAGCAAAGTCTAAAGGTGCCAATATGTCTCTGGCAGCTGATAGCTGGCTGGCATTTATGGATCGT 2388
Schil_LA1969_Ty1  TCAGGCAATTTCTGGAAGCAAAGTCTAAAGGTGCCAATATGTCTCTGGCAGCTGATAGCTGGCTGGCATTTATGGATCGT 2400
Schil_LA2779_Ty3  TTAGGCAATTTCTGGAAGCAAAGTCTAAAGGTGCCAATATGTCTCTGGCAGCTGATAGCTGGCTGGCATTTATGGATCGT 2400
Schil_LA1932_RDR  TTAGGCAATTTCTGGAAGCAAAGTCTAAAGGTGCCAATATGTCTCTGGCAGCTGATAGCTGGCTGGCATTTATGGATCGT 2400
Schil_LA1938_RDR  TTAGGCAATTTCTGGAAGCAAAGTCTAAAGGTGCCAATATGTCTCTGGCAGCTGATAGCTGGCTGGCATTTATGGATCGT 2400
Schil_LA1971_RDR  TTAGGCAATTTCTGGAAGCAAAGTCTAAAGGTGCCAATATGTCTCTGGCAGCTGATAGCTGGCTGGCATTTATGGATCGT 2400
Schil_LA0130_RDR  TTAGGCAATTTCTGGAAGCAAAGTCTAAAGGTGCCAATATGTCTCTGGCAGCTGATAGCTGGCTGGCATTTATGGATCGT 2400
Schil_LA1960_RDR  TTAGGCAATTTCTGGAAGCAAAGTCTAAAGGTGCCAATATGTCTCTGGCAGCTGATAGCTGGCTGGCATTTATGGATCGT 2400
Schil_LA2737_RDR  TTAGGCAATTTCTGGAAGCAAAGTCTAAAGGTGCCAATATGTCTCTGGCAGCTGATAGCTGGCTGGCATTTATGGATCGT 2400
```

```
Slyc_MM_RDR        CTTCTGACGCTGCGAGATGATAATGTGGATGATATGCATAGCTTGAAAGGCAAGATGCTTCACCTGATTGACATCTACTA 2468
Schil_LA1969_Ty1   CTTCTGATGCTGCGAGATGATAATGTGGATGATATGCATAGCTTGAAAGGCAAGATGCTTCACCTGATTGACATCTACTA 2480
Schil_LA2779_Ty3   CTTCTGATGCTGCGAGATGATAATGTGGATGATATGCATAGATTGAAAGGCAAGATGCTTCACTTGATTGACATCTACTA 2480
Schil_LA1932_RDR   CTTCTGATGCTGCGAGATGATAATGTGGATGATATGCATAGCTTGAAAGGCAAGATGCTTCACTTGATTGACATCTACTA 2480
Schil_LA1938_RDR   CTTCTGATGCTGCGAGATGATAATGTGGATGATATGCATAGCTTGAAAGGCAAGATGCTTCACCTGATTGACATCTACTA 2480
Schil_LA1971_RDR   CTTCTGATGCTGCGAGATGATAATGTGGATGATATGCATAGCTTGAAAGGCAAGATGCTTCACCTGATTGACATCTACTA 2480
Schil_LA0130_RDR   CTTCTGATGCTGCAAGATGATAATGTGGATGATATGCATAGCTTGAAAGGCAAGATGCTTCACCTGATTGACATCTACTA 2480
Schil_LA1960_RDR   CTTCTGATGCTGCGAGATGATAATGTGGATGATATGCATAGCTTGAAAGGCAAGATGCTTCACCTGATTGACATCTACTA 2480
Schil_LA2737_RDR   CTTCTGATGCTGCGAGATGATAATGTGGATGATATGCATAGCTTGAAAGGCAAGATGCTTCACCTGATTGACATCTACTA 2480

                                                  exon 17
Slyc_MM_RDR        TGATGCATTAGATGCACCTAAAAGCGGGAAGAAGGTTAGCATCCCTCATTATCTGAAGGCAAACAAGTTCCCCCACTATA 2548
Schil_LA1969_Ty1   TGATGCATTAGATGCACCTAAAAGCGGGAAGAAGGTTAGCATCCCTCATTATCTGAAGGCAAACAAGTTCCCCCACTATA 2560
Schil_LA2779_Ty3   TGATGCATTAGATGCACCTAAAAGCGGGAAGAAGGTTAGCATCCCTCATTATCTGAAGGCAAACAAGTTCCCCCACTATA 2560
Schil_LA1932_RDR   TGATGCATTAGATGCACCTAAAAGCGGGAAGAAGGTTAGCATCCCTCATTATCTGAAGGCAAACAAGTTCCCCCACTATA 2560
Schil_LA1938_RDR   TGATGCATTAGATGCACCTAAAAGCGGGAAGAAGGTTAGCATCCCTCATTATCTGAAAGCAAACAAGTTCCCCCACTATA 2560
Schil_LA1971_RDR   TGATGCATTAGATGCACCTAAAAGCGGGAAGAAGGTTAGCATCCCTCATTATCTGAAGGCAAACAAGTTCCCCCACTATA 2560
Schil_LA0130_RDR   TGATGCATTAGATGCACCTAAAAGCGGGAAGAAGGTTAGCATCCCTCATTATCTGAAAGCAAACAAGTTCCCCCACTATA 2560
Schil_LA1960_RDR   TGATGCATTAGATGCACCTAAAAGCGGGAAGAAGGTTAGCATCCCTCATTATCTGAAGGCAAACAAGTTCCCCCACTATA 2560
Schil_LA2737_RDR   TGATGCATTAGATGCACCTAAAAGCGGGAAGAAGGTTAGCATCCCTCATTATCTGAAAGCAAACAAGTTCCCCCACTATA 2560

Slyc_MM_RDR        TGGAAAAAGGGAACTCCTGCAGCTATCATTCAACTTCTATTCTGGGTCAGATTTATGATCATGTCGACTCATATCCAGAT 2628
Schil_LA1969_Ty1   TGGAAAAAGGGAACTCCTGCAGCTATCATTCAACTTCTATTCTGGGTCAGATTTATGATCATGTCGACTCATATCCAGAT 2640
Schil_LA2779_Ty3   TGGAAAAAGGGAACTCCTGCAGCTATCATTCAACTTCTATTCTGGGTCAGATTTATGATCATGTCGACTCATATCCAGAT 2640
Schil_LA1932_RDR   TGGAAAAAGGGAACTCCTGCAGCTATCATTCAACTTCTATTCTGGGTCAGATTTATGATCATGTCGACTCATATCCAGAT 2640
Schil_LA1938_RDR   TGGAAAAAGGGAACTCCTGCAGCTATCATTCAACTTCTATTCTGGGTCAGATTTATGATCATGTCGACTCATATCCAGAT 2640
Schil_LA1971_RDR   TGGAAAAAGGGAACTCCTGCAGCTATCATTCAACTTCTATTCTGGGTCAGATTTATGATCATGTCGACTCATATCCAGAT 2640
Schil_LA0130_RDR   TGGAAAAAGGGAACTCCTGCAGCTATCATTCAACTTCTATTCTGGGTCAGATTTATGATCATGTCGACTCATATCCAGAT 2640
Schil_LA1960_RDR   TGGAAAAAGGGAACTCCTGCAGCTATCATTCAACTTCTATTCTGGGTCAGATTTATGATCATGTCGACTCATATCCAGAT 2640
Schil_LA2737_RDR   TGGAAAAAGGGAACTCCTGCAGCTATCATTCAACTTCTATTCTGGGTCAGATTTATGATCATGTCGACTCATATCCAGAT 2640
```

```
                                          exon 18
Slyc_MM_RDR         GAAGATTTGTGTATAACAGAATCTCTAAACTGCCTTGCTTTGAAGTTGAAATCCCTCAAAGATGCATGACATTGTGGAG 2708
Schil_LA1969_Ty1    GAAGATTTGTGTATAACAGAGATCTCTAAACTGCCTTGCTTTGAAGTTGAAATCCCTCAAAGATGCATGACATTGTGGAG 2720
Schil_LA2779_Ty3    GAAGATTTGTGCATAACAGAGATCTCTAAACTGCCTTGCTTTGAAGTTGAAATCCCTCAAAGATGCATGACATTGTGGAG 2720
Schil_LA1932_RDR    GAAGATTTGTGCATAACAGAGATCTCTAAACTGCCTTGCTTTGAAGTTGAAATCCCTCAAAGATGCATGACATTGTGGAG 2720
Schil_LA1938_RDR    GAAGATTTGTGTATAACAGAGATCTCTAAACTGCCTTGCTTTGAAGTTGAAATCCCTCAAAGATGCATGACATTGTGGAG 2720
Schil_LA1971_RDR    GAAGATTTGTGTATAACAGAGATCTCTAAACTGCCTTGCTTTGAAGTTGAAATCCCTCAAAGATGCATGACATTGTGGAG 2720
Schil_LA0130_RDR    GAAGATTTGTGTATAACAGAGATCTCTAAACTGCCTTGCTTTGAAGTTGAAATCCCTCAAAGATGCATGACATTGTGGAG 2720
Schil_LA1960_RDR    GAAGATTTGTGTATAACAGAGATCTCTAAACTGCCTTGCTTTGAAGTTGAAATCCCTCAAAGATGCATGACATTGTGGAG 2720
Schil_LA2737_RDR    GAAGATTTGTGTATAACAGAGATCTCTAAACTGCCTTGCTTTGAAGTTGAAATCCCTCAAAGATGCATGACATTGTGGAG 2720

Slyc_MM_RDR         AGGAAGATATGAAGAGTACAAAAAGGATATGACACGGCCATGAACTTTGATTGTGAACTAAGAATCACCTCTTGCAATG 2788
Schil_LA1969_Ty1    AGGAAGATATGAAGAGTACAAAAAGGATATGACACAGGCCATGAACTTAGATTGTGAACTTAGAATCACCTCTTGCAATG 2800
Schil_LA2779_Ty3    AGGAAGATATGAAGAGTACAAAAAGGATATGACACAGGCCATGAACTTAGATTGTGAACTTAGAATCACCTCTTGCAATG 2800
Schil_LA1932_RDR    AGGAAGATATGAAGAGTACAAAAAGGATATGACACAGGCCATGAACTTAGATTGTGAACTTAGAATCACCTCTTGCAATG 2800
Schil_LA1938_RDR    AGGAAGATATGAAGAGTACAAAAAGGATATGACACAGGCCATGAACTTAGATTGTGAACTTAGAATCACCTCTTGCAATG 2800
Schil_LA1971_RDR    AGGAAGATATGAAGAGTACAAAAAGGATATGACACAGGCCATGAACTTAGATTGTGAACTTAGAATCACCTCCTGCAATG 2800
Schil_LA0130_RDR    AGGAAGATATGAAAAGTACAAAAAGGATATGACACAGGCCATGAACTTAGATTGTGAACTTAGAATCACCTCTTGCAATG 2800
Schil_LA1960_RDR    AGGAAGATATGAAGAGTACAAAAAGGATATGACACAGGCCATGAACTTAGATTGTGAACTTAGAATCACCTCCTGCAATG 2800
Schil_LA2737_RDR    AGGAAGATATGAAGAGTACAAAAAGGATATGACACAGGCCATGAACTTAGATTGTGAACTTAGAATCACCTCTTGCAATG 2800

                                              exon 19
Slyc_MM_RDR         AAGTTATAAAGAAGTACAAGATGTTGCTATATGGTGCTGTGGAGTTTGAACAAACAGTAAGAAAGACTGAAGACATTTTC 2868
Schil_LA1969_Ty1    AAGTTATAAAGAAGTACAAGATGTTGCTATATGGTGCTGTGGAGTTTGAACAAACAGTAAGAAAGACTGAAGACATTTTC 2880
Schil_LA2779_Ty3    AAGTTATAAAGAAGTACAAGATGTTGCTATATGGTGCTGTGGAGTTTGAACAAACAGTAAGAAAGACTGAAGACATTTTC 2880
Schil_LA1932_RDR    AAGTTATAAAGAAGTACAAGATGTTGCTATATGGTGCTGTGGAGTTTGAACAAACAGTAAGAAAGACTGAAGACATTTTC 2880
Schil_LA1938_RDR    AAGTTATAAAGAAGTACAAGATGTTGCTATATGGTGCTGTGGAGTTTGAACAAACAGTAAGAAAGACTGAAGACATTTTC 2880
Schil_LA1971_RDR    AAGTTATAAAGAAGTACAAGATGTTGCTATATGGTGCTGTAGAGTTTGAACAAACAGTAAGAAAGACTGAAGACATTTTT 2880
Schil_LA0130_RDR    AAGTTATAAAGAAGTACAAGATGTTGCTATATGGTGCTGTGGAGTTTGAACAAACAGTAAGAAAGACTGAAGACATTTTC 2880
Schil_LA1960_RDR    AAGTTATAAAGAAGTACAAGATGTTGCTATATGGTGCTGTAGAGTTTGAACAAACAGTAAGAAAGACTGAAGACATTTTT 2880
Schil_LA2737_RDR    AAGTTATAAAGAAGTACAAGATGTTGCTATATGGTGCTGTGGAGTTTGAACAAACAGTAAGAAAGACTGAAGACATTTTC 2880
```

```
Slyc_MM_RDR          GACGAGGCCCTTGCAATATATCATGTAACATATGATAATGCAAGGATCACATACAGCATAGAGAAATGTGGTTTTGCTTG 2948
Schil_LA1969_Ty1     GATGAAGCCCTTGCAATATATCATGTAACATATGATAATGCAAGGATCACATACAGCATAGAGAAATGTGGTTTTGCTTG 2960
Schil_LA2779_Ty3     GATGAGGCCCTTGCAATATATCATGTAACATATGATAATGCAAGGATCACATACAGCATAGAGAAATGTGGTTTTGCTTG 2960
Schil_LA1932_RDR     GATGAGGCCCTTGCAATATATCATGTAACATATGATAATGCAAGGATCACATACAGCATAGAGAAATGTGGTTTTGCTTG 2960
Schil_LA1938_RDR     GATGAGGCCCTTGCAATATATCATGTAACATATGATAATGCAAGGATCACATACAGCATAGAGAAATGTGGTTTTGCTTG 2960
Schil_LA1971_RDR     GATGAGGCCCTTGCAATATATCATGTAACATATGATAATGCAAGGATCGCATACAGCATAGAGAAATGTGGTTTTGCTTG 2960
Schil_LA0130_RDR     GATGAGGCCCTTGCAATATATCATGTAACATATGATAATGCAAGGATCACATACAGCATAGAGAAATGTGGTTTTGCTTG 2960
Schil_LA1960_RDR     GATGAGGCCCTTGCAATATATCATGTAACATATGATAATGCAAGGATCACATACAGCATAGAGAAATGTGGTTTTGCTTG 2960
Schil_LA2737_RDR     GATGAGGCCCTTGCAATATATCATGTAACATATGATAATGCAAGGATCACATACAGCATAGAGAAATGTGGTTTTGCTTG 2960

Slyc_MM_RDR          GAAAGTAGCTGGTTCTGCGCTTTGCAGGATCCACGCCATGTATCGCAAGGAAAAAGACTTGCCCATTTTGCCATCGGTTT 3028
Schil_LA1969_Ty1     GAAAGTAGCTGGTTCTGCGCTTTGCAGGATCCACGCCATGTATCGCAAGGAAAAAGACTTGCCCATTTTGCCATCGGTTT 3040
Schil_LA2779_Ty3     GAAAGTAGCTGGTTCTGCGCTTTGCAGGATCCACGCCATGTATCACAAGGAAAAAGACTTGCCCATTTTGCCATCGGTTT 3040
Schil_LA1932_RDR     GAAAGTAGCTGGTTCTGCGCTTTGCAGGATCCACGCCATGTATCACAAGGAAAAAGACTTGCCCATTTTGCCATCGGTTT 3040
Schil_LA1938_RDR     GAAAGTAGCTGGTTCTGCGCTTTGCAGGATCCACGCCATGTATCGCAAGGAAAAAGACTTGCCCATTTTGCCATCGGTTT 3040
Schil_LA1971_RDR     GAAAGTAGCTGGTTCTGCGCTTTGCAGGATCCACGCCATGTATCGCAAGGAAAAAGACTTGCCCATTTTGCCATCGGTTT 3040
Schil_LA0130_RDR     GAAAGTAGCTGGTTCTGCGCTTTGCAGGATCCACGCCATGTATCGCAAGGAAAAAGACTTGCCCATTTTACCATCGGTTT 3040
Schil_LA1960_RDR     GAAAGTAGCTGGTTCTGCGCTTTGCAGGATCCACGCCATGTATCGCAAGGAAAAAGACTTGCCCATTTTACCATCGGTTT 3040
Schil_LA2737_RDR     GAAAGTAGCTGGTTCTGCGCTTTGCAGGATCCACGCCATGTATCGCAAGGAAAAAGACTTGCCCATTTTGCCATCGGTTT 3040

Slyc_MM_RDR          TGCAGGAAATACTCTAG 3045
Schil_LA1969_Ty1     TGCAGGAAATACTCTAG 3057
Schil_LA2779_Ty3     TGCAGGAAATACTCTAG 3057
Schil_LA1932_RDR     TGCAGGAAATACTCTAG 3057
Schil_LA1938_RDR     TGCAGGAAATACTCTAG 3057
Schil_LA1971_RDR     TGCAGGAAATACTCTAG 3057
Schil_LA0130_RDR     TGCAGGAAATACTCTAG 3057
Schil_LA1960_RDR     TGCAGGAAATACTCTAG 3057
Schil_LA2737_RDR     TGCAGGAAATACTCTAG 3057
```
