# Supplementary material for: Resistance to Tomato Yellow Leaf Curl Virus in Tomato Germplasm
Source: Front Plant Sci. 2018 Aug 20;9:1198. doi: 10.3389/fpls.2018.01198 (PMC6110163; doi:10.3389/fpls.2018.01198)
Supplement: FIGURE S3 — Alignment of protein sequences of the Ty-1/Ty-3 alleles. Protein sequences are derived from cDNA sequences in Supplementary Figure S2. Unique amino acids present in either LA1960 or LA0130 are highlighted in yellow. The three amino acids specific for the Ty-1/Ty-3 allele are marked in red. [file Image_3.pdf]

```
Slyc_MM_RDR        MGDPLIEEIDV----LDAPLPYSVETMLDRICKEQGQKPPCTGIRRRLSSIGEKGSLEMLKIISRRPIKKSLSAFLVYMI 76
Schil_Ty1_MV       MGDPLIEEIDVPSCILDAPLPYSVETMLDRICKEQGQKPPCTGIRRRLSSIGEKGSLEMLKIISRRPIKKSLSAFLVYMI 80
Schil_Ty3_MV       MGDPLIEEIDVPSCILDAPLPYSVETMLDRICKEQGQKPPCTGIRRRLSSIGEKGSLEMLKIISRRPIKKSLSAFLVYMI 80
Schil_LA1932_RDR   MGDPLIEEIDVPSCILDAPLPYSVETMLDRICKEQGQKPPCTGIRRRLSSIGEKGSLEMLKIISRRPIKKSLSAFLVYMI 80
Schil_LA1938_RDR   MGDPLIEEIDVPSCILDAPLPYSVETMLDRICKEQGQKPPCTGIRRRLSSIGEKGSLEMLKIISRRPIKKSLSAFLVYMI 80
Schil_LA1971_RDR   MGDPLIEEIDVPSCILDAPLPYSVETMLDRICKEQGQKPPCTGIRRRLSSIGEKGSLEMLKIISRRPIKKSLSAFLVYMI 80
Schil_LA0130_RDR   MGDPLIEEIDVPSCILDAPLPYSVETMLDRICKEQGQKPPCTGIRRRLSSIGEKGSLEMLKIISRRPIKKSLSAFLVYMI 80
Schil_LA1960_RDR   MGDPLIEEIDVPSCILDAPLPFSVETMLDRICKEQGQKPPCTGIRRRLSSIGEKGSLEMLKIISRRPIKKSLSAFLVYMI 80
Schil_LA2737_RDR   MGDPLIEEIDVPSCILDAPLPYSVETMLDRICKEQGQKPPCTGIRRRLSSIGEKGSLEMLKIISRRPIKKSLSAFLVYMI 80

Slyc_MM_RDR        DRYPDCLSSSSSPFNCLLKRSSSPRLFPSPEGKRLQGESSSKSKLEMGLLACASPQKVARQLSFCEEPESNCRRTSPYVS 156
Schil_Ty1_MV       DRYPDCLSSSSSPFNSLLKRSSSPVLFPSPEGKRLQGESSSKSKLEMGLLACASPQKVARQLSFCEEPESNCRRTSPYVS 160
Schil_Ty3_MV       DRYPDCLSSSSSPFNSLLKRSSSPLLFPSPEGKRLLGESSSKSKLEMGLLACASPQKVARQLSFCEEPESNCRRTSPYVS 160
Schil_LA1932_RDR   DRYPDCLSSSSSPSNSLLKRSSSPLLFPSPEGKRLQGESSSKSKLEMGLLACASPQKVARQLSFCEEPESNCRRTSPYVS 160
Schil_LA1938_RDR   DRYPDCLSSSSSPFNSLLKRSSSPVLFPSPEGKRLQGESSSKSKLEMGLLACASPQKVARQLSFCEEPESNCRRTSPYVS 160
Schil_LA1971_RDR   DRYPDCLSSSSSPSNSLLKRSSSPVLFPSPEGKRLQGESSSKSKLEMGLLACASPQKVARQLSFCEDPEANCRRTSPYVS 160
Schil_LA0130_RDR   DRYPDCLSSSSSPSNSLLKRSSSPVLFPSPEGKRLQGESSSKSKLEMGLLACASPQKVVRQLSFCEEPESNCRRTSPYVS 160
Schil_LA1960_RDR   DRYPDCLSSSSSPFNSLLKRSSSPVLFPSPEGKRLQGESSSKSKLEMGLLACASPQKVARQLSFCEDPEANCRRTSPYVS 160
Schil_LA2737_RDR   DRYPDCLSSSSSPFNSLLKRSSSPVLFPSPEGKRLQGESSSKSKLEMGLLACASPQKVARQLSFCEEPESNCRRTSPYVS 160

Slyc_MM_RDR        QQLMILNELEFRKLFLVLSYIGCNKLEDVISPQIADDIVRKKNLSMTDFESEIWNAFGKACYAVSDRSKYLDWNCRKTHI 236
Schil_Ty1_MV       QQLMILNELEFRKLFLVLSYIGCNKLEDVISPQIADDIVRKKDLSMTDFESEIWNAFGKACYAVSDRSKYLDWNCRKTHI 240
Schil_Ty3_MV       QQLMILNELEFRKLFLVLSYIGCNKLEDVISPQIADDIVRKKDLSMTDFESEIWNAFGKACYAVSDRSKYLDWNCRKTHI 240
Schil_LA1932_RDR   QQLMILNELEFRKLFLVLSYIGCNKLEEVISPQIADDIVRKKNLSMTDFESEIWNAFGKACYAVSDRSKYLDWNCRKTHI 240
Schil_LA1938_RDR   QQLMILNELEFRKLFLVLSYIGCNKLEDVISPQIADDIVRKKDLSMTDFESEIWNAFGKACYAVSDRSKYLDWNCRKTHI 240
Schil_LA1971_RDR   QQLMILNELEFRKLFLVLSYIGCNKLEDVISPQIADDIVRKKDLSMTDFESEIWNAFGKACYAVSDRSKYLDWNCRKTHI 240
Schil_LA0130_RDR   QQLMILNELEFRKLFLVLSYIGCNKLEDVISPQIADDIVRKKDLSMTDFESEIWNAFGKACYAVSDRSKYLDWNCRKTHI 240
Schil_LA1960_RDR   QQLMILNELEFRKLFLVLSYIGCNKLEDVISPQIADDIVRKKDLSMTDFESEIWNAFGKACYAVSDRSKYLDWNCRKTHI 240
Schil_LA2737_RDR   QQLMILNELEFRKLFLVLSYIGCNKLEDVISPQIADDIVRKKDLSMTDFESEIWNAFGKACYAVSDRSKYLDWNCRKTHI 240
```

```
Slyc_MM_RDR        YYCHIKQNGYCSFKGPYLNTLRTHLQRALGDDNVLIVKFVEDTSCANIILEEGILVGLRRYRFFVYKDDKERKKSPAMMK 316
Schil_Ty1_MV       YYCHIKQNGCCTFKGPYLNTARTHLQRALGDDNVLIVKFVEDTSCANIILEEGILVGLRRYRFFVYKDDKERKKSPAMMK 320
Schil_Ty3_MV       YYCHIKQNGCCTFKGPYLNTARTHLQRALGDDNVLIVKFVEDTSCANIILEEGILVGLRRYRFFVYKDDKERKKSPAMMK 320
Schil_LA1932_RDR   YYCHIKQNGCCTFKGPYLNTARTHLQRALGDDNVLIVKFVEDTSCANIILEEGILVGLRRYRFFVYKDDKERKKSPAMMK 320
Schil_LA1938_RDR   YYCHIKQNGCCTFKGPYLNTARTHLQRALGDDNVLIVKFVEDTSCANIILEEGILVGLRRYRFFVYKDDKERKKSPAMMK 320
Schil_LA1971_RDR   YYCHIKQNGCCTFKGPYLNTARTHLQRALGDDNVLIVKFVEDTSCANIILEEGILVGLRRYRFFVYKDDKERKKSPAMMK 320
Schil_LA0130_RDR   YYCHIKQNGCCTFKGPYLNTARTHLQRALGDDNVLIVKFVEDTSCANIILEEGILVGLRRYRFFVYKDDKERKKSPAMMK 320
Schil_LA1960_RDR   YYCHIKQNGCCTFKGPYLNTARTHLQRALGDDNVLIVKFVEDTSCANIILEEGILVGLRRYRFFVYKDDKERKKSPAMMK 320
Schil_LA2737_RDR   YYCHIKQNGCCTFKGPYLNTARTHLQRALGDDNVLIVKFVEDTSCANIILEEGILVGLRRYRFFVYKDDKERKKSPAMMK 320

Slyc_MM_RDR        TKTASLKCYFVRFESIGTCNDGESYVFSTKTISQARCKFMHVHMVSNMAKYAARLSLILSKTIKLQTDLDSVTIERIEDI 396
Schil_Ty1_MV       TKTASLKCYFVRFESIGTCDDGESYVFSTKTISQARCKFMHVHMVSNMAKYAARLSLILSKTIKLQVDLDSVTIERIEDI 400
Schil_Ty3_MV       TKTASLKCYFVRFESIGTCDDGESYVFSTITISQARCKFMHVHMVSNMAKYAARLSLILSKTIKLQVDLDSVTIERIEDI 400
Schil_LA1932_RDR   TKTASLKCYFVRFESIGTCDDGESYVFSTKTISQARCKFMHVHMVSNMAKYAARLSLILSKTIKLQADLDSVTIERIEDI 400
Schil_LA1938_RDR   TKTASLKCYFVRFESIGTCDDGESYVFSTKTISQARCKFMHVHMVSNMAKYAARLSLILSKTIKLQVDLDSVTIERIEDI 400
Schil_LA1971_RDR   TKTASLKCYFVRFESIGTCDDGESYVFSTKTVSQARCKFMHVHMVSNMAKYAARLSLILSKTIKLQVDLDSVTIERIEDI 400
Schil_LA0130_RDR   TKTASLKCYFVRFESIGTCDDGESYVFSTKTISQARCKFMHVHMVSNMAKYAARLSLILSKTIKLQVDLDSVTIERIEDI 400
Schil_LA1960_RDR   TKTASLKCYFVRFESIGTCDDGESYVFSTKTVSQARCKFMHVHMVSNMAKYAARLSLILSKTIKLQVDLDSVTIERIEDI 400
Schil_LA2737_RDR   TKTASLKCYFVRFESIGTCDDGESYVFSTKTVSQARCKFMHVHMVSNMAKYAARLSLILSKTIKLQVDLDSVTIERIEDI 400

Slyc_MM_RDR        LCRDENGCIIQDEDGEPRIHTDGTGFISEDLAMHCPKDFSKAEYIKDENYENFVDIVDLDDVNVERRVSVSRNRKPPLLM 476
Schil_Ty1_MV       LCRDENGCIIQDEDGEPRIHTDGTGFISEDLAMHCPKDFSKAEYIKDENYENFVDIVDLDDVNVERRASVSGNREPPLLM 480
Schil_Ty3_MV       LCRDENGCIIQDEDGEPRIHTDGTGFISEDLAMHCPKDFSKAEYIKDENYENFVDIVDLDDVNVERRASVSGNREPPLLM 480
Schil_LA1932_RDR   LCRDENGCIIQDEDGEPRIHTDGTGFISEDLAMHCPKDFSKAEYIKDENYENFVDIVDLDDVNVERRASVSGKREPPLLM 480
Schil_LA1938_RDR   LCRDENGCIIQDEDGEPRIHTDGTGFISEDLAMHCPKDFSKAEYIKDENYENFVDIVDLDDVNVERRASVSGNREPPLLM 480
Schil_LA1971_RDR   LCQDENGCIIQDEDGEPRIHTDGTGFISEDLAMHCPKDFSKAEYIKDENYENFVDIVDLDDVNVERRASVSGNREPPLLM 480
Schil_LA0130_RDR   LCRDENGCIIQDEDGEPRIHTDGTGFISEDLAMHCPKDFSKAEYIKDENYENFVDIVDLDDVNVERRASVSGNREPPLLM 480
Schil_LA1960_RDR   LCQDENGCIIQDEDGEPRIHTDGTGFISEDLAMHCPKDFSKAEYIKDENYENFVDIVDLDDVNVERRASVSGNREPPLLM 480
Schil_LA2737_RDR   LCRDENGCIIQDEDGEPRIHTDGTGFISEDLAMHCPKDFSKAEYIKDENYENFVDIVDLDDVNVERRASVSGNREPPLLM 480
```

```
Slyc_MM_RDR        QCRLFFNGCAVKGTFLVNRKIGSRKIHIRPSMVKVEIDPTISSIPTFDSLEIVAISHRPNKAYLSKNLISLLSYGGVHKE 556
Schil_Ty1_MV       QCRLFFNGCAVKGTFLVNRKIGSRKIHIRPSMVKVEIDPTISSIPTFDSLEIVAISHRPNKAYLSKNLISLLSYGGVHKE 560
Schil_Ty3_MV       QCRLFFNGCAVKGTFLVNRKIGSRKIHIRPSMVKVEIDPTISSIPTFDSLEIVAISHRPNKAYLSKNLISLLSYGGVHKE 560
Schil_LA1932_RDR   QCRLFFKGCAVKGTFLINRKIGSRKIHIRPSMVKVEIDPTISSIPTFDSLEIVAISHRPNKAYLSKNLISLLSYGGVHKE 560
Schil_LA1938_RDR   QCRLFFNGCAVKGTFLVNRKIGSRKIHIRPSMVKVEIDPTISSIPTFDSLEIVAISHRPNKAYLSKNLISLLSYGGVHKE 560
Schil_LA1971_RDR   QCRLFFNGCAVKGTFLVNRKIGSRKIHIRPSMVKVEIDPTISSIPTFDSLEIVAISHRPNKAYLSKNLISLLSYGGVHKE 560
Schil_LA0130_RDR   QCRLFFNGCAVKGTFLVNRKIGSRKIHIRPSMVKVEIDPTISSIPTFDSLEIVAISHRPNKAYLSKNLISLLSYGGVHKE 560
Schil_LA1960_RDR   QCRLFFNGCAVKGTFLVNRKIGSRKIHIRPSMVKVEIDPTISSIPTFDSLEIVAISHRPNKAYLSKNLISLLSYGGVHKE 560
Schil_LA2737_RDR   QCRLFFNGCAVKGTFLVNRKIGSRKIHIRPSMVKVEIDPTISSIPTFDSLEIVAISHRPNKAYLSKNLISLLSYGGVHKE 560

Slyc_MM_RDR        YFMELLGSALEETKQVYLRKRAALKVAINYREMDDECLTARMISSGIPLNEPHLHARLSRLAKIERTKLRGGKLPISDSF 636
Schil_Ty1_MV       YFLELLGSALEETKQVYLRKRAALKVAINYREMDDECLTARMISSGIPLNEPHLHVRLSRLAKIERTKLRGGKLPISDSF 640
Schil_Ty3_MV       YFLELLGSALEETKQVYLRKRAALKVAINYREMDDECLTARMISSGIPLNEPHLHVRLSRLAKIERTKLRGGKLPISDSF 640
Schil_LA1932_RDR   YFLELLGSALEETKQVYLRKRAALKVAINYREMDDECLTARMISSGIPLNEPHLHVRLSRLAKIERTKLRGGKLPISDSF 640
Schil_LA1938_RDR   YFLELLGSALEETKQVYLRKRAALKVAINYREMDDECLTARMISSGIPLNEPHLHVRLSRLAKIERTKLRGGKLPISDSF 640
Schil_LA1971_RDR   YFLELLGSALEETKQVYLRKRAALKVAINYREMDDECLTARMISSGIPLNEPHLHVRLSRLAKIERTKLRGGKLPISDSF 640
Schil_LA0130_RDR   YFLELLGSALEETKQVYLRKRAALKVAINYREMDDECLTARMISSGIPLNEPHLHVRLSRLAKIERTKLRGGKLPISDSF 640
Schil_LA1960_RDR   YFLELLGSALEETKQVYLRKRAALKVAINYREMDDECLTARMISSGIPLNEPHLHVRLSRLAKIERTKLRGGKLPISDSF 640
Schil_LA2737_RDR   YFLELLGSALEETKQVYLRKRAALKVAINYREMDDECLTARMISSGIPLNEPHLHVRLSRLAKIERTKLRGGKLPISDSF 640

Slyc_MM_RDR        YLMGTADPTGVLESNEVCVILDNGQVSGRVLVYRNPGLHFGDVHVMKARYVEELADVVGDAKYGIFFSTKGPRSAATEIA 716
Schil_Ty1_MV       YLMGTADPTGVLESNEVCVILDNGQVSGRVLVYRNPGLHFGDVHVMKARYVEELADVVGDAKYGIFFSTKGPRSAATEIA 720
Schil_Ty3_MV       YLMGTADPTGVLESNEVCVILDNGQVSGRVLVYRNPGLHFGDVHVMKARYVEELADVVGDAKYGIFFSTKGPRSAATEIA 720
Schil_LA1932_RDR   YLMGTADPTGVLESNEVCVILDNGQVSGRVLVYRNPGLHFGDVHVMKARYVEELADVVGDAKYGIFFSTKGPRSAATEIA 720
Schil_LA1938_RDR   YLMGTADPTGVLESNEVCVILDNGQVSGRVLVYRNPGLHFGDVHVMKARYVEELADVVGDAKYGIFFSTKGPRSAATEIA 720
Schil_LA1971_RDR   YLMGTADPTGVLESNEVCVILDNGQVSGRVLVYRNPGLHFGDVHVMKARYVEELADVVGDAKYGIFFSTKGPRSAATEIA 720
Schil_LA0130_RDR   YLMGTADPTGVLESNEVCVILDNGQVSGRVLVYRNPGLHFGDVHVMKARYVEELADVVGDAKYGIFFSTKGPRSAATEIA 720
Schil_LA1960_RDR   YLMGTADPTGVLESNEVCVILDNGQVSGRVLVYRNPGLHFGDVHVMKARYVEELADVVGDAKYGIFFSTKGPRSAATEIA 720
Schil_LA2737_RDR   YLMGTADPTGVLESNEVCVILDNGQVSGRVLVYRNPGLHFGDVHVMKARYVEELADVVGDAKYGIFFSTKGPRSAATEIA 720
```

```
Slyc_MM_RDR        NGDFDGDMYWVSINRKLVDSYTTSRPWIRMHSTPNAVSKKPSEFSADELEYELFRQFLEAKSKGANMSLAADSWLAFMDR 796
Schil_Ty1_MV       NGDFDGDMYWVSINRKLVDSYTTSRPWIRMHSTPKAVSKKPSEFSADELEYELFRQFLEAKSKGANMSLAADSWLAFMDR 800
Schil_Ty3_MV       NGDFDGDMYWVSINRKLVDSYTTSRPWIRMHSTPKAVSKKPSEFSADELEYELFRQFLEAKSKGANMSLAADSWLAFMDR 800
Schil_LA1932_RDR   NGDFDGDMYWVSINRKLVDSYTTSRPWIRIHSTPKAVSKKPSEFSADELEYELFRQFLEAKSKGANMSLAADSWLAFMDR 800
Schil_LA1938_RDR   NGDFDGDMYWVSINRKLVDSYTTSRPWIRMHSTPKAVSKKPSEFSADELEYELFRQFLEAKSKGANMSLAADSWLAFMDR 800
Schil_LA1971_RDR   NGDFDGDMYWVSINRKLVDSYTTSRPWIRMHSTPKAVSKKPSEFSADELEYELFRQFLEAKSKGANMSLAADSWLAFMDR 800
Schil_LA0130_RDR   NGDFDGDMYWVSINRKLVDSYTTSRPWIRMHSTPKAVSKKPSEFSADELEYELFRQFLEAKSKGANMSLAADSWLAFMDR 800
Schil_LA1960_RDR   NGDFDGDMYWVSINRKLVDSYTTSRPWIRMHSTPKAVSKKPSEFSADELEYELFRQFLEAKSKGANMSLAADSWLAFMDR 800
Schil_LA2737_RDR   NGDFDGDMYWVSINRKLVDSYTTSRPWIRMHSTPKAVSKKPSEFSADELEYELFRQFLEAKSKGANMSLAADSWLAFMDR 800

Slyc_MM_RDR        LLTLRDDNVDDMHSLKGKMLHLIDIYYDALDAPKSGKKVSIPHYLKANKFPHYMEKGNSCSYHSTSILGQIYDHVDSYPD 876
Schil_Ty1_MV       LLMLRDDNVDDMHSLKGKMLHLIDIYYDALDAPKSGKKVSIPHYLKANKFPHYMEKGNSCSYHSTSILGQIYDHVDSYPD 880
Schil_Ty3_MV       LLMLRDDNVDDMHSLKGKMLHLIDIYYDALDAPKSGKKVSIPHYLKANKFPHYMEKGNSCSYHSTSILGQIYDHVDSYPD 880
Schil_LA1932_RDR   LLMLRDDNVDDMHSLKGKMLHLIDIYYDALDAPKSGKKVSIPHYLKANKFPHYMEKGNSCSYHSTSILGQIYDHVDSYPD 880
Schil_LA1938_RDR   LLMLRDDNVDDMHSLKGKMLHLIDIYYDALDAPKSGKKVSIPHYLKANKFPHYMEKGNSCSYHSTSILGQIYDHVDSYPD 880
Schil_LA1971_RDR   LLMLRDDNVDDMHSLKGKMLHLIDIYYDALDAPKSGKKVSIPHYLKANKFPHYMEKGNSCSYHSTSILGQIYDHVDSYPD 880
Schil_LA0130_RDR   LLMLQDDNVDDMHSLKGKMLHLIDIYYDALDAPKSGKKVSIPHYLKANKFPHYMEKGNSCSYHSTSILGQIYDHVDSYPD 880
Schil_LA1960_RDR   LLMLRDDNVDDMHSLKGKMLHLIDIYYDALDAPKSGKKVSIPHYLKANKFPHYMEKGNSCSYHSTSILGQIYDHVDSYPD 880
Schil_LA2737_RDR   LLMLRDDNVDDMHSLKGKMLHLIDIYYDALDAPKSGKKVSIPHYLKANKFPHYMEKGNSCSYHSTSILGQIYDHVDSYPD 880

Slyc_MM_RDR        EDLCITEISKLPCFEVEIPQRCMTLWRGRYEEYKKDMTRAMNFDCELRITSCNEVIKKYKMLLYGAVEFEQTVRKTEDIF 956
Schil_Ty1_MV       EDLCITEISKLPCFEVEIPQRCMTLWRGRYEEYKKDMTQAMNLDCELRITSCNEVIKKYKMLLYGAVEFEQTVRKTEDIF 960
Schil_Ty3_MV       EDLCITEISKLPCFEVEIPQRCMTLWRGRYEEYKKDMTQAMNLDCELRITSCNEVIKKYKMLLYGAVEFEQTVRKTEDIF 960
Schil_LA1932_RDR   EDLCITEISKLPCFEVEIPQRCMTLWRGRYEEYKKDMTQAMNLDCELRITSCNEVIKKYKMLLYGAVEFEQTVRKTEDIF 960
Schil_LA1938_RDR   EDLCITEISKLPCFEVEIPQRCMTLWRGRYEEYKKDMTQAMNLDCELRITSCNEVIKKYKMLLYGAVEFEQTVRKTEDIF 960
Schil_LA1971_RDR   EDLCITEISKLPCFEVEIPQRCMTLWRGRYEEYKKDMTQAMNLDCELRITSCNEVIKKYKMLLYGAVEFEQTVRKTEDIF 960
Schil_LA0130_RDR   EDLCITEISKLPCFEVEIPQRCMTLWRGRYEKYKKDMTQAMNLDCELRITSCNEVIKKYKMLLYGAVEFEQTVRKTEDIF 960
Schil_LA1960_RDR   EDLCITEISKLPCFEVEIPQRCMTLWRGRYEEYKKDMTQAMNLDCELRITSCNEVIKKYKMLLYGAVEFEQTVRKTEDIF 960
Schil_LA2737_RDR   EDLCITEISKLPCFEVEIPQRCMTLWRGRYEEYKKDMTQAMNLDCELRITSCNEVIKKYKMLLYGAVEFEQTVRKTEDIF 960
```

```
Slyc_MM_RDR          DEALAIYHVTYDNARITYSIEKCGFAWKVAGSALCRIHAMYRKEKDLPILPSVLQEIL  1014
Schil_Ty1_MV         DEALAIYHVTYDNARITYSIEKCGFAWKVAGSALCRIHAMYRKEKDLPILPSVLQEIL  1018
Schil_Ty3_MV         DEALAIYHVTYDNARITYSIEKCGFAWKVAGSALCRIHAMYRKEKDLPILPSVLQEIL  1018
Schil_LA1932_RDR     DEALAIYHVTYDNARITYSIEKCGFAWKVAGSALCRIHAMYHKEKDLPILPSVLQEIL  1018
Schil_LA1938_RDR     DEALAIYHVTYDNARITYSIEKCGFAWKVAGSALCRIHAMYRKEKDLPILPSVLQEIL  1018
Schil_LA1971_RDR     DEALAIYHVTYDNARIAYSIEKCGFAWKVAGSALCRIHAMYRKEKDLPILPSVLQEIL  1018
Schil_LA0130_RDR     DEALAIYHVTYDNARITYSIEKCGFAWKVAGSALCRIHAMYRKEKDLPILPSVLQEIL  1019
Schil_LA1960_RDR     DEALAIYHVTYDNARITYSIEKCGFAWKVAGSALCRIHAMYRKEKDLPILPSVLQEIL  1019
Schil_LA2737_RDR     DEALAIYHVTYDNARITYSIEKCGFAWKVAGSALCRIHAMYRKEKDLPILPSVLQEIL  1019
```
