# Supplementary material for: Resistance to Tomato Yellow Leaf Curl Virus in Tomato Germplasm
Source: Front Plant Sci. 2018 Aug 20;9:1198. doi: 10.3389/fpls.2018.01198 (PMC6110163; doi:10.3389/fpls.2018.01198)
Supplement: TABLE S1 — Summary of responses of wild tomato species upon natural infection with tomato yellow leaf curl disease at the Institute of Vegetables and Flowers, Chinese Academy of Agricultural Sciences, Beijing. [file Table_1.docx]

**Supplementary Table S1.** Summary of responses of wild tomato species upon natural infection with Tomato Yellow Leaf Curl Disease at the Institute of Vegetables and Flowers, Chinese Academy of Agricultural Sciences, Beijing.

| **Wild species ^a^** | **Equivalent *Lycopersicon* name** | **Number of Accessions ^b^** | | | |
| --- | --- | --- | --- | --- | --- |
|  |  | **Total** | **Symptomless** | **Symptomatic** | **Segregating** |
| *S. arcanum* | *L. peruvianum* / *L. peruvianum var. humifusum* | 24 | 14 | 5 | 5 |
| *S. cheesmaniae* | *L. cheesmanii* | 7 | 0 | 7 | 0 |
| *S. chilense* | *L. chilense* | 51 | 43 | 4 | 4 |
| *S. chmielewskii* | *L. chmielewskii* | 3 | 1 | 2 | 0 |
| *S. corneliomulleri* | *L. peruvianum* / *L. peruv. f. glandulosum* | 44 | 28 | 7 | 9 |
| *S. galapagense* | *L. cheesmanii f. minor* | 2 | 0 | 2 | 0 |
| *S. habrochaites* | *L. hirsutum* / *L. hirsutum f. glabratum* | 36 | 2 | 22 | 12 |
| *S. huaylasense* | *L. peruvianum* | 4 | 4 | 0 | 0 |
| *S. lycopersicoides* | *L. lycopersicoides* | 2 | 0 | 2 | 0 |
| *S. neorickii* | *L. parviflorum* | 3 | 1 | 2 | 0 |
| *S. pennellii* | *L. pennellii* / *L. pennellii var. puberulum* | 38 | 1 | 35 | 2 |
| *S. peruvianum* | *L. peruvianum* | 81 | 39 | 22 | 20 |
| *S. pimpinellifolium* | *L. pimpinellifolium* | 406 | 0 | 406 | 0 |

^a^ Taxon using the classification system of (Peralta et al. 2008)

^b^ Symptomless accessions were those for which all the tested plants of each accession were free of symptoms. Symptomatic accession were those for which all the tested individuals exhibited TYLCV symptoms, although symptoms may have varied in severity from plant to plant. Segregating accessions showed heterogeneous phenotypes with both symptomatic and symptomless plants for the same accession
