# Supplementary material for: Resistance to Tomato Yellow Leaf Curl Virus in Tomato Germplasm
Source: Front Plant Sci. 2018 Aug 20;9:1198. doi: 10.3389/fpls.2018.01198 (PMC6110163; doi:10.3389/fpls.2018.01198)
Supplement: TABLE S2 — Average disease severity index ratings of Solanum spp. accessions upon natural infection with tomato yellow leaf curl disease at the Institute of Vegetables and Flowers, Chinese Academy of Agricultural Sciences, Beijing. [file Table_2.docx]

**Supplementary Table S2.** Average disease severity index ratings of *Solanum* spp. accessions upon natural infection with Tomato Yellow Leaf Curl Disease at the Institute of Vegetables and Flowers, Chinese Academy of Agricultural Sciences, Beijing.

| ***Solanum* spp. accession^a^** | **Source^b^** | **Average**  **DSI^c^** | **Phenotype^d^** |
| --- | --- | --- | --- |
| *Solanum arcanum* |  |  |  |
| LA 0378 | AVRDC | 0 ± 0 | symptomless |
| LA 0385 | CGN | 0 ± 0 | symptomless |
| LA 0441 | AVRDC | 0 ± 0 | symptomless |
| LA 1626 | TGRC | 0 ± 0 | symptomless |
| LA 1984 | AVRDC | 0 ± 0 | symptomless |
| LA 2152 | TGRC | 0 ± 0 | symptomless |
| LA 2172 ^e^ | TGRC | 0 ± 0 | symptomless |
| LA 2326 | CGN | 0 ± 0 | symptomless |
| LA 2333 | CGN | 0 ± 0 | symptomless |
| LA 2388 | TGRC | 0 ± 0 | symptomless |
| LA 2548 | TGRC | 0 ± 0 | symptomless |
| LA 2553 | AVRDC | 0 ± 0 | symptomless |
| LA 2582 | TGRC | 0 ± 0 | symptomless |
| LA 2917 | TGRC | 0 ± 0 | symptomless |
| LA 1351 | TGRC | 1 ± 0 | symptomatic |
| LA 1395 | TGRC | 1 ± 0 | symptomatic |
| LA 2150 | TGRC | 1 ± 0 | symptomatic |
| LA 2153 | TGRC | 1 ± 0 | symptomatic |
| LA 1350 | TGRC | 3 ± 0 | symptomatic |
| *Solanum cheesmaniae* |  |  |  |
| LA 0166 | CGN | 3 ± 0 | symptomatic |
| LA 0422 | TGRC | 3 ± 0 | symptomatic |
| LA 0429 | TGRC | 3.1 ± 0.9 | symptomatic |
| LA 1448 | CGN | 3.4 ± 0.4 | symptomatic |
| LA 1409 | CGN | 3.9 ± 0.2 | symptomatic |
| LA 0932 | CGN | 4 ± 0 | symptomatic |
| LA 1402 | CGN | 4 ± 0 | symptomatic |
| *Solanum chilense* |  |  |  |
| LA 0130 | TGRC | 0 ± 0 | symptomless |
| LA 0294 | TGRC | 0 ± 0 | symptomless |
| LA 0456 | TGRC | 0 ± 0 | symptomless |
| LA 0458 | TGRC | 0 ± 0 | symptomless |
| LA 0460 | TGRC | 0 ± 0 | symptomless |
| LA 1030 | TGRC | 0 ± 0 | symptomless |
| LA 1917 | TGRC | 0 ± 0 | symptomless |
| LA 1930 | TGRC | 0 ± 0 | symptomless |
| LA 1938 | TGRC | 0 ± 0 | symptomless |
| LA 1958 | TGRC | 0 ± 0 | symptomless |
| LA 1959 | TGRC | 0 ± 0 | symptomless |
| LA 1960 | TGRC | 0 ± 0 | symptomless |
| LA 1963 | AVRDC | 0 ± 0 | symptomless |
| LA 1965 | TGRC | 0 ± 0 | symptomless |
| (*Continued on next page*) | | | |

**Supplementary Table S2.** Average disease severity index ratings of *Solanum* spp. accessions upon natural infection with Tomato Yellow Leaf Curl Disease at the Institute of Vegetables and Flowers, Chinese Academy of Agricultural Sciences, Beijing.

| ***Solanum* spp. accession^a^** | **Source^b^** | **Average**  **DSI^c^** | **Phenotype^d^** |
| --- | --- | --- | --- |
| *Solanum chilense* |  |  |  |
| LA 1967 | TGRC | 0 ± 0 | symptomless |
| LA 1968 | TGRC | 0 ± 0 | symptomless |
| LA 1970 | TGRC | 0 ± 0 | symptomless |
| LA 1971 | AVRDC | 0 ± 0 | symptomless |
| LA 1972 | TGRC | 0 ± 0 | symptomless |
| LA 2404 | TGRC | 0 ± 0 | symptomless |
| LA 2405 | TGRC | 0 ± 0 | symptomless |
| LA 2406 | TGRC | 0 ± 0 | symptomless |
| LA 2731 | TGRC | 0 ± 0 | symptomless |
| LA 2737 | TGRC | 0 ± 0 | symptomless |
| LA 2739 | TGRC | 0 ± 0 | symptomless |
| LA 2746 | TGRC | 0 ± 0 | symptomless |
| LA 2747 | TGRC | 0 ± 0 | symptomless |
| LA 2748 | TGRC | 0 ± 0 | symptomless |
| LA 2749 | TGRC | 0 ± 0 | symptomless |
| LA 2750 | TGRC | 0 ± 0 | symptomless |
| LA 2751 | TGRC | 0 ± 0 | symptomless |
| LA 2753 | TGRC | 0 ± 0 | symptomless |
| LA 2762 | TGRC | 0 ± 0 | symptomless |
| LA 2764 | TGRC | 0 ± 0 | symptomless |
| LA 2765 | TGRC | 0 ± 0 | symptomless |
| LA 2768 | TGRC | 0 ± 0 | symptomless |
| LA 2930 | TGRC | 0 ± 0 | symptomless |
| LA 2931 | AVRDC | 0 ± 0 | symptomless |
| LA 2932 | TGRC | 0 ± 0 | symptomless |
| VI031797 | AVRDC | 0 ± 0 | symptomless |
| VI031802 | AVRDC | 0 ± 0 | symptomless |
| VI031803 | AVRDC | 0 ± 0 | symptomless |
| VI046773 | AVRDC | 0 ± 0 | symptomless |
| LA 4108 | TGRC | 1 ± 0 | symptomatic |
| LA 4330 | TGRC | 1 ± 0 | symptomatic |
| LA 4332 | TGRC | 1 ± 0 | symptomatic |
| LA 4109 | TGRC | 2 ± 0 | symptomatic |
| *Solanum chmielewskii* |  |  |  |
| CGN19145 | CGN | 0 ± 0 | symptomless |
| LA 1045 | CGN | 2.3 ± 0.3 | symptomatic |
| LA 1028 | TGRC | 2.8 ± 1 | symptomatic |
| *Solanum corneliomulleri* |  |  |  |
| CGN14358 | CGN | 0 ± 0 | symptomless |
| CGN15303 | CGN | 0 ± 0 | symptomless |
| CGN15802 | CGN | 0 ± 0 | symptomless |
| CGN15803 | CGN | 0 ± 0 | symptomless |
| (*Continued on next page*) | | | |

**Supplementary Table S2.** Average disease severity index ratings of *Solanum* spp. accessions upon natural infection with Tomato Yellow Leaf Curl Disease at the Institute of Vegetables and Flowers, Chinese Academy of Agricultural Sciences, Beijing.

| ***Solanum* spp. accession^a^** | **Source^b^** | **Average**  **DSI^c^** | **Phenotype^d^** |
| --- | --- | --- | --- |
| *Solanum corneliomulleri* |  |  |  |
| LA 0107 | TGRC | 0 ± 0 | symptomless |
| LA 0366 | AVRDC | 0 ± 0 | symptomless |
| LA 0444 | AVRDC | 0 ± 0 | symptomless |
| LA 0451 | TGRC | 0 ± 0 | symptomless |
| LA 1271 | TGRC | 0 ± 0 | symptomless |
| LA 1274 | TGRC | 0 ± 0 | symptomless |
| LA 1281 | TGRC | 0 ± 0 | symptomless |
| LA 1283 | TGRC | 0 ± 0 | symptomless |
| LA 1292 | TGRC | 0 ± 0 | symptomless |
| LA 1293 | TGRC | 0 ± 0 | symptomless |
| LA 1296 | TGRC | 0 ± 0 | symptomless |
| LA 1305 | AVRDC | 0 ± 0 | symptomless |
| LA 1331 | TGRC | 0 ± 0 | symptomless |
| LA 1373 | CGN | 0 ± 0 | symptomless |
| LA 1473 | TGRC | 0 ± 0 | symptomless |
| LA 1552 | TGRC | 0 ± 0 | symptomless |
| LA 1609 | TGRC | 0 ± 0 | symptomless |
| LA 1647 | TGRC | 0 ± 0 | symptomless |
| LA 1653 | TGRC | 0 ± 0 | symptomless |
| LA 1723 | TGRC | 0 ± 0 | symptomless |
| LA 1910 | TGRC | 0 ± 0 | symptomless |
| LA 1945 | TGRC | 0 ± 0 | symptomless |
| LA 1973 | TGRC | 0 ± 0 | symptomless |
| LA 3156 | TGRC | 0 ± 0 | symptomless |
| LA 1339 | TGRC | 1 ± 0 | symptomatic |
| LA 1722 | TGRC | 1 ± 0 | symptomatic |
| LA 1937 | TGRC | 1 ± 0 | symptomatic |
| CGN15793 | CGN | 1.3 ± 0.6 | symptomatic |
| LA 1377 | TGRC | 2 ± 0 | symptomatic |
| LA 0103 | TGRC | 2.5 ± 2.1 | symptomatic |
| PI 199380 | AVRDC | 3.9 ± 0.4 | symptomatic |
| *Solanum galapagense* |  |  |  |
| LA 1400 | TGRC | 2.5 ± 0.5 | symptomatic |
| LA 1411 | TGRC | 3 ± 1 | symptomatic |
| *Solanum habrochaites* |  |  |  |
| H302 | KSU | 0 ± 0 | symptomless |
| LA 2314 | TGRC | 0 ± 0 | symptomless |
| PI 128644 | AVRDC | 1 ± 0.5 | symptomatic |
| H30506 | KSU | 1.5 ± 1.2 | symptomatic |
| 4M03 | KSU | 2 ± 0.7 | symptomatic |
| M202 | KSU | 2 ± 0.9 | symptomatic |
| 2H03 | KSU | 2 ± 1.4 | symptomatic |
| (*Continued on next page*) | | | |

**Supplementary Table S2.** Average disease severity index ratings of *Solanum* spp. accessions upon natural infection with Tomato Yellow Leaf Curl Disease at the Institute of Vegetables and Flowers, Chinese Academy of Agricultural Sciences, Beijing.

| ***Solanum* spp. accession^a^** | **Source^b^** | **Average**  **DSI^c^** | **Phenotype^d^** |
| --- | --- | --- | --- |
| *Solanum habrochaites* |  |  |  |
| CGN15817 | CGN | 2.1 ± 0.3 | symptomatic |
| CGN15914 | CGN | 2.3 ± 0.2 | symptomatic |
| M20303 | KSU | 2.3 ± 1 | symptomatic |
| PI 134417 | AVRDC | 2.4 ± 1.3 | symptomatic |
| M203 | KSU | 2.5 ± 0.7 | symptomatic |
| LA 1223 | TGRC | 2.5 ± 0.8 | symptomatic |
| CGN15878 | CGN | 2.8 ± 0.3 | symptomatic |
| M20202 | KSU | 2.8 ± 0.5 | symptomatic |
| CGN15428 | CGN | 3 ± 0 | symptomatic |
| CGN15790 | CGN | 3 ± 0 | symptomatic |
| CGN15370 | CGN | 3 ± 0.1 | symptomatic |
| CGN15391 | CGN | 3 ± 0.2 | symptomatic |
| CGN15879 | CGN | 3 ± 1 | symptomatic |
| CGN15792 ^e^ | CGN | 3.1 ± 0.3 | symptomatic |
| CGN15791 ^e^ | CGN | 3.5 ± 0.1 | symptomatic |
| CGN24036 | CGN | 3.6 ± 0.4 | symptomatic |
| CGN24035 | CGN | 3.8 ± 0.3 | symptomatic |
| PI 308182 | CGN | 4 ± 0.2 | symptomatic |
| *Solanum huaylasense* |  |  |  |
| LA 1360 | TGRC | 0 ± 0 | symptomless |
| LA 1364 ^e^ | AVRDC | 0 ± 0 | symptomless |
| LA 1365 ^e^ | AVRDC | 0 ± 0 | symptomless |
| LA 1982 | AVRDC | 0 ± 0 | symptomless |
| *Solanum lycopersicoides* |  |  |  |
| LA 2408 | TGRC | 3 ± 0 | symptomatic |
| CGN23973 | CGN | 3 ± 0.2 | symptomatic |
| *Solanum neorickii* |  |  |  |
| LA 1716 | AVRDC | 0 ± 0 | symptomless |
| CGN15816 | CGN | 2.3 ± 0.4 | symptomatic |
| LA 1321 | AVRDC | 3.1 ± 0.4 | symptomatic |
| *Solanum pennellii* |  |  |  |
| LA 1733 | TGRC | 0 ± 0 | symptomless |
| LA 1303 | TGRC | 1 ± 0 | symptomatic |
| LA 1656 | TGRC | 1 ± 0 | symptomatic |
| LA 1674 | TGRC | 1 ± 0 | symptomatic |
| LA 1299 | TGRC | 1.5 ± 0.8 | symptomatic |
| LA 1277 | TGRC | 1.6 ± 1.1 | symptomatic |
| LA 1273 | TGRC | 1.8 ± 0.8 | symptomatic |
| LA 1809 | TGRC | 1.9 ± 0.2 | symptomatic |
| PI 365976 | CGN | 1.9 ± 1 | symptomatic |
| LA 0716 ^e^ | TGRC | 2 ± 0 | symptomatic |
| LA 1297 | TGRC | 2 ± 0 | symptomatic |
| (*Continued on next page*) | | | |

**Supplementary Table S2.** Average disease severity index ratings of *Solanum* spp. accessions upon natural infection with Tomato Yellow Leaf Curl Disease at the Institute of Vegetables and Flowers, Chinese Academy of Agricultural Sciences, Beijing.

| ***Solanum* spp. accession^a^** | **Source^b^** | **Average**  **DSI^c^** | **Phenotype^d^** |
| --- | --- | --- | --- |
| *Solanum pennellii* |  |  |  |
| LA 1724 | TGRC | 2 ± 0 | symptomatic |
| LA 2580 | TGRC | 2 ± 0 | symptomatic |
| PI 365977 | CGN | 2 ± 0 | symptomatic |
| PI 365970 | CGN | 2 ± 0.4 | symptomatic |
| LA 1946 | TGRC | 2 ± 1.4 | symptomatic |
| LA 1275 | TGRC | 2.1 ± 0.7 | symptomatic |
| LA 1693 | TGRC | 2.2 ± 0.3 | symptomatic |
| LA 0751 | TGRC | 2.4 ± 0.4 | symptomatic |
| LA 1943 | TGRC | 2.5 ± 0.7 | symptomatic |
| LA 1912 | TGRC | 2.6 ± 0.3 | symptomatic |
| CGN24194 | CGN | 2.6 ± 0.4 | symptomatic |
| LA 1272 ^e^ | TGRC | 2.6 ± 0.4 | symptomatic |
| LA 1734 | TGRC | 2.8 ± 0.9 | symptomatic |
| H303 | KSU | 2.8 ± 1.5 | symptomatic |
| LA 0750 | CGN | 3 ± 0 | symptomatic |
| LA 1302 | TGRC | 3 ± 0 | symptomatic |
| LA 1356 | TGRC | 3 ± 0 | symptomatic |
| LA 1657 | TGRC | 3 ± 0 | symptomatic |
| LA 1911 | TGRC | 3 ± 0 | symptomatic |
| LA 2657 | TGRC | 3 ± 0 | symptomatic |
| CGN15818 | CGN | 3.4 ± 0.7 | symptomatic |
| LA 1926 | TGRC | 3.8 ± 0.3 | symptomatic |
| LA 1649 | TGRC | 4 ± 0 | symptomatic |
| LA 2963 | TGRC | 4 ± 0 | symptomatic |
| *Solanum peruvianum* |  |  |  |
| CGN14501 | CGN | 0 ± 0 | symptomless |
| CGN15306 | CGN | 0 ± 0 | symptomless |
| CGN15530 ^e^ | CGN | 0 ± 0 | symptomless |
| CGN15532 ^e^ | CGN | 0 ± 0 | symptomless |
| CGN15794 | CGN | 0 ± 0 | symptomless |
| CGN23955 | CGN | 0 ± 0 | symptomless |
| LA 0372 | CGN | 0 ± 0 | symptomless |
| LA 0374 | CGN | 0 ± 0 | symptomless |
| LA 0445 | TGRC | 0 ± 0 | symptomless |
| LA 0446 | TGRC | 0 ± 0 | symptomless |
| LA 0448 | TGRC | 0 ± 0 | symptomless |
| LA 0455 | TGRC | 0 ± 0 | symptomless |
| LA 1336 | TGRC | 0 ± 0 | symptomless |
| LA 1368 | TGRC | 0 ± 0 | symptomless |
| LA 1513 | TGRC | 0 ± 0 | symptomless |
| LA 1517 | TGRC | 0 ± 0 | symptomless |
| (*Continued on next page*) | | | |

**Supplementary Table S2.** Average disease severity index ratings of *Solanum* spp. accessions upon natural infection with Tomato Yellow Leaf Curl Disease at the Institute of Vegetables and Flowers, Chinese Academy of Agricultural Sciences, Beijing.

| ***Solanum* spp. accession^a^** | **Source^b^** | **Average**  **DSI^c^** | **Phenotype^d^** |
| --- | --- | --- | --- |
| *Solanum peruvianum* |  |  |  |
| LA 1537 | TGRC | 0 ± 0 | symptomless |
| LA 1929 | TGRC | 0 ± 0 | symptomless |
| LA 1935 | TGRC | 0 ± 0 | symptomless |
| LA 1977 | TGRC | 0 ± 0 | symptomless |
| LA 2732 | AVRDC | 0 ± 0 | symptomless |
| LA 2964 | TGRC | 0 ± 0 | symptomless |
| LA 3790 | TGRC | 0 ± 0 | symptomless |
| LA 4125 | TGRC | 0 ± 0 | symptomless |
| LA 4317 | TGRC | 0 ± 0 | symptomless |
| LA 4318 | TGRC | 0 ± 0 | symptomless |
| LA 4445 | TGRC | 0 ± 0 | symptomless |
| PI 126928 | AVRDC | 0 ± 0 | symptomless |
| PI 128643 | AVRDC | 0 ± 0 | symptomless |
| PI 128647 | AVRDC | 0 ± 0 | symptomless |
| PI 128650-6Y-IV-1 | CGN | 0 ± 0 | symptomless |
| PI 128651 | AVRDC | 0 ± 0 | symptomless |
| PI 128653 | CGN | 0 ± 0 | symptomless |
| PI 128656 | AVRDC | 0 ± 0 | symptomless |
| PI 128657 | AVRDC | 0 ± 0 | symptomless |
| PI 128663 | AVRDC | 0 ± 0 | symptomless |
| PI 251306 | AVRDC | 0 ± 0 | symptomless |
| PI 266375 ^e^ | AVRDC | 0 ± 0 | symptomless |
| LA 1692 | TGRC | 1 ± 0 | symptomatic |
| LA 1947 | TGRC | 1 ± 0 | symptomatic |
| LA 1949 | TGRC | 1 ± 0 | symptomatic |
| LA 1954 ^e^ | TGRC | 1 ± 0 | symptomatic |
| LA 1955 | TGRC | 1 ± 0 | symptomatic |
| LA 2581 | TGRC | 1 ± 0 | symptomatic |
| PI 126444 | AVRDC | 1 ± 0 | symptomatic |
| PI 126929 | AVRDC | 1 ± 0 | symptomatic |
| PI 126935 | AVRDC | 1 ± 0 | symptomatic |
| PI 126946 | AVRDC | 1 ± 0 | symptomatic |
| PI 128654 | AVRDC | 1 ± 0 | symptomatic |
| PI 128659 | AVRDC | 1 ± 0 | symptomatic |
| PI 128660 | AVRDC | 1 ± 0 | symptomatic |
| PI 129149 | AVRDC | 1 ± 0 | symptomatic |
| PI 129152 | AVRDC | 1 ± 0 | symptomatic |
| PI 143679 | AVRDC | 1 ± 0 | symptomatic |
| CGN15795 | CGN | 1.4 ± 0.4 | symptomatic |
| LA 0462 | TGRC | 2 ± 0 | symptomatic |
| PI 128652 | AVRDC | 2 ± 0 | symptomatic |
| PI 129145 | AVRDC | 2 ± 0 | symptomatic |
| (*Continued on next page*) | | | |

**Supplementary Table S2.** Average disease severity index ratings of *Solanum* spp. accessions upon natural infection with Tomato Yellow Leaf Curl Disease at the Institute of Vegetables and Flowers, Chinese Academy of Agricultural Sciences, Beijing.

| ***Solanum* spp. accession^a^** | **Source^b^** | **Average**  **DSI^c^** | **Phenotype^d^** |
| --- | --- | --- | --- |
| *Solanum peruvianum* |  |  |  |
| PI 251314 | AVRDC | 2.8 ± 0.5 | symptomatic |
| LA 3218 | TGRC | 4 ± 0 | symptomatic |
| *Solanum pimpinellifolium* |  |  |  |
| LA 1607 | TGRC | 0.5 ± 0.5 | symptomatic |
| LA 1344 | TGRC | 1 ± 0 | symptomatic |
| LA 2578 | TGRC | 1 ± 1.1 | symptomatic |
| LA 1863 | TGRC | 1.3 ± 0.4 | symptomatic |
| LA 0398 | TGRC | 1.6 ± 0.7 | symptomatic |
| LA 1630 | TGRC | 1.8 ± 0.5 | symptomatic |
| LA 1589 | TGRC | 1.8 ± 0.7 | symptomatic |
| LA 1597 | TGRC | 2 ± 0 | symptomatic |
| LA 1846 | TGRC | 2 ± 0 | symptomatic |
| LA 1605 | TGRC | 2 ± 0.6 | symptomatic |
| PI 211838 | AVRDC | 2 ± 0.8 | symptomatic |
| LA 1355 | TGRC | 2 ± 0.9 | symptomatic |
| LA 1742 | TGRC | 2.1 ± 0.2 | symptomatic |
| LA 1256 | TGRC | 2.1 ± 0.6 | symptomatic |
| PI 211840 | AVRDC | 2.1 ± 0.7 | symptomatic |
| LA 0122 | TGRC | 2.2 ± 0.3 | symptomatic |
| LA 1237 | TGRC | 2.2 ± 0.9 | symptomatic |
| LA 1817 | TGRC | 2.3 ± 0.6 | symptomatic |
| LA 1357 | TGRC | 2.3 ± 0.8 | symptomatic |
| CGN15302 | CGN | 2.4 ± 0.6 | symptomatic |
| LA 1579 | TGRC | 2.4 ± 0.6 | symptomatic |
| PI 143527 | AVRDC | 2.5 ± 0.4 | symptomatic |
| LA 1380 | AVRDC | 2.5 ± 0.6 | symptomatic |
| PI 390704 | AVRDC | 2.5 ± 0.6 | symptomatic |
| PI 143524 | AVRDC | 2.5 ± 0.7 | symptomatic |
| PI 211839 | AVRDC | 2.5 ± 0.7 | symptomatic |
| LA 1345 | TGRC | 2.5 ± 1 | symptomatic |
| LA 1374 | TGRC | 2.6 ± 0.4 | symptomatic |
| LA 1686 | TGRC | 2.6 ± 0.4 | symptomatic |
| LA 1581 | TGRC | 2.6 ± 0.5 | symptomatic |
| LA 1924 | TGRC | 2.6 ± 0.5 | symptomatic |
| PI 390696 | AVRDC | 2.6 ± 0.6 | symptomatic |
| PI 407546 | AVRDC | 2.6 ± 0.6 | symptomatic |
| PI 407544 | AVRDC | 2.6 ± 0.7 | symptomatic |
| LA 1561 | TGRC | 2.6 ± 0.8 | symptomatic |
| LA 2176 | TGRC | 2.6 ± 1.2 | symptomatic |
| LA 1583 | TGRC | 2.6 ± 1.4 | symptomatic |
| PI 126940 | AVRDC | 2.7 ± 0.3 | symptomatic |
| PI 390689 | AVRDC | 2.7 ± 0.3 | symptomatic |
| (*Continued on next page*) | | | |

**Supplementary Table S2.** Average disease severity index ratings of *Solanum* spp. accessions upon natural infection with Tomato Yellow Leaf Curl Disease at the Institute of Vegetables and Flowers, Chinese Academy of Agricultural Sciences, Beijing.

| ***Solanum* spp. accession^a^** | **Source^b^** | **Average**  **DSI^c^** | **Phenotype^d^** |
| --- | --- | --- | --- |
| *Solanum pimpinellifolium* |  |  |  |
| PI 126932 | AVRDC | 2.7 ± 0.4 | symptomatic |
| PI 407543 | AVRDC | 2.7 ± 0.4 | symptomatic |
| PI 390698 | AVRDC | 2.7 ± 0.5 | symptomatic |
| PI 126934 | AVRDC | 2.7 ± 0.6 | symptomatic |
| PI 407537 | AVRDC | 2.7 ± 0.6 | symptomatic |
| PI 407555 | AVRDC | 2.7 ± 0.6 | symptomatic |
| PI 407557 | AVRDC | 2.7 ± 0.7 | symptomatic |
| LA 1383 | TGRC | 2.7 ± 0.8 | symptomatic |
| LA 1861 | TGRC | 2.7 ± 0.8 | symptomatic |
| LA 2093 | TGRC | 2.7 ± 0.8 | symptomatic |
| LA 1580 | TGRC | 2.8 ± 0.3 | symptomatic |
| LA 2001 | TGRC | 2.8 ± 0.3 | symptomatic |
| PI 407536 | AVRDC | 2.8 ± 0.3 | symptomatic |
| LA 1370 | TGRC | 2.8 ± 0.4 | symptomatic |
| LA 2423 | TGRC | 2.8 ± 0.4 | symptomatic |
| LA 3161 | TGRC | 2.8 ± 0.4 | symptomatic |
| PI 390695 | AVRDC | 2.8 ± 0.4 | symptomatic |
| LA 1578 ^e^ | TGRC | 2.8 ± 0.5 | symptomatic |
| LA 2656 | AVRDC | 2.8 ± 0.5 | symptomatic |
| PI 390713 | AVRDC | 2.8 ± 0.5 | symptomatic |
| PI 407542 | AVRDC | 2.8 ± 0.6 | symptomatic |
| PI 407553 | AVRDC | 2.8 ± 0.7 | symptomatic |
| LA 0376 | TGRC | 2.8 ± 0.8 | symptomatic |
| LA 1603 | TGRC | 2.8 ± 0.9 | symptomatic |
| LA 1847 | TGRC | 2.8 ± 0.9 | symptomatic |
| LA 2647 | TGRC | 2.8 ± 1 | symptomatic |
| LA 2426 | TGRC | 2.8 ± 1.3 | symptomatic |
| PI 390694 | AVRDC | 2.9 ± 0.3 | symptomatic |
| PI 126937 | AVRDC | 2.9 ± 0.4 | symptomatic |
| VI030547 | AVRDC | 2.9 ± 0.4 | symptomatic |
| VI030548 | AVRDC | 2.9 ± 0.4 | symptomatic |
| LA 1263 | AVRDC | 2.9 ± 0.5 | symptomatic |
| LA 1359 | AVRDC | 2.9 ± 0.5 | symptomatic |
| PI 390688 | AVRDC | 2.9 ± 0.5 | symptomatic |
| PI 407540 | AVRDC | 2.9 ± 0.5 | symptomatic |
| PI 407550 | AVRDC | 2.9 ± 0.5 | symptomatic |
| PI 407539 | AVRDC | 2.9 ± 0.6 | symptomatic |
| PI 407552 | AVRDC | 2.9 ± 0.6 | symptomatic |
| LA 2189 | TGRC | 2.9 ± 0.8 | symptomatic |
| LA 1242 | TGRC | 2.9 ± 0.9 | symptomatic |
| CGN14498 | CGN | 3 ± 0 | symptomatic |
| LA 0413 | TGRC | 3 ± 0 | symptomatic |
| (*Continued on next page*) | | | |

**Supplementary Table S2.** Average disease severity index ratings of *Solanum* spp. accessions upon natural infection with Tomato Yellow Leaf Curl Disease at the Institute of Vegetables and Flowers, Chinese Academy of Agricultural Sciences, Beijing.

| ***Solanum* spp. accession^a^** | **Source^b^** | **Average**  **DSI^c^** | **Phenotype^d^** |
| --- | --- | --- | --- |
| *Solanum pimpinellifolium* |  |  |  |
| LA 1601 | TGRC | 3 ± 0 | symptomatic |
| LA 1604 | TGRC | 3 ± 0 | symptomatic |
| LA 1608 | TGRC | 3 ± 0 | symptomatic |
| LA 1836 | TGRC | 3 ± 0 | symptomatic |
| VI030600 | AVRDC | 3 ± 0 | symptomatic |
| PI 212444 | AVRDC | 3 ± 0.2 | symptomatic |
| VI046131 | AVRDC | 3 ± 0.2 | symptomatic |
| PI 370093 | AVRDC | 3 ± 0.3 | symptomatic |
| LA 0384 | AVRDC | 3 ± 0.4 | symptomatic |
| PI 390697 | AVRDC | 3 ± 0.4 | symptomatic |
| VI040706 | AVRDC | 3 ± 0.4 | symptomatic |
| LA 2966 | TGRC | 3 ± 0.5 | symptomatic |
| PI 390711 | AVRDC | 3 ± 0.5 | symptomatic |
| PI 407556 | AVRDC | 3 ± 0.5 | symptomatic |
| PI 407558 | AVRDC | 3 ± 0.5 | symptomatic |
| LA 1682 | TGRC | 3 ± 0.6 | symptomatic |
| LA 1838 | TGRC | 3 ± 0.6 | symptomatic |
| PI 126939 | AVRDC | 3 ± 0.6 | symptomatic |
| PI 407545 | AVRDC | 3 ± 0.6 | symptomatic |
| VI030544 | AVRDC | 3 ± 0.6 | symptomatic |
| LA 1471 | TGRC | 3 ± 0.7 | symptomatic |
| PI 390705 | AVRDC | 3 ± 0.7 | symptomatic |
| PI 407554 | AVRDC | 3 ± 0.7 | symptomatic |
| LA 1587 | TGRC | 3 ± 1.1 | symptomatic |
| LA 1660 | TGRC | 3 ± 1.2 | symptomatic |
| LA 1582 | TGRC | 3 ± 1.3 | symptomatic |
| LA 2645 | TGRC | 3 ± 1.4 | symptomatic |
| PI 390519 | AVRDC | 3 ± 1.4 | symptomatic |
| LA 1678 | TGRC | 3 ± 1.5 | symptomatic |
| LA 1628 | TGRC | 3 ± 1.7 | symptomatic |
| CGN15812 | CGN | 3.1 ± 0.2 | symptomatic |
| LA 2398 | TGRC | 3.1 ± 0.2 | symptomatic |
| PI 212408 | AVRDC | 3.1 ± 0.2 | symptomatic |
| PI 224710 | AVRDC | 3.1 ± 0.2 | symptomatic |
| PI 127805 | AVRDC | 3.1 ± 0.3 | symptomatic |
| PI 270446 | AVRDC | 3.1 ± 0.3 | symptomatic |
| VI030601 | AVRDC | 3.1 ± 0.3 | symptomatic |
| LA 2390 | TGRC | 3.1 ± 0.4 | symptomatic |
| LA 2831 | TGRC | 3.1 ± 0.4 | symptomatic |
| PI 126927 | AVRDC | 3.1 ± 0.4 | symptomatic |
| LA 1255 | AVRDC | 3.1 ± 0.5 | symptomatic |
| PI 126954 | AVRDC | 3.1 ± 0.5 | symptomatic |
| (*Continued on next page*) | | | |

**Supplementary Table S2.** Average disease severity index ratings of *Solanum* spp. accessions upon natural infection with Tomato Yellow Leaf Curl Disease at the Institute of Vegetables and Flowers, Chinese Academy of Agricultural Sciences, Beijing.

| ***Solanum* spp. accession^a^** | **Source^b^** | **Average**  **DSI^c^** | **Phenotype^d^** |
| --- | --- | --- | --- |
| *Solanum pimpinellifolium* |  |  |  |
| PI 407547 | AVRDC | 3.1 ± 0.5 | symptomatic |
| VI030545 | AVRDC | 3.1 ± 0.5 | symptomatic |
| PI 407541 | AVRDC | 3.1 ± 0.6 | symptomatic |
| PI 407551 | AVRDC | 3.1 ± 0.6 | symptomatic |
| LA 2112 | TGRC | 3.1 ± 0.7 | symptomatic |
| LA 0391 | TGRC | 3.1 ± 0.8 | symptomatic |
| LA 0397 | TGRC | 3.1 ± 0.8 | symptomatic |
| LA 1585 | TGRC | 3.1 ± 0.8 | symptomatic |
| LA 1858 | TGRC | 3.1 ± 0.8 | symptomatic |
| To-937 |  | 3.1 ± 0.8 | symptomatic |
| PI 407548 | AVRDC | 3.1 ± 0.9 | symptomatic |
| PI 126936 | AVRDC | 3.1 ± 1 | symptomatic |
| LA 1637 | TGRC | 3.1 ± 1.1 | symptomatic |
| LA 1332 | TGRC | 3.1 ± 1.2 | symptomatic |
| LA 1591 | TGRC | 3.1 ± 1.2 | symptomatic |
| PI 270440 | AVRDC | 3.2 ± 0.2 | symptomatic |
| LA 2585 | TGRC | 3.2 ± 0.3 | symptomatic |
| PI 126931 | AVRDC | 3.2 ± 0.3 | symptomatic |
| PI 224709 | AVRDC | 3.2 ± 0.3 | symptomatic |
| PI 375937 | AVRDC | 3.2 ± 0.3 | symptomatic |
| CGN15913 | CGN | 3.2 ± 0.4 | symptomatic |
| LA 1933 | TGRC | 3.2 ± 0.4 | symptomatic |
| LA 1993 | TGRC | 3.2 ± 0.4 | symptomatic |
| LA 4138 | TGRC | 3.2 ± 0.4 | symptomatic |
| PI 270441 | AVRDC | 3.2 ± 0.4 | symptomatic |
| PI 270447 | AVRDC | 3.2 ± 0.4 | symptomatic |
| PI 270448 | AVRDC | 3.2 ± 0.4 | symptomatic |
| PI 270449 | AVRDC | 3.2 ± 0.4 | symptomatic |
| LA 1258 | AVRDC | 3.2 ± 0.5 | symptomatic |
| LA 1562 | TGRC | 3.2 ± 0.5 | symptomatic |
| PI 124039 | AVRDC | 3.2 ± 0.5 | symptomatic |
| PI 270442 | AVRDC | 3.2 ± 0.5 | symptomatic |
| PI 390693 | AVRDC | 3.2 ± 0.5 | symptomatic |
| LA 1651 | TGRC | 3.2 ± 0.6 | symptomatic |
| PI 126941 | AVRDC | 3.2 ± 0.6 | symptomatic |
| PI 212442 | AVRDC | 3.2 ± 0.6 | symptomatic |
| PI 346340 | AVRDC | 3.2 ± 0.6 | symptomatic |
| PI 432362 | AVRDC | 3.2 ± 0.6 | symptomatic |
| LA 0114 | TGRC | 3.2 ± 0.7 | symptomatic |
| LA 1874 | TGRC | 3.2 ± 0.7 | symptomatic |
| PI 126436 | AVRDC | 3.2 ± 0.8 | symptomatic |
| PI 432363 | AVRDC | 3.2 ± 0.8 | symptomatic |
| (*Continued on next page*) | | | |

**Supplementary Table S2.** Average disease severity index ratings of *Solanum* spp. accessions upon natural infection with Tomato Yellow Leaf Curl Disease at the Institute of Vegetables and Flowers, Chinese Academy of Agricultural Sciences, Beijing.

| ***Solanum* spp. accession^a^** | **Source^b^** | **Average**  **DSI^c^** | **Phenotype^d^** |
| --- | --- | --- | --- |
| *Solanum pimpinellifolium* |  |  |  |
| LA 2652 | TGRC | 3.2 ± 1.4 | symptomatic |
| LA 2097 | TGRC | 3.3 ± 0.3 | symptomatic |
| PI 263589 | AVRDC | 3.3 ± 0.3 | symptomatic |
| LA 1384 | TGRC | 3.3 ± 0.4 | symptomatic |
| PI 128639 | AVRDC | 3.3 ± 0.4 | symptomatic |
| PI 270453 | AVRDC | 3.3 ± 0.4 | symptomatic |
| PI 344102 | AVRDC | 3.3 ± 0.4 | symptomatic |
| PI 407538 | AVRDC | 3.3 ± 0.4 | symptomatic |
| VI009103 | AVRDC | 3.3 ± 0.4 | symptomatic |
| VI010049 | AVRDC | 3.3 ± 0.4 | symptomatic |
| VI030543 | AVRDC | 3.3 ± 0.4 | symptomatic |
| LA 0412 | TGRC | 3.3 ± 0.5 | symptomatic |
| LA 1572 | TGRC | 3.3 ± 0.5 | symptomatic |
| LA 1719 | TGRC | 3.3 ± 0.5 | symptomatic |
| LA 2348 | TGRC | 3.3 ± 0.5 | symptomatic |
| LA 2853 | TGRC | 3.3 ± 0.5 | symptomatic |
| PI 212441 | AVRDC | 3.3 ± 0.5 | symptomatic |
| PI 407549 | AVRDC | 3.3 ± 0.5 | symptomatic |
| LA 1588 | AVRDC | 3.3 ± 0.6 | symptomatic |
| LA 1864 | TGRC | 3.3 ± 0.6 | symptomatic |
| PI 212409 | AVRDC | 3.3 ± 0.6 | symptomatic |
| PI 306216 | AVRDC | 3.3 ± 0.6 | symptomatic |
| PI 407535 | AVRDC | 3.3 ± 0.6 | symptomatic |
| LA 1342 | TGRC | 3.3 ± 0.7 | symptomatic |
| LA 1573 | TGRC | 3.3 ± 0.7 | symptomatic |
| PI 129062 | AVRDC | 3.3 ± 0.7 | symptomatic |
| PI 212443 | AVRDC | 3.3 ± 0.7 | symptomatic |
| LA 1343 | TGRC | 3.3 ± 0.8 | symptomatic |
| LA 1685 | TGRC | 3.3 ± 0.8 | symptomatic |
| LA 2915 | TGRC | 3.3 ± 0.8 | symptomatic |
| LA 2653 | TGRC | 3.3 ± 1 | symptomatic |
| LA 1577 | TGRC | 3.3 ± 1.5 | symptomatic |
| CGN14352 | CGN | 3.4 ± 0.3 | symptomatic |
| LA 2832 | TGRC | 3.4 ± 0.4 | symptomatic |
| PI 205009 | AVRDC | 3.4 ± 0.4 | symptomatic |
| PI 270450 | AVRDC | 3.4 ± 0.4 | symptomatic |
| PI 274174 | AVRDC | 3.4 ± 0.4 | symptomatic |
| VI007167 | AVRDC | 3.4 ± 0.4 | symptomatic |
| CGN14353 | CGN | 3.4 ± 0.5 | symptomatic |
| LA 1260 | AVRDC | 3.4 ± 0.5 | symptomatic |
| LA 1514 | TGRC | 3.4 ± 0.5 | symptomatic |
| LA 1593 | TGRC | 3.4 ± 0.5 | symptomatic |
| (*Continued on next page*) | | | |

**Supplementary Table S2.** Average disease severity index ratings of *Solanum* spp. accessions upon natural infection with Tomato Yellow Leaf Curl Disease at the Institute of Vegetables and Flowers, Chinese Academy of Agricultural Sciences, Beijing.

| ***Solanum* spp. accession^a^** | **Source^b^** | **Average**  **DSI^c^** | **Phenotype^d^** |
| --- | --- | --- | --- |
| *Solanum pimpinellifolium* |  |  |  |
| LA 1638 | TGRC | 3.4 ± 0.5 | symptomatic |
| LA 1872 | TGRC | 3.4 ± 0.5 | symptomatic |
| LA 2181 | TGRC | 3.4 ± 0.5 | symptomatic |
| LA 2412 | TGRC | 3.4 ± 0.5 | symptomatic |
| LA 2425 | TGRC | 3.4 ± 0.5 | symptomatic |
| PI 126430 | AVRDC | 3.4 ± 0.5 | symptomatic |
| PI 126432 | AVRDC | 3.4 ± 0.5 | symptomatic |
| PI 126925 | AVRDC | 3.4 ± 0.5 | symptomatic |
| PI 127833 | AVRDC | 3.4 ± 0.5 | symptomatic |
| PI 230327 | AVRDC | 3.4 ± 0.5 | symptomatic |
| PI 270439 | AVRDC | 3.4 ± 0.5 | symptomatic |
| PI 270445 | AVRDC | 3.4 ± 0.5 | symptomatic |
| PI 270451 | AVRDC | 3.4 ± 0.5 | symptomatic |
| PI 303662 | AVRDC | 3.4 ± 0.5 | symptomatic |
| PI 309907 | AVRDC | 3.4 ± 0.5 | symptomatic |
| LA 1466 | TGRC | 3.4 ± 0.6 | symptomatic |
| PI 126433 | AVRDC | 3.4 ± 0.6 | symptomatic |
| PI 344103 | AVRDC | 3.4 ± 0.6 | symptomatic |
| PI 407534 | AVRDC | 3.4 ± 0.6 | symptomatic |
| LA 1645 | TGRC | 3.4 ± 0.7 | symptomatic |
| LA 1684 | TGRC | 3.4 ± 0.7 | symptomatic |
| LA 1923 | TGRC | 3.4 ± 0.7 | symptomatic |
| LA 2659 | TGRC | 3.4 ± 0.7 | symptomatic |
| LA 2914A | TGRC | 3.4 ± 0.8 | symptomatic |
| LA 1610 | TGRC | 3.4 ± 0.9 | symptomatic |
| LA 1592 | TGRC | 3.4 ± 1.3 | symptomatic |
| LA 1547 | TGRC | 3.5 ± 0 | symptomatic |
| LA 2903 | TGRC | 3.5 ± 0 | symptomatic |
| LA 1259 | AVRDC | 3.5 ± 0.4 | symptomatic |
| LA 1594 | TGRC | 3.5 ± 0.4 | symptomatic |
| LA 1828 | TGRC | 3.5 ± 0.4 | symptomatic |
| LA 2576 | TGRC | 3.5 ± 0.4 | symptomatic |
| PI 110595 | AVRDC | 3.5 ± 0.4 | symptomatic |
| PI 112215 | AVRDC | 3.5 ± 0.4 | symptomatic |
| PI 270443 | AVRDC | 3.5 ± 0.4 | symptomatic |
| PI 279373 | AVRDC | 3.5 ± 0.4 | symptomatic |
| PI 407533 | AVRDC | 3.5 ± 0.4 | symptomatic |
| LA 0420 | TGRC | 3.5 ± 0.5 | symptomatic |
| LA 1416SAL 504 | AVRDC | 3.5 ± 0.5 | symptomatic |
| LA 1472 | TGRC | 3.5 ± 0.5 | symptomatic |
| LA 1611 | TGRC | 3.5 ± 0.5 | symptomatic |
| LA 1867 | TGRC | 3.5 ± 0.5 | symptomatic |
| (*Continued on next page*) | | | |

**Supplementary Table S2.** Average disease severity index ratings of *Solanum* spp. accessions upon natural infection with Tomato Yellow Leaf Curl Disease at the Institute of Vegetables and Flowers, Chinese Academy of Agricultural Sciences, Beijing.

| ***Solanum* spp. accession^a^** | **Source^b^** | **Average**  **DSI^c^** | **Phenotype^d^** |
| --- | --- | --- | --- |
| *Solanum pimpinellifolium* |  |  |  |
| LA 2170 | TGRC | 3.5 ± 0.5 | symptomatic |
| LA 2179 | TGRC | 3.5 ± 0.5 | symptomatic |
| LA 2340 | TGRC | 3.5 ± 0.5 | symptomatic |
| LA 2345 | TGRC | 3.5 ± 0.5 | symptomatic |
| PI 126933 | AVRDC | 3.5 ± 0.5 | symptomatic |
| PI 126938 | AVRDC | 3.5 ± 0.5 | symptomatic |
| PI 126952 | AVRDC | 3.5 ± 0.5 | symptomatic |
| PI 126953 | AVRDC | 3.5 ± 0.5 | symptomatic |
| PI 270452 | AVRDC | 3.5 ± 0.5 | symptomatic |
| LA 1612 | TGRC | 3.5 ± 0.6 | symptomatic |
| LA 1631 | TGRC | 3.5 ± 0.6 | symptomatic |
| LA 1870 | TGRC | 3.5 ± 0.6 | symptomatic |
| PI 127806 | AVRDC | 3.5 ± 0.6 | symptomatic |
| LA 1584 ^e^ | TGRC | 3.5 ± 0.7 | symptomatic |
| LA 1661 | TGRC | 3.5 ± 0.8 | symptomatic |
| LA 2178 | TGRC | 3.5 ± 0.8 | symptomatic |
| LA 1263SAL 345 | AVRDC | 3.6 ± 0.3 | symptomatic |
| LA 1825 | TGRC | 3.6 ± 0.3 | symptomatic |
| PI 270444 | AVRDC | 3.6 ± 0.3 | symptomatic |
| LA 2180 | TGRC | 3.6 ± 0.4 | symptomatic |
| PI 126924 | AVRDC | 3.6 ± 0.4 | symptomatic |
| PI 212445 | AVRDC | 3.6 ± 0.4 | symptomatic |
| VI030546 | AVRDC | 3.6 ± 0.4 | symptomatic |
| LA 0373 | TGRC | 3.6 ± 0.5 | symptomatic |
| LA 1596 | TGRC | 3.6 ± 0.5 | symptomatic |
| LA 2149 | TGRC | 3.6 ± 0.5 | symptomatic |
| LA 2187 | TGRC | 3.6 ± 0.5 | symptomatic |
| LA 2833 | TGRC | 3.6 ± 0.5 | symptomatic |
| PI 126947 | AVRDC | 3.6 ± 0.5 | symptomatic |
| PI 193408 | AVRDC | 3.6 ± 0.5 | symptomatic |
| PI 79532 | CGN | 3.6 ± 0.5 | symptomatic |
| LA 1679 | TGRC | 3.6 ± 0.9 | symptomatic |
| LA 1860 | TGRC | 3.7 ± 0.4 | symptomatic |
| PI 127807 | AVRDC | 3.7 ± 0.4 | symptomatic |
| PI 340905 | AVRDC | 3.7 ± 0.4 | symptomatic |
| LA 1634 | TGRC | 3.7 ± 0.5 | symptomatic |
| LA 1848 | TGRC | 3.7 ± 0.5 | symptomatic |
| LA 2184 | TGRC | 3.7 ± 0.5 | symptomatic |
| LA 1246 | TGRC | 3.7 ± 0.6 | symptomatic |
| LA 1520 | TGRC | 3.7 ± 0.6 | symptomatic |
| LA 1575 | TGRC | 3.7 ± 0.6 | symptomatic |
| LA 1633 | TGRC | 3.7 ± 0.6 | symptomatic |
| (*Continued on next page*) | | | |

**Supplementary Table S2.** Average disease severity index ratings of *Solanum* spp. accessions upon natural infection with Tomato Yellow Leaf Curl Disease at the Institute of Vegetables and Flowers, Chinese Academy of Agricultural Sciences, Beijing.

| ***Solanum* spp. accession^a^** | **Source^b^** | **Average**  **DSI^c^** | **Phenotype^d^** |
| --- | --- | --- | --- |
| *Solanum pimpinellifolium* |  |  |  |
| LA 2646 | TGRC | 3.7 ± 0.6 | symptomatic |
| LA 1670 | TGRC | 3.7 ± 0.8 | symptomatic |
| LA 1521 | TGRC | 3.8 ± 0.3 | symptomatic |
| LA 1921 | TGRC | 3.8 ± 0.3 | symptomatic |
| LA 2391 | TGRC | 3.8 ± 0.3 | symptomatic |
| LA 1280 | TGRC | 3.8 ± 0.4 | symptomatic |
| LA 1341 | TGRC | 3.8 ± 0.4 | symptomatic |
| LA 1375 | TGRC | 3.8 ± 0.4 | symptomatic |
| LA 1382SAL 466 | AVRDC | 3.8 ± 0.4 | symptomatic |
| LA 2346 | TGRC | 3.8 ± 0.4 | symptomatic |
| LA 2628 | TGRC | 3.8 ± 0.4 | symptomatic |
| LA 2866 | TGRC | 3.8 ± 0.4 | symptomatic |
| LA 2904 | TGRC | 3.8 ± 0.4 | symptomatic |
| LA 3123 | TGRC | 3.8 ± 0.4 | symptomatic |
| LA 3468 | TGRC | 3.8 ± 0.4 | symptomatic |
| CGN23962 | CGN | 3.8 ± 0.5 | symptomatic |
| LA 0121 | TGRC | 3.8 ± 0.5 | symptomatic |
| LA 0480 | TGRC | 3.8 ± 0.5 | symptomatic |
| LA 1831 | TGRC | 3.8 ± 0.5 | symptomatic |
| LA 1720 | TGRC | 3.8 ± 0.6 | symptomatic |
| LA 1866 | TGRC | 3.8 ± 0.6 | symptomatic |
| LA 1835 | TGRC | 3.9 ± 0.2 | symptomatic |
| LA 2183 | TGRC | 3.9 ± 0.2 | symptomatic |
| LA 2851 | TGRC | 3.9 ± 0.2 | symptomatic |
| LA 1629 | TGRC | 3.9 ± 0.3 | symptomatic |
| VI005513 | AVRDC | 3.9 ± 0.3 | symptomatic |
| LA 0375 | TGRC | 3.9 ± 0.4 | symptomatic |
| LA 0381 | TGRC | 3.9 ± 0.4 | symptomatic |
| LA 0400 | TGRC | 3.9 ± 0.4 | symptomatic |
| LA 0443 | TGRC | 3.9 ± 0.4 | symptomatic |
| LA 1600 | TGRC | 3.9 ± 0.4 | symptomatic |
| LA 1613 | TGRC | 3.9 ± 0.4 | symptomatic |
| LA 1687 | TGRC | 3.9 ± 0.4 | symptomatic |
| LA 1688 | TGRC | 3.9 ± 0.4 | symptomatic |
| LA 1842 | TGRC | 3.9 ± 0.4 | symptomatic |
| CGN14351 | CGN | 4 ± 0 | symptomatic |
| CGN14354 | CGN | 4 ± 0 | symptomatic |
| CGN15398 | CGN | 4 ± 0 | symptomatic |
| CGN15458 | CGN | 4 ± 0 | symptomatic |
| CGN15529 | CGN | 4 ± 0 | symptomatic |
| CGN15809 | CGN | 4 ± 0 | symptomatic |
| CGN15813 | CGN | 4 ± 0 | symptomatic |
| (*Continued on next page*) | | | |

**Supplementary Table S2.** Average disease severity index ratings of *Solanum* spp. accessions upon natural infection with Tomato Yellow Leaf Curl Disease at the Institute of Vegetables and Flowers, Chinese Academy of Agricultural Sciences, Beijing.

| ***Solanum* spp. accession^a^** | **Source^b^** | **Average**  **DSI^c^** | **Phenotype^d^** |
| --- | --- | --- | --- |
| *Solanum pimpinellifolium* |  |  |  |
| CGN15946 | CGN | 4 ± 0 | symptomatic |
| CGN15948 | CGN | 4 ± 0 | symptomatic |
| CGN15950 | CGN | 4 ± 0 | symptomatic |
| CGN18401 | CGN | 4 ± 0 | symptomatic |
| LA 0369 | TGRC | 4 ± 0 | symptomatic |
| LA 0411 | TGRC | 4 ± 0 | symptomatic |
| LA 0417 | TGRC | 4 ± 0 | symptomatic |
| LA 0418 | TGRC | 4 ± 0 | symptomatic |
| LA 0722 | TGRC | 4 ± 0 | symptomatic |
| LA 0859 | TGRC | 4 ± 0 | symptomatic |
| LA 1236 | TGRC | 4 ± 0 | symptomatic |
| LA 1261 | CGN | 4 ± 0 | symptomatic |
| LA 1262 | TGRC | 4 ± 0 | symptomatic |
| LA 1269 | TGRC | 4 ± 0 | symptomatic |
| LA 1349 | TGRC | 4 ± 0 | symptomatic |
| LA 1428 | TGRC | 4 ± 0 | symptomatic |
| LA 1469 | TGRC | 4 ± 0 | symptomatic |
| LA 1595 | TGRC | 4 ± 0 | symptomatic |
| LA 1636 | TGRC | 4 ± 0 | symptomatic |
| LA 1652 | TGRC | 4 ± 0 | symptomatic |
| LA 1676 | TGRC | 4 ± 0 | symptomatic |
| LA 1690 | TGRC | 4 ± 0 | symptomatic |
| LA 1697 | TGRC | 4 ± 0 | symptomatic |
| LA 1781 | TGRC | 4 ± 0 | symptomatic |
| LA 1810 | TGRC | 4 ± 0 | symptomatic |
| LA 1818 | TGRC | 4 ± 0 | symptomatic |
| LA 1820 | TGRC | 4 ± 0 | symptomatic |
| LA 1845 | TGRC | 4 ± 0 | symptomatic |
| LA 1925 | TGRC | 4 ± 0 | symptomatic |
| LA 1950 | TGRC | 4 ± 0 | symptomatic |
| LA 2000 | TGRC | 4 ± 0 | symptomatic |
| LA 2147 | TGRC | 4 ± 0 | symptomatic |
| LA 2335 | TGRC | 4 ± 0 | symptomatic |
| LA 2347 | TGRC | 4 ± 0 | symptomatic |
| LA 2655 | TGRC | 4 ± 0 | symptomatic |
| LA 2804 | TGRC | 4 ± 0 | symptomatic |
| LA 2805 | TGRC | 4 ± 0 | symptomatic |
| LA 2839 | TGRC | 4 ± 0 | symptomatic |
| LA 2840 | TGRC | 4 ± 0 | symptomatic |
| LA 2854 | TGRC | 4 ± 0 | symptomatic |
| LA 2857 | TGRC | 4 ± 0 | symptomatic |
| LA 2983 | TGRC | 4 ± 0 | symptomatic |
| (*Continued on next page*) | | | |

**Supplementary Table S2.** Average disease severity index ratings of *Solanum* spp. accessions upon natural infection with Tomato Yellow Leaf Curl Disease at the Institute of Vegetables and Flowers, Chinese Academy of Agricultural Sciences, Beijing.

| ***Solanum* spp. accession^a^** | **Source^b^** | **Average**  **DSI^c^** | **Phenotype^d^** |
| --- | --- | --- | --- |
| *Solanum pimpinellifolium* |  |  |  |
| LA 3158 | TGRC | 4 ± 0 | symptomatic |
| LA 3159 | TGRC | 4 ± 0 | symptomatic |
| LA 3160 | TGRC | 4 ± 0 | symptomatic |
| LA 3638 | TGRC | 4 ± 0 | symptomatic |
| LA 3910 | TGRC | 4 ± 0 | symptomatic |
| LA 4431 | TGRC | 4 ± 0 | symptomatic |
| PI 124161 Selection 1-2 | CGN | 4 ± 0 | symptomatic |
| PI 422397 | AVRDC | 4 ± 0 | symptomatic |

^a^ Taxon using the classification system of (Peralta et al., 2008) and the records holding in Tomato Genetics Resource Center (TGRC); Accession numbers were cross-referenced in different germplasm banks, if LA numbers or PI numbers were not available; then CGN numbers or VI numbers respectively corresponding to gene bank in the Netherland (Centre for Genetic Resources, the Netherlands) and AVRDC (World Vegetable Center in Taiwan (previously the Asian Vegetable Research and Development Center) were presented.

^b^ Germplasm bank where each accessions was sourced.

^c^ Results were displayed as Mean DSI ± Standard deviation.

^d^ Phenotype are categorized as symptomatic and symptomless.

^e^ Accessions were included in the 150 Tomato Genome Re-sequencing project (Aflitos et al., 2014). Selected tomato wild accessions represent diversification of genetic variation. For one accession, whitefly resistance reported in *S. pimpinellifolium* accession LA1584 (Firdaus et al., 2012). *S. peruvianum* accession PI 266375 refers to a different accession number as well as different tomato wild species (CGN15820, *S. cheesmaniae*), in the list of selected wild accessions of the 150 Tomato Genome ReSequencing Project. CGN15820 is the accession number published in the genebank, Centre for Genetic Resources, the Netherlands, corresponding to *S. cheesmaniae*. However, for this accession, resequencing data (single nucleotide variation, and INDEL variations) is not available on the genome browser. *S. pennellii* accession LA2172 refers to a different accession number (LYC 1831), in the list of selected wild accessions of the 150 Tomato Genome ReSequencing Project. LYC 1831 is the accession number published in the genebank, IPK Gatersleben.
