# Supplementary material for: Resistance to Tomato Yellow Leaf Curl Virus in Tomato Germplasm
Source: Front Plant Sci. 2018 Aug 20;9:1198. doi: 10.3389/fpls.2018.01198 (PMC6110163; doi:10.3389/fpls.2018.01198)
Supplement: TABLE S3 — Wild tomato accessions displaying variation in phenotypic responses upon natural infection with tomato yellow leaf curl disease at the Institute of Vegetables and Flowers, Chinese Academy of Agricultural Sciences, Beijing. [file Table_3.docx]

**Supplementary Table S3.** Wild tomato accessions displaying variation in phenotypic responses upon natural infection with Tomato Yellow Leaf Curl Disease at the Institute of Vegetables and Flowers, Chinese Academy of Agricultural Sciences, Beijing

| ***Solanum* spp. accession^a^** | **Total number of**  **tested plants** | **Number of plants assigned to each DSI^b^** | | | | | | | | |
| --- | --- | --- | --- | --- | --- | --- | --- | --- | --- | --- |
|  |  | **0** | **0.5** | **1** | **1.5** | **2** | **2.5** | **3** | **3.5** | **4** |
| *S. arcanum* |  |  |  |  |  |  |  |  |  |  |
| LA2157 ^c^ | 4 | 2 | 0 | 1 | 0 | 0 | 0 | 0 | 0 | 1 |
| LA1346 | 8 | 5 | 0 | 0 | 0 | 1 | 2 | 0 | 0 | 0 |
| LA1708 | 12 | 9 | 0 | 3 | 0 | 0 | 0 | 0 | 0 | 0 |
| LA2185 | 8 | 4 | 0 | 1 | 2 | 1 | 0 | 0 | 0 | 0 |
| LA2334 | 14 | 11 | 0 | 3 | 0 | 0 | 0 | 0 | 0 | 0 |
| *S. chilense* |  |  |  |  |  |  |  |  |  |  |
| LA1782 | 10 | 4 | 0 | 6 | 0 | 0 | 0 | 0 | 0 | 0 |
| LA2754 | 11 | 8 | 0 | 2 | 1 | 0 | 0 | 0 | 0 | 0 |
| CGN15877 | 13 | 10 | 0 | 3 | 0 | 0 | 0 | 0 | 0 | 0 |
| VI031800 | 4 | 3 | 0 | 0 | 1 | 0 | 0 | 0 | 0 | 0 |
| *S. corneliomulleri* |  |  |  |  |  |  |  |  |  |  |
| LA1677 | 6 | 2 | 0 | 4 | 0 | 0 | 0 | 0 | 0 | 0 |
| PI 126448 | 5 | 3 | 0 | 2 | 0 | 0 | 0 | 0 | 0 | 0 |
| LA3219 | 15 | 12 | 0 | 3 | 0 | 0 | 0 | 0 | 0 | 0 |
| LA1294 | 10 | 7 | 0 | 3 | 0 | 0 | 0 | 0 | 0 | 0 |
| LA1744 | 4 | 2 | 0 | 2 | 0 | 0 | 0 | 0 | 0 | 0 |
| LA2721 | 3 | 1 | 0 | 2 | 0 | 0 | 0 | 0 | 0 | 0 |
| LA1944 | 3 | 1 | 0 | 0 | 2 | 0 | 0 | 0 | 0 | 0 |
| LA2724 | 3 | 2 | 0 | 1 | 0 | 0 | 0 | 0 | 0 | 0 |
| CGN14357 | 14 | 12 | 0 | 2 | 0 | 0 | 0 | 0 | 0 | 0 |
| *S. habrochaites* |  |  |  |  |  |  |  |  |  |  |
| LA2314 | 8 | 4 | 0 | 2 | 0 | 2 | 0 | 0 | 0 | 0 |
| CGN15817 | 10 | 9 | 0 | 0 | 1 | 0 | 0 | 0 | 0 | 0 |
| PI126445 | 13 | 7 | 0 | 2 | 0 | 1 | 0 | 3 | 0 | 0 |
| LA1341 | 7 | 3 | 0 | 2 | 0 | 2 | 0 | 0 | 0 | 0 |
| 2H04 | 3/10 | 3 | 0 | 4 | 0 | 1 | 0 | 2 | 0 | 0 |
| (*Continued on next page*) | | | | | | | | | | |

**Supplementary Table S3.** Wild tomato accessions displaying variation in phenotypic responses upon natural infection with Tomato Yellow Leaf Curl Disease at the Institute of Vegetables and Flowers, Chinese Academy of Agricultural Sciences, Beijing

| ***Solanum* spp. accession^a^** | **Total number of**  **tested plants** | **Number of plants assigned to each DSI^b^** | | | | | | | | |
| --- | --- | --- | --- | --- | --- | --- | --- | --- | --- | --- |
|  |  | **0** | **0.5** | **1** | **1.5** | **2** | **2.5** | **3** | **3.5** | **4** |
| *S. habrochaites* |  |  |  |  |  |  |  |  |  |  |
| H20H | 3/9 | 3 | 0 | 4 | 0 | 2 | 0 | 1 | 0 | 0 |
| H305 | 5/10 | 5 | 0 | 2 | 0 | 1 | 0 | 1 | 0 | 1 |
| 2H02 | 3/6 | 3 | 0 | 1 | 0 | 1 | 0 | 1 | 0 | 0 |
| HM04 | 7/9 | 7 | 0 | 1 | 0 | 0 | 0 | 1 | 0 | 0 |
| LA1353 | 5/9 | 5 | 0 | 1 | 0 | 2 | 0 | 1 | 0 | 0 |
| PI247087 | 3/8 | 3 | 0 | 1 | 0 | 2 | 0 | 1 | 0 | 1 |
| LA1392 | 2/9 | 7 | 0 | 2 | 0 | 0 | 0 | 0 | 0 | 0 |
| *S. pennellii* |  |  |  |  |  |  |  |  |  |  |
| LA1732 | 1/2 | 1 | 0 | 0 | 0 | 0 | 0 | 0 | 0 | 1 |
| LA1282 | 1/4 | 1 | 0 | 2 | 0 | 0 | 0 | 1 | 0 | 0 |
| *S. peruvianum* |  |  |  |  |  |  |  |  |  |  |
| PI 128650-6Y-1-12 | 5/8 | 5 | 0 | 3 | 0 | 0 | 0 | 0 | 0 | 0 |
| LA1369 | 2/3 | 2 | 0 | 1 | 0 | 0 | 0 | 0 | 0 | 0 |
| PI 128650 | 8/12 | 8 | 0 | 4 | 0 | 0 | 0 | 0 | 0 | 0 |
| LA3858 | 7/13 | 7 | 0 | 1 | 4 | 1 | 0 | 0 | 0 | 0 |
| LA3853 | 2/4 | 2 | 0 | 2 | 0 | 0 | 0 | 0 | 0 | 0 |
| LA4325 | 2/3 | 2 | 0 | 1 | 0 | 0 | 0 | 0 | 0 | 0 |
| PI 128648 | 2/4 | 2 | 0 | 2 | 0 | 0 | 0 | 0 | 0 | 0 |
| PI 251308 | 3/4 | 3 | 0 | 0 | 0 | 0 | 1 | 0 | 0 | 0 |
| PI 212407 | 3/4 | 3 | 0 | 0 | 0 | 1 | 0 | 0 | 0 | 0 |
| LA1474 | 2/3 | 2 | 0 | 0 | 0 | 0 | 0 | 1 | 0 | 0 |
| LA0153 | 2/3 | 2 | 0 | 1 | 0 | 0 | 0 | 0 | 0 | 0 |
| LA2744 | 2/3 | 2 | 0 | 0 | 0 | 1 | 0 | 0 | 0 | 0 |
| (*Continued on next page*) | | | | | | | | | | |

**Supplementary Table S3.** Wild tomato accessions displaying variation in phenotypic responses upon natural infection with Tomato Yellow Leaf Curl Disease at the Institute of Vegetables and Flowers, Chinese Academy of Agricultural Sciences, Beijing

| ***Solanum* spp. accession^a^** | **Total number of**  **tested plants** | **Number of plants assigned to each DSI^b^** | | | | | | | | |
| --- | --- | --- | --- | --- | --- | --- | --- | --- | --- | --- |
|  |  | **0** | **0.5** | **1** | **1.5** | **2** | **2.5** | **3** | **3.5** | **4** |
| *S. peruvianum* |  |  |  |  |  |  |  |  |  |  |
| LA3640 | 4/8 | 4 | 0 | 0 | 2 | 0 | 2 | 0 | 0 | 0 |
| LA3900 | 4/8 | 4 | 0 | 4 | 0 | 0 | 0 | 0 | 0 | 0 |
| LA2745 | 9/11 | 9 | 0 | 1 | 0 | 1 | 0 | 0 | 0 | 0 |
| CGN15531 | 9/13 | 9 | 0 | 4 | 0 | 0 | 0 | 0 | 0 | 0 |
| PI 126441 | 1/4 | 1 | 0 | 0 | 0 | 1 | 0 | 0 | 0 | 2 |
| PI 126926 | 6/14 | 6 | 0 | 4 | 2 | 2 | 0 | 0 | 0 | 0 |
| CGN14355 | 7/12 | 7 | 0 | 2 | 2 | 1 | 0 | 0 | 0 | 0 |
| CGN14356 | 8/11 | 8 | 0 | 3 | 0 | 0 | 0 | 0 | 0 | 0 |
| (*Continued on next page*) | | | | | | | | | | |

^a^ Taxon using the classification system of (Peralta et al., 2008) and the records holding in Tomato Genetics Resource Center (TGRC); Accession numbers were cross-referenced in different germplasm banks, if LA numbers or PI numbers were not available; then CGN numbers or VI numbers respectively corresponding to gene bank in the Netherland (Centre for Genetic Resources, the Netherlands) and AVRDC (World Vegetable Center in Taiwan (previously the Asian Vegetable Research and Development Center) were presented.

^b^ DSI = Disease Severity Index on 0 (symptomless) - 4 (severe symptom) scale as described in (Friedmann et al., 1998).

^c^ Accessions were included in the 150 Tomato Genome ReSequencing project (Aflitos et al., 2014).
