# Supplementary material for: Resistance to Tomato Yellow Leaf Curl Virus in Tomato Germplasm
Source: Front Plant Sci. 2018 Aug 20;9:1198. doi: 10.3389/fpls.2018.01198 (PMC6110163; doi:10.3389/fpls.2018.01198)
Supplement: TABLE S4 — Polymerase chain reaction detection of tomato yellow leaf curl virus (TYLCV) in wild accessions screened with Agrobacterium-mediated inoculation. Analyses were performed at 55 days post inoculation in young tissues of three tested individuals of each accession. Plant response was evaluated according to the intensity of viral DNA amplification and symptom expression. [file Table_4.docx]

**Supplementary Table S4.** Polymerase chain reaction detection of Tomato yellow leaf curl virus (TYLCV) in wild accessions screened with Agrobacterium-mediated inoculation. Analyses were performed at 55 days post inoculation in young tissues of three tested individuals of each accession. Plant response was evaluated according to the intensity of viral DNA amplification and symptom expression.

| ***Solanum* spp.** | **Accession**  **number** | **Virus**  **detection** | **Disease Severity**  **Index** |
| --- | --- | --- | --- |
| *S. lycopersicoides* | CGN23973 | 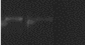 | 0 ± 0 |
| *S. habrochaites* | LA4137 | 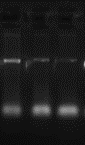 | 0 ± 0 |
| *S. habrochaites* | LA1718 | 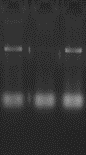 | 0 ± 0 |
| *S. peruvianum* | LA0372 | 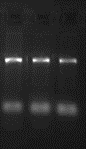 | 0 ± 0 |
| *S. habrochaites* | LA1777 | 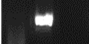 | 0 ± 0 |
| *S. corneliomulleri* | CGN14358 | 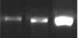 | 0 ± 0 |
| *S. arcanum* | LA2172 | 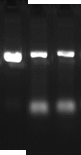 | 0 ± 0 |
| *S. peruvianum* | CGN14355 | 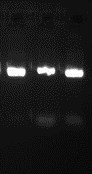 | 0 ± 0 |
| *S. peruvianum* | CGN14356 | 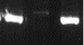 | 0 ± 0 |
| *S. peruvianum* | CGN15531 | 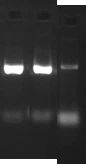 | 0 ± 0 |
| *S. peruvianum* | PI 126928 | 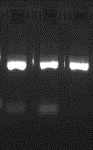 | 0 ± 0 |
| *S. pimpinellifolium* | CGN15528 | 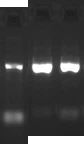 | 0 ± 0 |
| *S. peruvianum* | LA0462 | 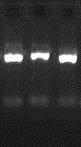 | 0 ± 0 |
| *S. peruvianum* | LA1955 | 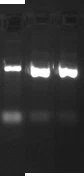 | 0 ± 0 |
| *S. habrochaites* | CGN24035 | 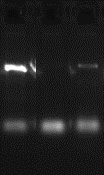 | 4 ± 0 |
| *S. habrochaites* | CGN15790 | 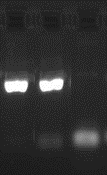 | 4 ± 0 |
| *S. habrochaites* | CGN15879 | 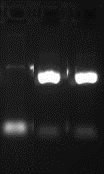 | 4 ± 0 |
| *S. habrochaites* | PI 134417 | 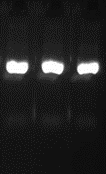 | 4 ± 0 |
| *S. pimpinellifolium* | PI 365960 | 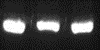 | 4 ± 0 |
| *S. galapagense* | LA1401 | 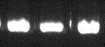 | 4 ± 0 |
| *S. habrochaites* | CGN15792 | 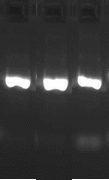 | 4 ± 0 |
| *S. pimpinellifolium* | LA1584 | 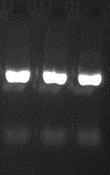 | 4 ± 0 |
