# Supplementary material for: Resistance to Tomato Yellow Leaf Curl Virus in Tomato Germplasm
Source: Front Plant Sci. 2018 Aug 20;9:1198. doi: 10.3389/fpls.2018.01198 (PMC6110163; doi:10.3389/fpls.2018.01198)
Supplement: TABLE S5 — Previous published data on resistant/tolerant and susceptible wild tomato accessions against tomato yellow leaf curl virus complex. [file Table_5.docx]

**Supplementary Table S5.** Previous published data on resistant/ tolerant and susceptible wild tomato accessions against *Tomato Yellow Leaf Curl Virus* complex

| ***Solanum* spp. ^a^**  **Accession/ line Number^b^** | **Viral species^c^** | **Phenotypic response^d^** | **Reference^e^** | |
| --- | --- | --- | --- | --- |
| *Solanum arcanum* | | | | |
| EELM-382 | TYLCV-IL | R | (Tomás et al. 2011) | |
| LA0378 | TYLCV-IL | R | (Pilowsky and Cohen 2000) | |
| LA0385 | TYLCV | R | (Kasrawi et al. 1988) | |
| LA0441 |  | R | (Ji et al. 2007) | |
| LA2185 | TYLCSV-ES, TYLCV-IL and TYLCV-Mld | MR | (Pereira-Carvalho et al. 2010) | |
| LA2553, LA1626, LA2152 | TYLCSV-ES, TYLCV-IL and TYLCV-Mld | R | (Pereira-Carvalho et al. 2010) | |
| *Solanum cheesmaniae* | | | | |
| Breeding line 44 |  |  | (Ji et al. 2007) | |
| LA0166 | TYLCV-EG | R | (El-Dougdoug et al. 2013) | |
| *Solanum chilense* | | | |  |
| EELM-722 | TYLCV-IL | R | (Tomás et al. 2011) | |
| LA1932 | TYLCSV | R | (Jordá et al. 1996; Picó et al. 1998; Pico et al. 1999a) **^e^** | |
| LA1938, LA1968 | TYLCSV | R | (Pico et al. 1999b) | |
| LA1959, LA1960, LA1971 | TYLCSV and TYLCV | R | (Jordá et al. 1996; Pérez de Castro et al. 2005) **^e^** | |
| LA1961 | TYLCSV | R | (Pico et al. 1999b) | |
| LA1963 | TYLCSV | R | (Jordá et al. 1996; Picó et al. 1998) **^e^** | |
| LA1963, LA2747, LA2762 |  | R | (Picó et al. 1996) | |
| LA2774, LA2779 |  | R | (Picó et al. 1996) | |
| LA2884 | TYLCSV and TYLCV | S | (Jordá et al. 1996; Pérez de Castro et al. 2004, 2005; Picó et al. 1998) **^e^** | |
| LA2930, LA2771, LA2759, LA2748 | TYLCSV and TYLCV | R | (Pérez de Castro et al. 2005) **^e^** | |
| LA2981a | TYLCSV and TYLCV | S | (Pérez de Castro et al. 2005) **^e^** | |
| (*Continued on next page*) | | | | |

**Supplementary Table S5.** Previous published data on resistant/ tolerant and susceptible wild tomato accessions against *Tomato Yellow Leaf Curl Virus* complex

| ***Solanum* spp. ^a^**  **Accession/ line Number^b^** | **Viral species^c^** | **Phenotypic response^d^** | **Reference^e^** |
| --- | --- | --- | --- |
| *Solanum chilense* |  |  |  |
| UPV20304, UPV20306, UPV20310, UPV20320, UPV20328, UPV20329, UPV20336 | TYLCSV and TYLCV | R | (Pérez de Castro et al. 2004) **^e^** |
| *Solanum* *chmielewskii* |  |  |  |
| LA1028, LA1306 |  | MR | (de la Peña et al. 2010) |
| *Solanum* *corneliomulleri* | | | |
| B6005 | TYLCV-IN | R | (Banerjee and Kalloo 1987) |
| CGN15803 | TYLCV-EG | S | (El-Dougdoug et al. 2013) |
| CNPH-1033 | TYLCSV-ES, TYLCV-IL/Mld | R | (Pereira-Carvalho et al. 2010) |
| LA0451, LA0366, LA0364 |  | MR | (de la Peña et al. 2010) |
| LA1274, LA1373 | TYLCV | R | (Kasrawi et al. 1988) |
| LA1609, LA1973 | TYLCSV-ES, TYLCV-IL , and TYLCV-Mld | R | (Pereira-Carvalho et al. 2010) |
| *Solanum* *galapagense* |  |  |  |
| LA 1401 | TYLCV-IL | MR | (Vidavski et al. 2008) |
| *Solanum habrochaites* |  |  |  |
| CGN24035 | TYLCV-EG | R | (El-Dougdoug et al. 2013) |
| ECU336, ECU436 | TYLCSV | R | (Picó and Díez 1998) **^e^** |
| EELM-386, EELM-392 | TYLCV-IL | MR segregating | (Tomás et al. 2011) |
| EELM-388, EELM-889 | TYLCV-IL | R | (Tomás et al. 2011) |
| LA0386, LA1777 | TYLCV-IL | R | (Vidavsky and Czosnek 1998) |
| LA0407 |  | MR | (de la Peña et al. 2010) |
| LA1252, LA1295, LA1352, LA1691 |  | R | (Ji et al. 2007) |
| (*Continued on next page*) | | | |

**Supplementary Table S5.** Previous published data on resistant/ tolerant and susceptible wild tomato accessions against *Tomato Yellow Leaf Curl Virus* complex

| ***Solanum* spp. ^a^**  **Accession/ line Number^b^** | **Viral species^c^** | **Phenotypic response^d^** | **Reference^e^** |
| --- | --- | --- | --- |
| *Solanum habrochaites* |  |  |  |
| LA1295, LA1352, LA1252, LA1393, LA1624 | TYLCV | S | (Kasrawi et al. 1988) |
| LA1777 | TYLCSV | S | (Pico et al. 1999a) **^e^** |
| LM-041 | TYLCV-IL | R | (Pereira-Carvalho et al. 2010) |
| PI 126449, PI 129157, PI 134417, PI 134418, PI 199381, PI 365907, LA1265 | TYLCV-IL | S | (Pilowsky and Cohen 2000) |
| PI 127826 | TYLCV-IN | R | (Banerjee and Kalloo 1987) |
| PI 390658, PI 390659, PI 390513 | TYLCV-IL | R | (de la Peña et al. 2010) |
| UPV14260, UPV14356 | TYLCSV | S | (Picó and Díez 1998) **^e^** |
| UPV16910, UPV16911 | TYLCSV and TYLCV | R | (Picó et al. 2000; Soler et al. 2000) **^e^** |
| UPV16929, UPV16924, UPV16928, UPV16931, UPV16933, UPV16934 UPV17041, UPV17046, UPV16914 UPV16916, UPV16920 | TYLCV and TYLCSV | S | (Picó et al. 2000; Soler et al. 2000) **^e^** |
| UPV16930, UPV16941, UPV16918 UPV16919 | TYLCSV | S | (Picó et al. 2000) **^e^** |
| UPV16936 | TYLCV and TYLCSV | S | (Soler et al. 2000) **^e^** |
| *Solanum lycopersicoides* |  |  |  |
| CDP01698, CDP08438, CDP04848, CDP02515, CDP02967, CDP06284, CDP09704 | TYLCV | R | (Pérez de Castro et al. 2010) **^e^** |
| CDP07004, CDP04766, CDP08628, CDP03824, CDP04780 CDP02020 | TYLCV | S | (Pérez de Castro et al. 2010) **^e^** |
| *Solanum lycopersicum var. cerasiforme* |  |  |  |
| ECU464 | TYLCSV | R | (Picó and Díez 1998) **^e^** |
| (*Continued on next page*) | | | |

**Supplementary Table S5.** Previous published data on resistant/ tolerant and susceptible wild tomato accessions against *Tomato Yellow Leaf Curl Virus* complex

| ***Solanum* spp. ^a^**  **Accession/ line Number^b^** | **Viral species^c^** | **Phenotypic response^d^** | **Reference^e^** |
| --- | --- | --- | --- |
| *Solanum neorickii* |  |  |  |
| ECU301 | TYLCSV | R | (Picó and Díez 1998) **^e^** |
| EELM-743 | TYLCV-IL | MR segregating | (Tomás et al. 2011) |
| UPV14227 | TYLCSV | S | (Picó and Díez 1998) **^e^** |
| PI 390677, PI 390678, | TYLCV-IL | S | (Pilowsky and Cohen 2000) |
| *Solanum pennellii* |  |  |  |
| CGN15533( LA0716) | TYLCV-EG | S | (El-Dougdoug et al. 2013) |
| EELM-893 | TYLCV-IL | S | (Tomás et al. 2011) |
| LA1940 |  | R | (de la Peña et al. 2010) |
| UPV16937,UPV16940,UPV16942, UPV16945, UPV17043 | TYLCV and TYLCSV | S | (Picó et al. 2000; Soler et al. 2000) **^e^** |
| UPV16941 | TYLCSV | S | (Picó et al. 2000) **^e^** |
| *Solanum peruvianum* |  |  |  |
| EC 104395 | TYLCV | MR | (Vidavsky et al. 1998) |
| EC 148898, B6002/77, EC148897, B6002 | TYLCV-IN | R | (Banerjee and Kalloo 1987) |
| ECU446 | TYLCSV | R | (Picó and Díez 1998) **^e^** |
| KC-315037, KC-315038, KC-315039, KC-315040, KC-315041 | TYLCV-IL | R | (Azizi et al. 2008) |
| LA0454 | TYLCSV-ES, TYLCV-IL, and TYLCV-Mld | MR | (Pereira-Carvalho et al. 2010) |
| LA0462 | TYLCV-EG | R | (El-Dougdoug et al. 2013) |
| LA0462, LA1333, CMVSel. INRA | TYLCV | R | (Kasrawi et al. 1988) |
| LA0372 | TYLCV | R | (Kasrawi et al. 1988) |
| LA1954 |  | MR | (de la Peña et al. 2010) |
| PI 126431, PI126945 | TYLCV-IL | R | (Pilowsky and Cohen 2000) |
| (*Continued on next page*) | | | |

**Supplementary Table S5.** Previous published data on resistant/ tolerant and susceptible wild tomato accessions against *Tomato Yellow Leaf Curl Virus* complex

| ***Solanum* spp. ^a^**  **Accession/ line Number^b^** | **Viral species^c^** | **Phenotypic response^d^** | **Reference^e^** |
| --- | --- | --- | --- |
| *Solanum peruvianum* | | | |
| PI 126441, PI 212407, PI 251311, LA2770, LA2744 | TYLCSV | S | (Jordá et al. 1996) **^e^** |
| PI 126926, PI 126930, PI 390681 |  | R | (Ji et al. 2007) |
| PI 126929, PI 390682, PI 126944 | TYLCV-IL | S | (Pilowsky and Cohen 2000) |
| PI 126935 | TYLCSV | R | (Jordá et al. 1996; Picó et al. 1998) **^e^** |
| PI 126935 | TYLCV-IL | MR | (Pilowsky and Cohen 1990) |
| PI 126944 | TYLCSV and TYLCV | R | (Pérez de Castro et al. 2004, 2005; Picó et al. 1998; Pico et al. 1999a) **^e^** |
| PI 127830 |  | R | (de la Peña et al. 2010) |
| PI 127831 | TYLCV-IL | MR | (Zakay et al. 1991) |
| PI 143679 | TYLCSV | R | (Jordá et al. 1996; Picó et al. 1998;Pico et al. 1999a)^e^ |
| PI 306811, PI 365951, PI 365952, PI 365953, CNPH-1194, CNPH-0785, LA2744, PI 128660 | TYLCSV-ES, TYLCV-IL, and TYLCV-Mld | R | (Pereira-Carvalho et al. 2010) |
| PI 127832, 78-1556, 81-2274, CMV-INRA | TYLCV-IL | R | (Zakay et al. 1991) |
| UPV12121, PE30 | TYLCSV | S | (Jordá et al. 1996; Picó et al. 1998) **^e^** |
| UPV14367 | TYLCSV | S | (Picó and Díez 1998) **^e^** |
| UPV20196, UPV20345, | TYLCSV and TYLCV | S | (Pérez de Castro et al. 2005) **^e^** |
| UPV20208 | TYLCSV and TYLCV | R | (Pérez de Castro et al. 2005) **^e^** |
| UPV20340, UPV20342 | TYLCSV and TYLCV | S | (Pérez de Castro et al. 2004) **^e^** |
| UPV20344, UPV20349, UPV20197, UPV20311 | TYLCSV and TYLCV | S | (Pérez de Castro et al. 2005) **^e^** |
| UPV20355, UPV20379 | TYLCSV and TYLCV | R | (Pérez de Castro et al. 2004) **^e^** |
| (*Continued on next page*) | | | |

**Supplementary Table S5.** Previous published data on resistant/ tolerant and susceptible wild tomato accessions against *Tomato Yellow Leaf Curl Virus* complex

| ***Solanum* spp. ^a^**  **Accession/ line Number^b^** | **Viral species^c^** | **Phenotypic response^d^** | **Reference^e^** |
| --- | --- | --- | --- |
| *Solanum pimpinellifolium* |  |  |  |
| 69- 187, 75-298 | TYLCV | R | (Zakay et al. 1991) |
| CGN15812 | TYLCV-EG | S | (El-Dougdoug et al. 2013) |
| CIAS 27, PI 126430, PI 126436, PI 126927 | TYLCV-IL | S | (Pilowsky and Cohen 2000) |
| ECU421, | TYLCSV | S | (Picó and Díez 1998) **^e^** |
| EELM-127, EELM-363 | TYLCV-IL | MR | (Tomás et al. 2011) |
| Hirsute-INRA, LA1478 | TYLCV | MR | (Kasrani 1989) |
| LA 1921, EC 65992, PI 205009, Pan American | TYLCV- IN | R | (Banerjee and Kalloo 1987) |
| LA0121 | TYLCV-IL | MR | (Pilowsky and Cohen 1974) |
| LA0373 | TYLCV | MR | (Kasrani 1989) |
| LA0375, LA1335 |  | MR | (de la Peña et al. 2010) |
| LA1478 | TYLCV-IL | S | (Zakay et al. 1991) |
| LA1579, LA1589, |  | MR | (Picó et al. 1996) |
| PI 211840, PI 212408 |  | MR | (Picó et al. 1996) |
| PI 407543, LA1582, PI 407544 |  | MR | (Ji et al. 2007) |
| PI 407555 |  | MR | (Ji et al. 2007) |
| UPV- 16991 | TYLCSV | MR | (Pérez de Castro et al. 2008) |
| UPV16951, UPV16963, UPV16971, UPV16973, UPV17039 | TYLCV and TYLCSV | S | (Soler et al. 2000) **^e^** |
| UPV16953 | TYLCSV | R | (Picó et al. 2000) **^e^** |
| UPV16957, UPV16960 UPV16962, UPV16975, UPV17047 | TYLCSV | S | (Picó et al. 2000) **^e^** |
| UPV16966, UPV16990, UPV16991, UPV17049 | TYLCSV and TYLCV | R | (Picó et al. 2000; Soler et al. 2000) **^e^** |
| (*Continued on next page*) | | | |

**Supplementary Table S5.** Previous published data on resistant/ tolerant and susceptible wild tomato accessions against *Tomato Yellow Leaf Curl Virus* complex

| ***Solanum* spp. ^a^**  **Accession/ line Number^b^** | **Viral species^c^** | **Phenotypic response^d^** | **Reference^e^** |
| --- | --- | --- | --- |
| *Solanum pimpinellifolium* | | | |
| UPV16903, UPV16904 UPV16947 UPV16949 UPV16950, UPV16952  UPV16954, UPV16958, UPV16959 UPV16961, UPV16964, UPV16965  UPV16968, UPV16969 UPV16970 UPV16972 UPV16974, UPV16976 UPV16977, UPV16978 UPV16980 UPV16981 UPV16982, UPV16983 UPV16984, UPV16985 UPV16987 UPV16988, UPV16989, UPV17044 | TYLCV and TYLCSV | S | (Picó et al. 2000; Soler et al. 2000) **^e^** |

^a^ Taxonomy of wild species referred to (Peralta et al. 2008).

^b^ Accession sourced from, EELM accessions: the La Mayora-CSIC seed bank, Spain; UPV, ECU and PE accessions: Genebank of the Institute for the Conservation and Improvement of Biodiversity (COMAV); CGN accessions: Centre for Genetic Resources, The Netherlands; CNPH accessions: the National Center for Vegetable Crops Research (CNPH)/ EMBRAPA (Brasilia-DF, Brazil); INRA accessions: H. Laterrot, INRA, Avignon, France; 69- 187, 75-298, 78-1556, 81-2274, 3407 seeds: from D. Zamir, The Hebrew University of Jerusalem, Rehovot, Israel

^c^ TYLCV (*Tomato yellow leaf curl* *virus*); TYLCSV (*Tomato yellow leaf curl Sardinia virus*); TYLCV-IL (Israel or Mild strain of *Tomato yellow leaf curl* *virus*); TYLCSV-ES (ES strain of *Tomato yellow leaf curl Sardinia virus*); TYLCV-Mld (Mild strain of *Tomato yellow leaf curl virus*); TYLCV-EG (Egyptian isolate of *Tomato yellow leaf curl* *virus*); TYLCV-In (India strain of *Tomato yellow leaf curl* *virus*)

^d^  R: resistant; MR: moderately resistant (with slight symptom or tolerant); S: susceptible

^e^ Resistant and susceptible accessions obtained from nine COMAV publications (Institute for the Conservation and Improvement of Agrodiversity (COMAV), Valencia). One of the resistant accessions (ECU301) was initially tested as *S. peruvianum* (Picó and Díez 1998), but later reclassified as *S. neorickii*.

**References**

Azizi, A., Mozafari, J., and Shams-bakhsh, M. (2008). Phenotypic and molecular screening of tomato germplasm for resistance to *Tomato yellow leaf curl virus*. *Iran. J. Biotechnol*. 6, 199-206.

Banerjee, M. K., and Kalloo, M. K. (1987). Sources and inheritance of resistance to leaf curl virus in *Lycopersicon*. *Theor*. *Appl*. *Genet*. 73, 707-710. doi.org/10.1007/BF00260780

De la Peña, R., Kadirvel, P., Venkatesan, S., Kenyon, L., and Hughes, J. (2010). “Integrated approaches to manage Tomato Yellow Leaf Curl Viruses,” in *Biocatalysis and Biomolecular Engineering*. eds C. T. Hou and J-F. Shaw (Hoboken NJ: John Wiley & Sons), 105-132. doi.org/10.1002/9780470608524.ch8

El-Dougdoug, N. K., Mahfouze, S. A., Ahmed, S. A., Othman, B. A., and Hazaa, M. M. (2013). Identification of biochemical and molecular markers in tomato yellow leaf curl virus resistant tomato species. *Sci*. *Agri*. 2, 46-53.

Ji, Y., Scott, J. W., Hanson, P., Graham, E., and Maxwell, D. P. (2007). “Sources of resistance, inheritance, and location of genetic loci conferring resistance to members of the tomato-infecting begomoviruses,” in *Tomato Yellow Leaf Curl Virus Disease: Management, Molecular Biology, Breeding for Resistance*, ed. H. Czosnek (Dordrecht, The Netherlands: Springer), 343-362. doi.org/10.1007/978-1-4020-4769-5_20

Jordá, C., Picó, B., Díez, M. J., and Nuez, F. (1996). Cribado de germoplasma resistente a TYLCV. Desarrollo de un método de diagnóstico adecuado. *VIII Congreso Nacional de la Sociedad Española de Fitopatología*. Córdoba, Spain. 218.

Kasrawi, M. A., Suwwan, M. A., and Mansour, A. (1988). Sources of resistance to tomato-yellow-leaf-curl-virus (TYLCV) in *Lycopersicon* species. *Euphytica* 37, 61-64. doi.org/10.1007/BF00037224

Kasrawi, M. A. (1989). Inheritance of resistance to tomato yellow leaf curl virus (TYLCV) in *Lycopersicon pimpinellifolium*. *Plant Dis*. 73, 435-437.

Pereira-Carvalho, R. C., Boiteux, L. S., Fonseca, M. E. N., Díaz-Pendón, J. A., Moriones, E., Fernández-Muñoz, R., et al. (2010). Multiple resistance to *Meloidogyne* spp. and bipartite and monopartite *Begomovirus* spp. in wild *Solanum* (*Lycopersicon*) accessions. *Plant Dis*. 94, 179-185. doi.org/10.1094/PDIS-94-2-0179

Pérez de Castro, A., Díez, M. J., and Nuez, F. (2004). Identificación de nuevas fuentes de resistencia al virus del rizado amarillo del tomate (TYLCV). *Actas de Horticultura*. 41, 119-122.

Pérez de Castro, A., Díez, M. J., and Nuez, F. (2005). Caracterización de entradas de Lycopersicon peruvianum y *L. chilense* por su resistencia al *Tomato yellow leaf curl virus* (TYLCV). *Actas Portuguesas de Horticultura*. 8, 48-54.

Pérez de Castro, A., Díez, M. J., and Nuez, F. (2008). Exploiting partial resistance to *Tomato yellow leaf curl virus* derived from *Solanum pimpinellifolium* UPV16991. *Plant Dis*. 92, 1083-1090. doi.org/10.1094/PDIS-92-7-1083

Pérez de Castro, A., Díez, M. J., and Nuez, F. (2010). Resistencia a la enfermedad del rizado amarillo del tomate en la especie silvestre *Solanum lycopersicoides*. *Actas de Horticul*. 55, 169-170.

Picó, B., Díez, M. J., and Nuez, F. (1996). Viral diseases causing the greatest economic losses to the tomato crop. II. The Tomato yellow leaf curl virus - a review. *Sci*. *Hortic*. 67, 151-196. doi.org/10.1016/S0304-4238(96)00945-4

Picó, B., and Díez, M. J. (1998). “Screening *Lycopersicon* spp. for resistance to TYLCV,” in *2^nd^ International Workshop on Bemisia and Geminiviral Disease*, (San Juan, Puerto Rico: USDA-ARS), 43.

Picó, B., Díez, M. J., and Nuez, F. (1998). Evaluation of whitefly-mediated inoculation techniques to screen *Lycopersicon esculentum* and wild relatives for resistance to Tomato yellow leaf curl virus. *Euphytica* 101, 259-271. doi.org/10.1023/A:1018353806051

Picó, B., Ferriol, M., Diez, M., and Nuez, F. (1999a). Cribado de fuentes de resistencia de *Lycopersicon* spp. al *Tomato Yellow Leaf Curl Virus* mediante agroinoculación en disco de hoja. *Actas de Horticultura*. 24, 105-112.

Picó, B., Ferriol, M., Diez, M. J., and Nuez, F. (1999b). Developing tomato breeding lines resistant to tomato yellow leaf curl virus. *Plant Breed*. 118, 537-542. doi.org/10.1046/j.1439-0523.1999.00427.x

Picó, B., Sifres, A., Elía, M., Díez, M. J., and Nuez, F. (2000). Searching for new resistance sources to tomato yellow leaf curl virus within a highly variable wild *Lycopersicon* genetic pool. *Acta Physiol*. *Plant*. 22, 344-350. doi.org/10.1007/s11738-000-0051-0

Pilowsky, M., and Cohen, S. (2000). Screening additional wild tomatoes for resistance to the whitefly-borne *Tomato yellow leaf curl virus*. *Acta Physiol*. *Plant*. 22, 351-353. doi.org/10.1007/s11738-000-0052-z

Soler, S., Pico, B., Sifres, A., Diez, M., De Frutos, R., and Nuez, F. (2000). “Multiple virus resistance in a collection of Lycopersicon spp.,” in Proceedings of the Fifth Congress of the European Foundation for Plant Pathology, (Taormina, Italy: European Foundation for Plant Pathology), 17.

Tomás, D. M., Cañizares, M. C., Abad, J., Fernández-Muñoz, R., and Moriones, E. (2011). Resistance to *Tomato yellow leaf curl virus* accumulation in the tomato wild relative *Solanum habrochaites* associated with the C4 viral protein. *Mol. Plant Microbe Interact*. 24, 849-861. doi.org/10.1094/MPMI-12-10-0291

Vidavsky, F., and Czosnek, H. (1998). Tomato breeding lines resistant and tolerant to tomato yellow leaf curl virus issued from *Lycopersicon hirsutum*. *Phytopathology* 88, 910-914. doi.org/10.1094/PHYTO.1998.88.9.910

Vidavsky, F., Leviatov, S., Milo, J., Rabinowitch, H., Kedar, N., and Czosnek, H. (1998). Response of tolerant breeding lines of tomato, *Lycopersicon esculentum*, originating from three different sources (*L. peruvianum*, *L. pimpinellifolium* and *L. chilense*) to early controlled inoculation by tomato yellow leaf curl virus (TYLCV). *Plant Breed.* 117, 165-169. doi.org/10.1111/j.1439-0523.1998.tb01472.x

Vidavski, F., Czosnek, H., Gazit, S., Levy, D., and Lapidot, M. (2008). Pyramiding of genes conferring resistance to *Tomato yellow leaf curl virus* from different wild tomato species. *Plant Breed*. 127, 625-631. doi.org/10.1111/j.1439-0523.2008.01556.x

Zakay, Y., Navot, N., Zeidan, M., Kedar, N., Rabinowitch, H., Czosnek, H., et al. (1991). Screening *Lycopersicon* accessions for resistance to tomato yellow leaf curl virus: presence of viral DNA and symptom development. *Plant Dis*. 75, 279-281.
